# Skeletal Transformations Observed in the Reaction of a Tricyclic Thymine Nucleoside with Dicarbonyl Compounds

**DOI:** 10.1021/acsomega.4c02553

**Published:** 2024-08-12

**Authors:** María-Cruz Bonache, Elisa G. -Doyagüez, Raúl Benito-Arenas, M. Angeles Bonache, María-Luisa Jimeno, Ana San-Félix

**Affiliations:** † Instituto de Química Médica (CSIC), Juan de la Cierva 3, 28006 Madrid, Spain; ‡ Centro de Química Orgánica “Lora-Tamayo” (CSIC), Juan de la Cierva 3, 28006 Madrid, Spain; § Instituto de Química Orgánica General (CSIC), Juan de la Cierva 3, 28006 Madrid, Spain

## Abstract

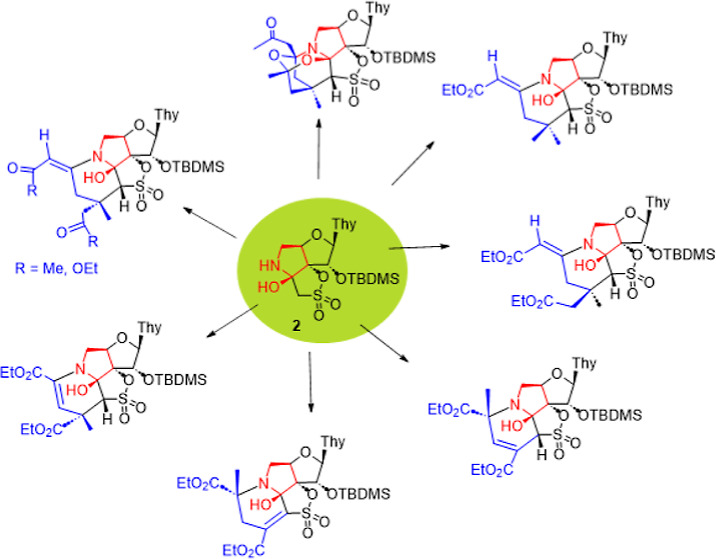

Some intriguing skeletal transformations were observed in the reaction of α-hydroxypyrrolidine thymine nucleoside **2** with different dicarbonyl compounds. In these reactions, unusual ring systems, together with new C–C bonds and stereogenic centers of defined configuration, were formed in a single step. These reactions were initiated by the nucleophilic attack of the NH of the pyrrolidine ring, present on **2**, on one of the carbonyl moieties of a dicarbonyl reagent and seem to proceed through an enamine–iminium mechanism. The present methodology is particularly attractive because no catalyst or aggressive conditions are needed. The new polycyclic nucleosides obtained from **2** can be good scaffolds for diversification. In fact, modification and derivatization can be achieved by performing further chemical transformations of the functional groups present in some of them. This may lead to the formation of new highly functionalized nucleosides. Our results show the high synthetic potential of **2** to construct complex systems in an efficient way. On the other hand, the enamine chemistry involved in the particular reactivity of the α-hydroxy pyrrolidine ring present in **2** has no connection with the nucleobase and could be extended to simple glycosides preserving this essential ring system.

## Introduction

The generation of molecular complexity in a rapid and controlled way is an important aspect of modern synthetic chemistry.^1^ In this respect, reactions in tandem are some of the ways to reach this goal as several bonds can be formed in a single operation under the same reaction conditions.^2−9^

**Figure 1:**
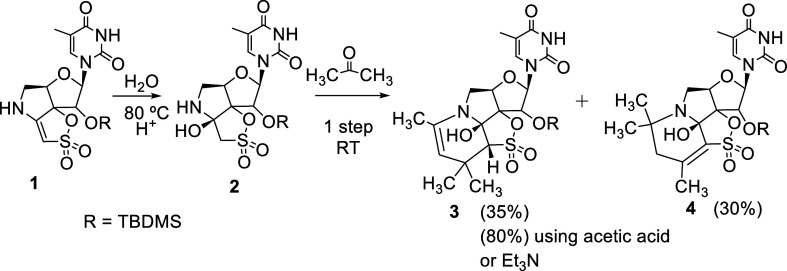
Synthesis of the α-hydroxy pyrrolidine tricyclic nucleoside **2** and its spontaneous reaction with acetone observed in our previous project.

Previously,^10^ we have discovered that the α-hydroxy pyrrolidine tricyclic nucleoside **2** (efficiently obtained from **1**) reacted spontaneously with acetone in a short and efficient manner to afford the highly functionalized polycyclic nucleosides **3** and **4**, with rather unusual molecular skeletons, in a complete regio- and stereoselective way (Figure 1). The reaction involves the spontaneous (noncatalyzed) formation of a novel six-membered ring and three new bonds, two of them carbon–carbon bonds, in a single step (one-pot way). In this previous work, it was clearly demonstrated that the process is initiated by the nucleophilic attack of the NH of the pyrrolidine ring present in **2** on the carbonyl moiety of the acetone to give a carbinolamine intermediate that evolves through iminium/enamine intermediates toward the final compounds **3** and **4**. The scope of this reaction was briefly examined using a small set of ketones: 2-butanone, 3-butanone, and methyl vinyl ketone.^10^ From this study, we concluded that the nature of the ketone (R^1^ COR^2^) is critical for the initiation of the reaction (the attack of the NH on the carbonyl group to give the carbinolamine intermediate).

The main goal of this work is to extend this reaction to dicarbonyl compounds (ketones and esters) with nonadjacent and adjacent carbonyl moieties. These reactions allow the construction, in a single chemical step, of polycyclic nucleosides with unique ring systems. The purpose of this study is not only to determine the structure of these new compounds but also to propose plausible mechanisms for their formation.

## Results and Discussion

### Chemistry

We initiated our study by attempting the reaction of **2** with a 1,3-dicarbonyl ketone (acetylacetone). When compound **2** was treated with this ketone, no reaction was observed at room temperature. However, heating at 80 °C for 24 h afforded two novel compounds, **5** and **6**, that were isolated in 40 and 30% yield, respectively, after purification (Table 1, entry 1).

**Table 1:**
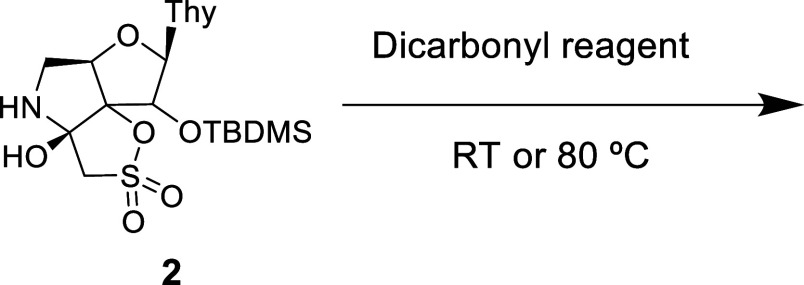

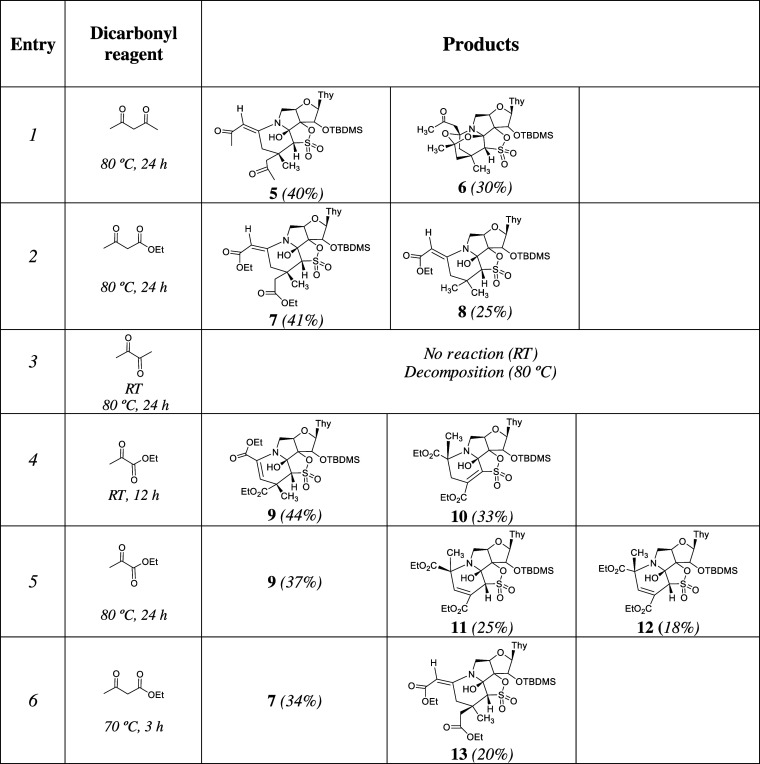
Reaction of **2** with Different 1,3- and 1,2-Dicarbonyl Compounds

On the other hand, when compound **2** was treated at 80 °C for 24 h with a 1,3-dicarbonyl ester (ethyl acetoacetate), a mixture of compounds **7** (41%) and **8** (25%) was obtained (Table 1, entry 2). The reaction was also successful when methyl acetoacetate was used as a reagent. However, in this case, the resulting compounds could not be satisfactorily separated, and their structures could not be unequivocally determined.

Next, we decided to investigate the reaction with 1,2-dicarbonyl compounds (ketones and esters) (Table 1, entries 3 and 4).

When compound **2** was treated with 2,3-butanedione (ketone), no reaction was observed at room temperature. Heating at 80 °C for 24 h afforded complex mixtures of compounds that could not be identified (Table 1, entry 3).

Moreover, the reaction with an ester derivative was studied. Thus, when **2** was reacted with ethyl pyruvate at room temperature for 12 h, a mixture of compounds **9** (44%) and **10** (33%) was obtained (Table 1, entry 4). The reaction was also successful at 80 °C. In this case, besides **9**, which was obtained in 37% yield, two new compounds, **11** (25%) and **12** (18%), were isolated. However, compound **10** was not detected (Table 1, entry 5).

The structures of new compounds **5**–**12** were assigned by NMR and mass spectrometry (MS) studies. The rather unusual molecular skeletons formed in these reactions, together with particularly intriguing results, like the addition of an unusual ring to our precursor system to give **6** or the formation of **8**, which involves a decarboxylation process, encouraged us to study in detail all of these transformations.

#### Mechanistic Considerations

**Scheme 1:**
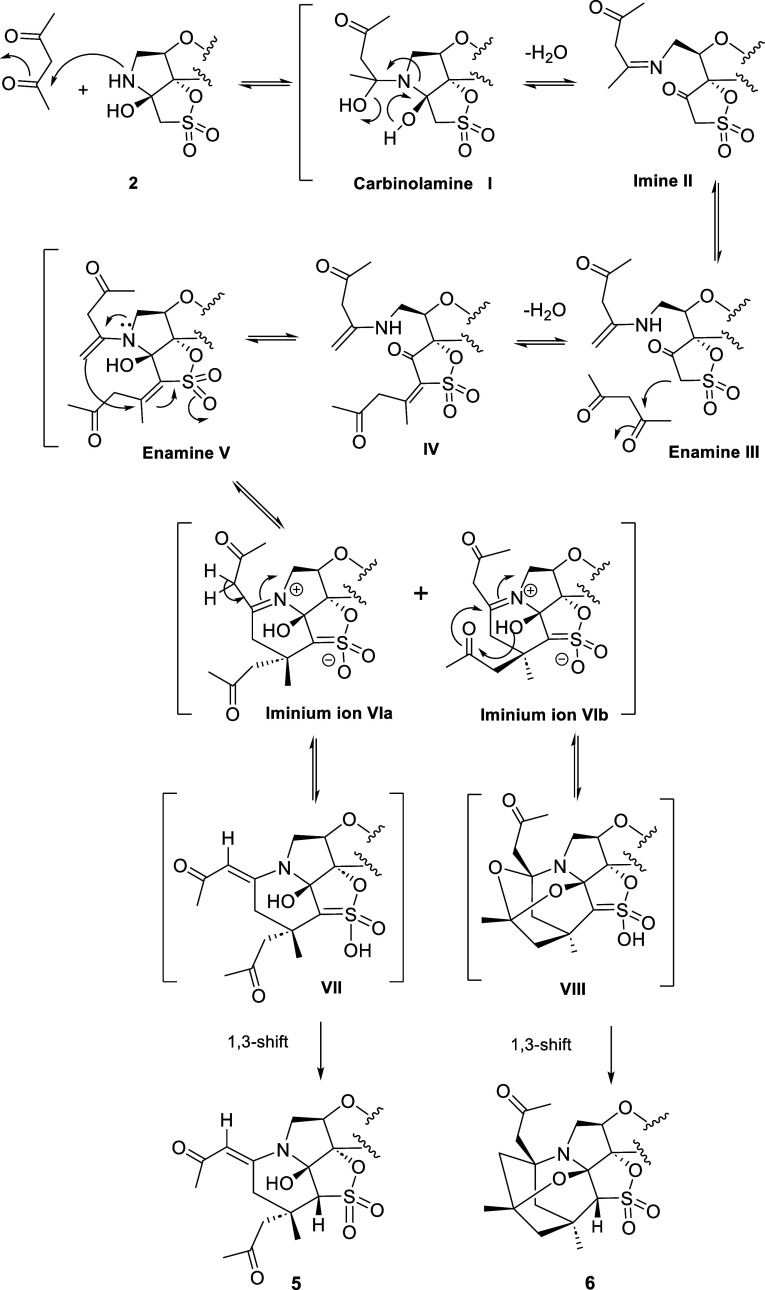
Proposed Evolution of **2** toward **5** and **6**

A plausible mechanism for the formation of compounds **5** and **6** is illustrated in Scheme 1. Based on our previously reported results^10,11^ and on literature precedents concerning iminium- and enamine-based catalysis,^12−25^ we propose an initial step in which the secondary amino group of the constrained pyrrolidine ring of **2** might attack one of the carbonyls of the acetylacetone to generate the carbinolamine intermediate **I**.

The subsequent opening of the pyrrolidine ring might generate imine intermediate **II** and then enamine **III**. This process would afford a β-keto sulfonate, which might readily react with a second molecule of acetylacetone in a Knoevenagel-type condensation due to the increased acidity of the protons adjacent to SO_2_ compared to those of the β-hydroxy sulfonate to afford **IV**. The reclosure of the ring would restore the original stereochemistry to afford intermediate **V**. The subsequent conjugate addition of the enamine to the activated double bond (C=C–SO_2_) might take place from the top (above the plane) or the bottom face (below the plane) of the furanose ring to give the iminium intermediates **VIa** or **VIb**, respectively. Interestingly, a second C–C bond is formed in this reaction, resulting in a new six-membered ring. Our own work,^10^ together with literature precedents showing the participation of enamines in asymmetric conjugate additions to a vinyl sulfone (Michael acceptor), gives strong support to this attack.^21,26−29^

From **VIa**, a proton transfer from the methylene group next to the iminium ion might afford intermediate **VII**, whose subsequent isomerization (1,3 shift) would give the final compound **5**.

Alternatively, from **VIb**, a subsequent attack of the OH on the neighboring carbonyl group might result in a concerted intramolecular cyclization that might give intermediate **VIII**, whose subsequent isomerization would give the final compound **6**. It should be noted that the system is now much more complex than that of the hydroxy tricyclic precursor **2** and has been constructed with complete control of the regio- and stereochemistry.

A remarkable aspect of our mechanistic proposal is that the formation of **6** points to an intramolecular attack of the OH on the CO, which is only possible if both moieties are on the same side of the molecule. However, this attack is not possible when the OH and CO are on opposite sides of the molecule. In this case, compound **5** is formed. We hypothesized that probably the mentioned attack could take place through a hydrogen-bond-assisted ring-closing strategy in which the hemiaminal C–OH bond is selectively weakened to form a relatively acidic proton. In this way, the nucleophilicity of this OH could be enhanced. The activation of molecules through intramolecular hydrogen-bond formation to promote chemical reactions has been reviewed by Fraile and Alemán et al. in 2022, giving strong support for this hypothesis.^30^

All of the proton transfers that take place in the early stages of the formation of **5** and **6** appear to be mediated by the hydroxyl group at the α position of the pyrrolidine ring, which was regarded as crucial for the progress of the reaction. This hypothesis is in agreement with the above-mentioned precedent from our group.^10^

As shown in Table 1 (entry 2), when we conducted the reaction between **2** and an ester derivative, like ethyl acetoacetate, in acetonitrile at 80 °C, a mixture of compounds **7** (41%) and **8** (25%) was obtained.

**Scheme 2:**
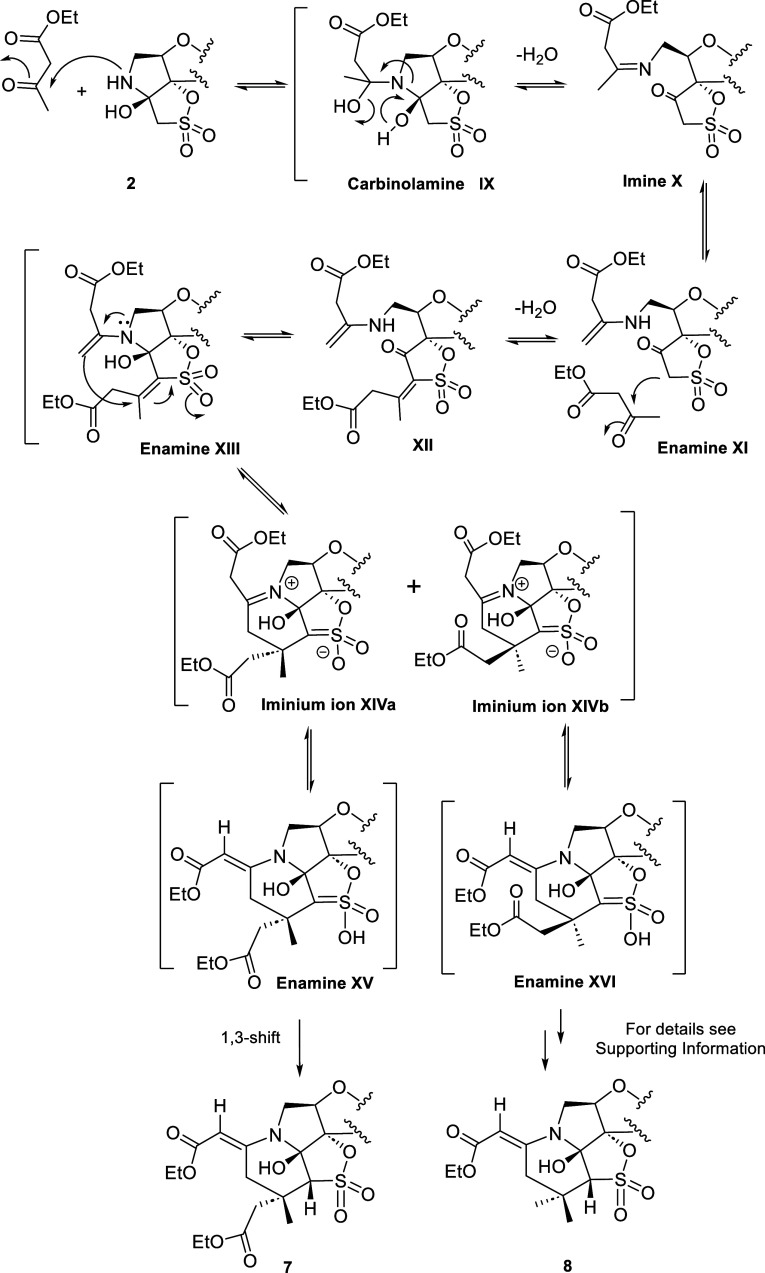
Proposed Evolution of **2** toward **7** and **8**

A plausible mechanism for the formation of compounds **7** and **8** is illustrated in Scheme 2. As shown above, the first step might involve the nucleophilic attack of the amino group of the pyrrolidine ring of **2** at COCH_3_ to form carbinolamine intermediate I**X**. The subsequent opening of the pyrrolidine ring might afford imine intermediate **X** and then enamine **XI**. Subsequent reaction with a second molecule of ethyl acetoacetate, followed by a series of reasonable steps similar to those proposed for **5** and **6** (ring closing and concerted intramolecular cyclization), might afford two possible intermediates **XIVa** (similar to **VIa**) and **XIVb** (similar to **VIb**), with the methyl group at the top or bottom face of the furanose ring, respectively. Next, intermediate **XIVa** could follow a pathway similar to the above proposed for **VIa** to give the final compound **7**, in which the methyl moiety is at the top face of the furanose ring (Scheme 2, left side). However, intermediate **XIVb**, in which the methyl moiety is at the opposite face (bottom), should evolve in a different way to give final compound **8** (Scheme 2, right side). In this transformation, an intriguing and not obvious decarboxylation reaction took place. A possible pathway to explain this transformation is proposed in the Supporting Information (Scheme S1).

With the aim to shed some light about the decarboxylation process, we decided to shorten the reaction time and to decrease the temperature. Thus, a solution of **2** in acetonitrile was allowed to react with ethyl acetoacetate at 70 °C (instead of 80 °C) for 3 h (instead of 24 h). Under these conditions, no traces of **8** were isolated. Instead, a new compound (**13**), whose structure is depicted in Table 1 (entry 6), was formed in a 20% yield. Moreover, compound **7** was also obtained in 34% yield.

**Figure 2:**
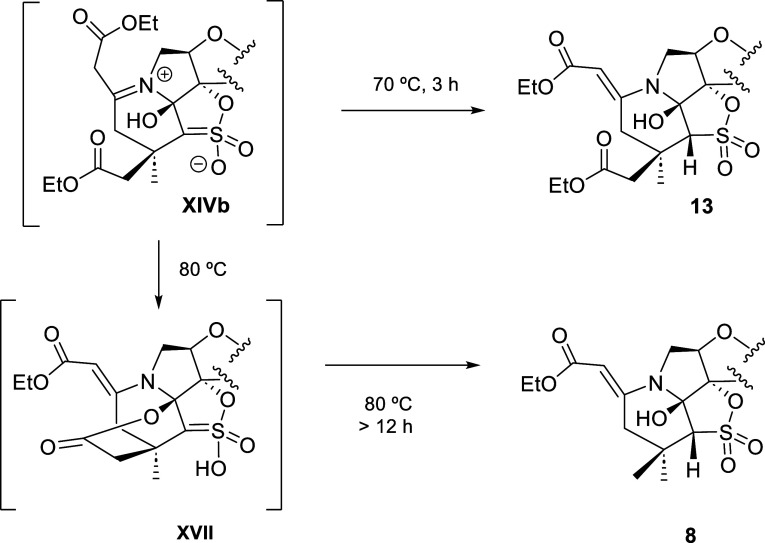
Evolution of **XIVb** toward **13** or **8**.

Isolation of **13**, in which the CH_2_CO_2_Et moiety is on the same side as the OH and thymine (top face of the furanose ring), provided strong support for the participation of intermediate **XIVb** in the formation of **8** (Scheme and Figure 2). This intermediate might evolve toward **13** through imine–enamine equilibrium and SO_2_ isomerization when the reaction was stopped after 3 h and heated at 70 °C. Alternatively, **XIVb** might evolve toward lactone **XVII** and then toward **8**. This requires prolonged reaction time (>12 h) and a higher temperature (80 °C) (Figure 2).

**Figure 3:**
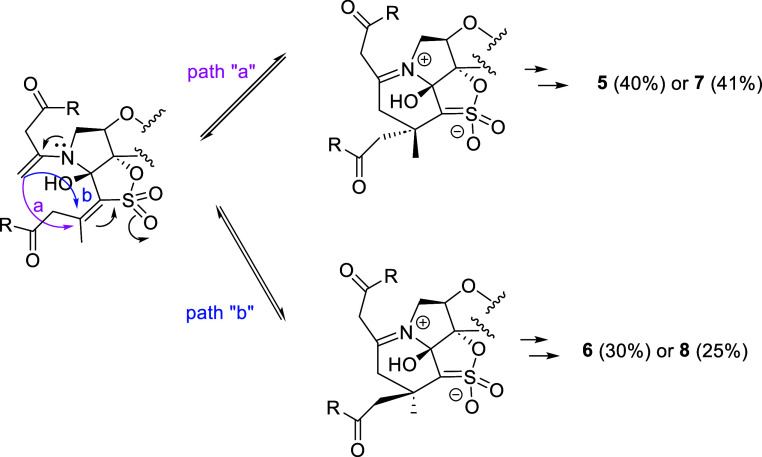
Proposed paths “a” (attack from the bottom face of the furanose) and “b” (attack from the top face of the furanose).

It should be mentioned that compounds **5** and **7** were obtained with a higher yield than that of **6** and **8** (Table 1). This observation supports the reasonable hypothesis that path “a”, which, as shown in Figure 3, implies the intramolecular attack of the enamine to the activated double bond (C=C–SO_2_) through the bottom face of the sugar, is more favorable than path “b”, in which the attack takes place through the top face.

### Studies to Support the Proposed Mechanism

**Scheme 3:**
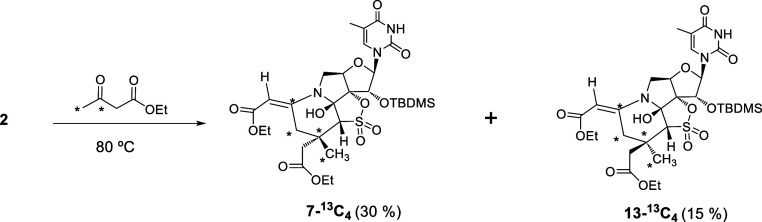
Conversion of **2** into **7-**^**13**^**C**_**4**_ and **13-**^**13**^**C**_**4**_

Finally, to obtain evidence of the participation of the acetyl fragment of the reagent in the construction of the new extra six-membered ring present in compounds **5**–**12**, we carried out the reaction of **2** with the C^13^-enriched ethyl acetoacetate (*CH_3_*COCH_2_COOCH_2_CH_3_) (Scheme 3).

When **2** was treated with the above-mentioned C^13^-enriched reagent in acetonitrile at 70 °C for 3 h, a mixture of **7-**^**13**^**C**_**4**_ and **13-**^**13**^**C**_**4**_ was isolated in 30% and 15% yield, respectively (Scheme 3).

Next, ^1^H NMR studies were performed to determine the position of the carbon labels. In this respect, it is known^31−36^ that ^13^C is a stable isotope with magnetic properties (NMR active) that splits hydrogen atoms to which it is attached and to which it is adjacent. Consequently, the resonance lines of the affected proton signals split into well-established patterns when *J*-coupled to a ^13^C nucleus.

**Figure 4:**
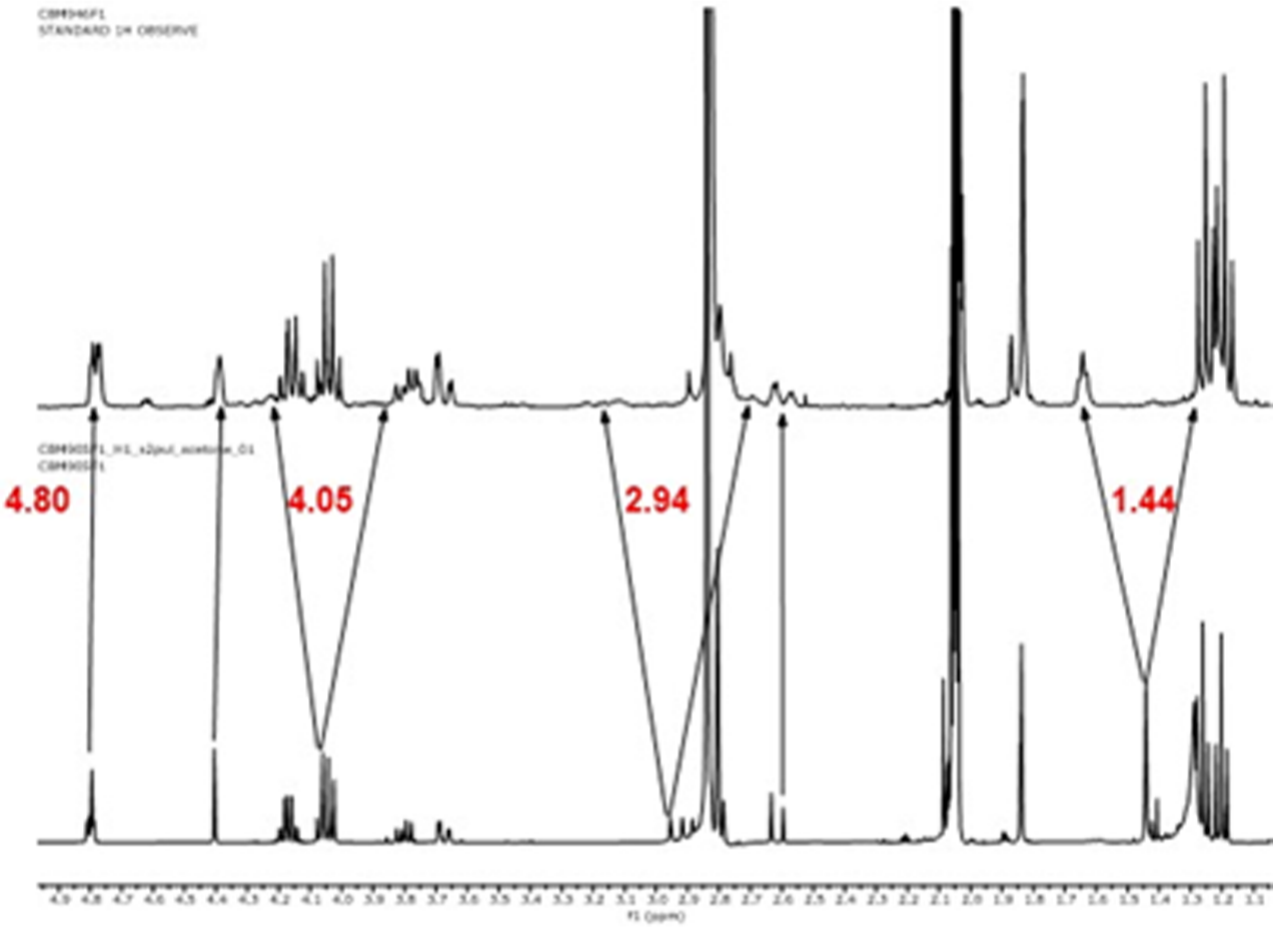
(Up) Portion of the ^1^H NMR spectrum of **7-**^**13**^**C**_**4**_; the (Down) same portion of the ^1^H NMR spectrum of **7**.

**Figure 5:**
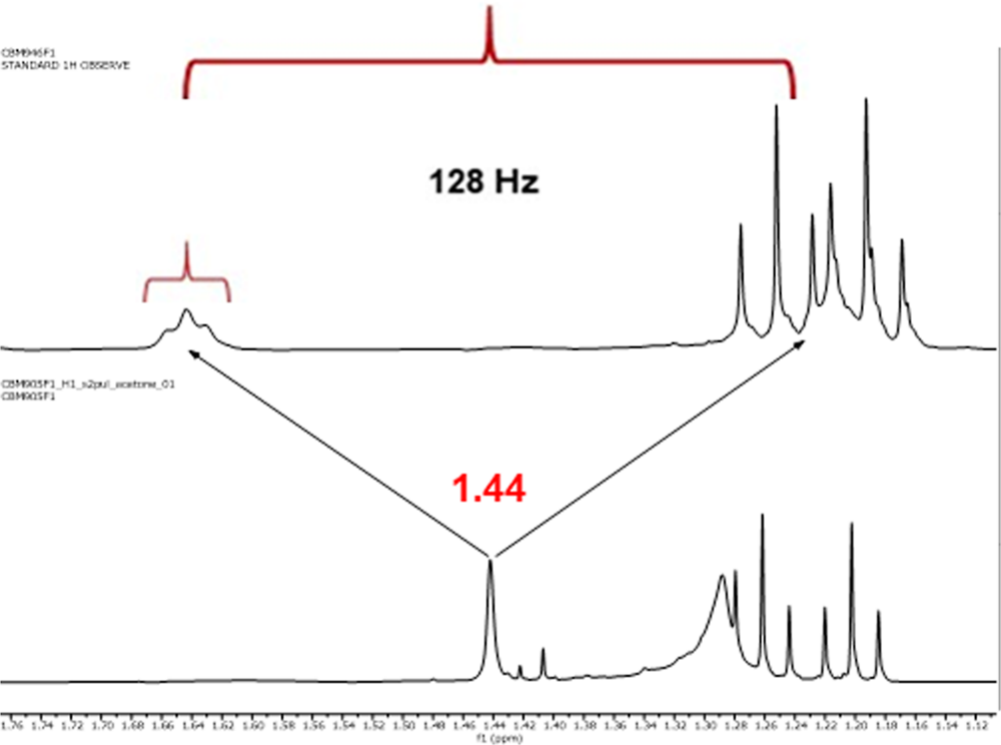
Expansion of the region corresponding to CH_3_ (δ = 1.44 ppm in **7**).

Inspection of the ^1^H NMR spectra of **7-**^**13**^**C**_**4**_ clearly showed the ^13^C isotope effects on the ^1^H chemical shifts of the CH_3_ and CH_2_-cycle protons. These protons resonate at δ 1.44 ppm (CH_3_) and δ 2.94, 4.05 ppm (CH_2_-cycle), respectively, in the unlabeled compound **7** (Figure 4, down). Interestingly, in the labeled **7-**^**13**^**C**_**4**_ (Figure 4, up), isotopic enrichment of ^13^C was detected by *J*-splitting of these resonances. The coupling constant ^1^*J*_CH_ of 128 Hz can be easily observed in each case. However, the coupling constants ^2^*J*_CH_ and ^3^*J*_CH_, due to labeled carbons being nondirectly linked to these protons, are more difficult to determine, although they are also observed. For example, the singlet corresponding to CH_3_ that appears at δ 1.44 ppm in **7** becomes a doublet of pseudo triplets in **7-**^**13**^**C**_**4**_ instead of a doublet, indicating that the adjacent carbons are also labeled (Figure 5). Also, the exocyclic vinylic proton (δ 4.80 ppm) is affected due to the existence of a coupling constant ^2^*J*_CH_ (Figure 4).

Similarly, in the case of **13-**^**13**^**C**_**4**_, the ^1^H NMR spectra also showed *J*-splitting of the CH_2_-cycle resonance (δ 3.29, 3.38 ppm) and CH_3_ resonance (δ 1.59 ppm). Also, the exocyclic vinylic proton is affected (δ = 4.84 ppm).

**Figure 6:**
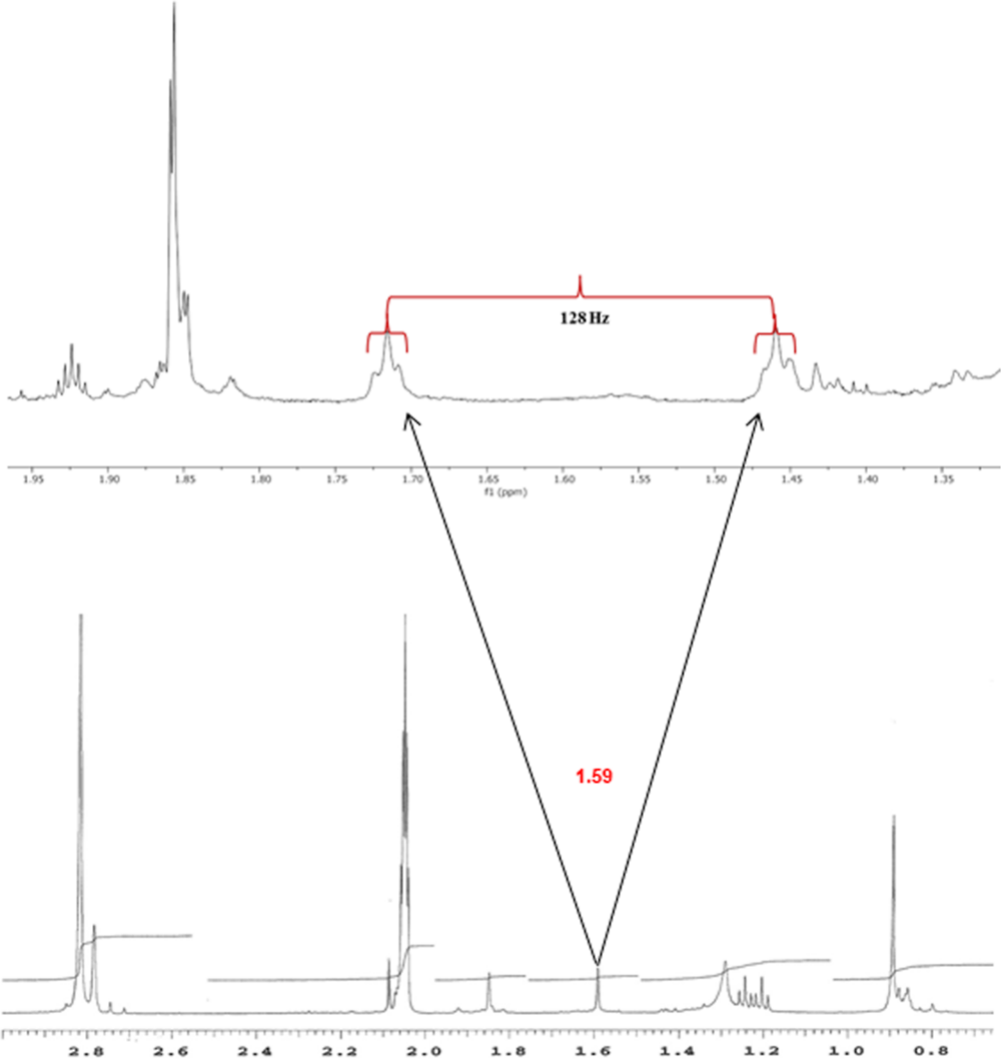
Expansion of the region corresponding to CH_3_ (δ = 1.59 ppm in **13**).

As shown in Figure 6, for the signal corresponding to CH_3_ (δ 1.59 ppm), the coupling constant ^1^*J*_CH_ of 128 Hz can be easily observed. Coupling constants ^2^*J*_CH_ and ^3^*J*_CH_, due to labeled carbons being nondirectly linked to these protons, are also observed, but they are more difficult to determine.

To summarize this part, by comparing 1D-^1^H NMR experiments performed with and without ^13^C decoupling during acquisition, we were able to trace the environment of CH_3_ and CO enriched with ^13^C. Those experiments provide strong support for the participation of the CH_3_CO fragment of the reagent in the formation of the new six-membered ring fused to the precursor ring system present in **2** (see Scheme 3).

### Structural Assignments

The structures of the new compounds **5**–**12** were assigned by NMR (^1^H and ^13^C) spectroscopy using mono- and 2-dimensional techniques (COSY, gHMBC,^37,38^ gHSQC,^38^ NOESY, and ROESY experiments) together with MS studies.

### Compound **5**

**Figure 7:**
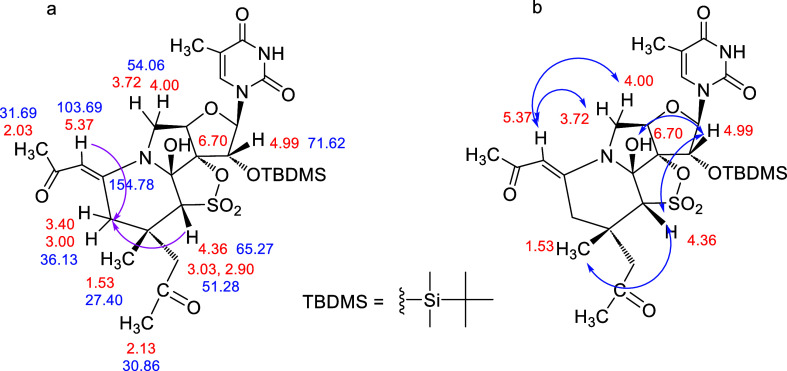
(a) Most relevant gHSQC correlations and key bond connectivities identified by gHMBC in **5** (pink arrows); (b) ROESY correlations in **5** (blue arrows).

The ^1^H NMR spectrum and ^1^H COSY experiment showed two characteristic AB spin systems, with signals at δ 3.40 and 3.00 ppm (*J*_AB_ = 17.4 Hz) and δ 3.03 and 2.90 ppm (*J*_AB_ = 18.9 Hz), that were indicative of the presence of two isolated methylene groups (see Supporting Information). Correlations of those signals with carbon atoms at δ 36.13 and 51.28 ppm, respectively, were observed in a gHMBC experiment. Furthermore, the presence of carbon signals at δ 154.78 (quaternary) and 103.69 ppm (CH at δ 5.37 ppm) indicated the presence of one double bond. The ^1^H NMR spectrum also showed three new singlet peaks at δ 1.53 (3H), 2.03 (3H, overlaps with the deuterated solvent), and 2.13 ppm (3H, overlaps with the deuterated solvent). The gHMBC spectrum provided several key correlations that supported the structure of **5**. In particular (Figure 7a), long-range correlations were observed between protons at δ 4.36 (proton adjacent to SO_2_ and CH–SO_2_) and 5.37 ppm (H-vinylic) and the carbon at δ 36.13 ppm (CH_2_-cycle). A ROESY experiment (Figure 7b) showed correlations between the H-2′ proton of the sugar (δ 4.99 ppm) and protons at δ 4.36 (CH–SO_2_) and 6.70 ppm (OH at the α position of the pyrrolidine ring), indicating that all of these protons are oriented at the top face of the furanose ring (above the plane of the furanose ring). Moreover, the new CH_3_ signal at δ 1.53 ppm shows correlation to CH–SO_2_ (δ 4.36 ppm), concluding that this methyl moiety is also oriented toward the top face of the furanose ring. Finally, the signal at δ 5.37 ppm (olefinic proton) correlates to the sugar protons H-5′_a_ and H-5′_b_ (δ 3.72 and 4.00 ppm), confirming the stereochemistry of the double bond proposed for **5**.

### Compound **6**

**Figure 8:**
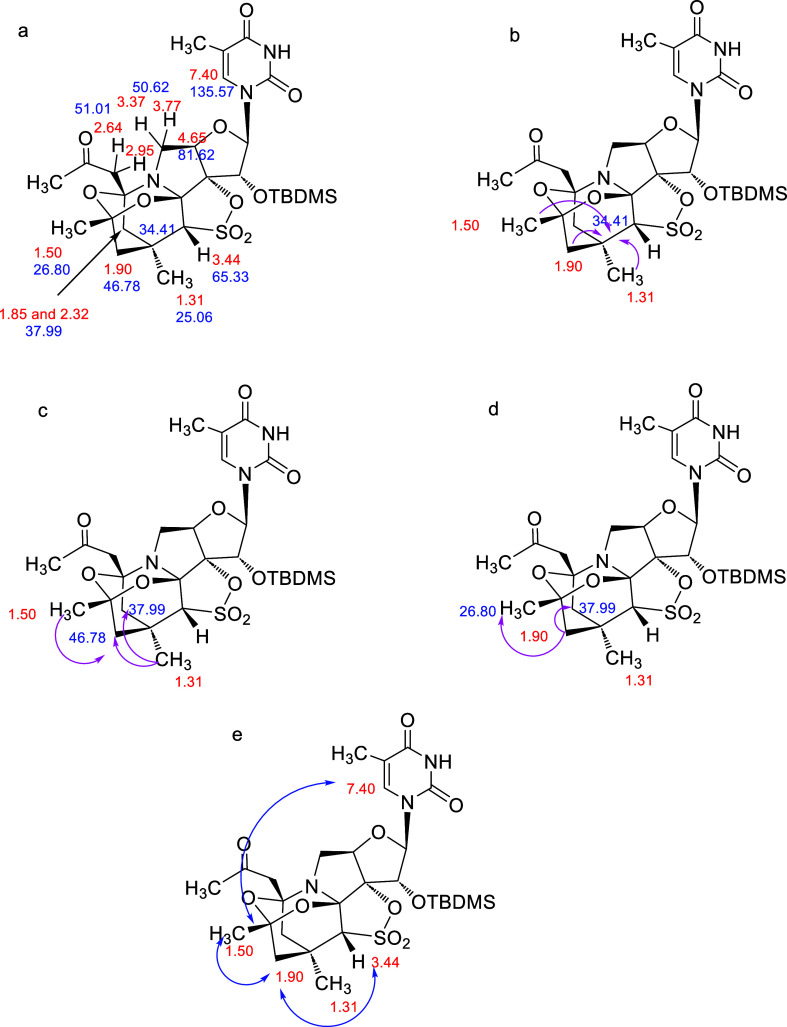
(a–d) Most relevant gHSQC correlations and key bond connectivities identified by gHMBC in **6** (pink arrows); (e) ROESY correlations in **6** (blue arrows).

The most intriguing feature of the ^1^H NMR spectrum of **6** is that the signal ascribed to the OH group at the α position of the pyrrolidine ring was absent (see Supporting Information). In the gHSQC experiment (Figure 8a), the presence of a new characteristic AB system, with protons at δ 1.85 and 2.32 ppm, was observed. These protons correlate with the same carbon atom at δ 37.99 ppm. Moreover, three new signal peaks at δ 1.31, δ 1.50, and δ 1.90 ppm that correlate with carbons at δ 25.06 (CH_3_), 26.80 (CH_3_), and 46.78 (CH_2_-cycle) ppm, respectively, were shown. Each of these three new signals (δ 1.31, 1.50, and 1.90 ppm) showed, in the HMBC experiment, a long-range correlation with the signal of the quaternary carbon at δ 34.41 ppm (Figure 8b). Moreover, the CH_3_ at δ 1.31 ppm showed long-range correlations with the CH_2_ carbons at δ 37.99 and δ 46.78 ppm, while the CH_3_ at δ 1.50 ppm showed a long-range correlation with the CH_2_ carbon at δ 46.78 ppm (Figure 8c). Finally, the CH_2_ at δ 1.90 ppm correlates to the CH_2_ carbon at δ 37.99 ppm and to the CH_3_ carbon at δ 26.80 ppm (Figure 8d). All of these correlations are possible only if the proposed extra cycle is present in the structure. A ROESY experiment (Figure 8e) showed a correlation between the protons at δ 1.90 ppm (CH_2_ cycle) and the protons at δ 3.44 (CH–SO_2_) and 1.50 ppm (new CH_3_). Moreover, the signal at δ 7.40 ppm (H-6 of the nucleobase) correlates with the new CH_3_ (δ 1.50 ppm), confirming that all these protons were at the same top face of the furanose ring.

### Compound **7**

**Figure 9:**
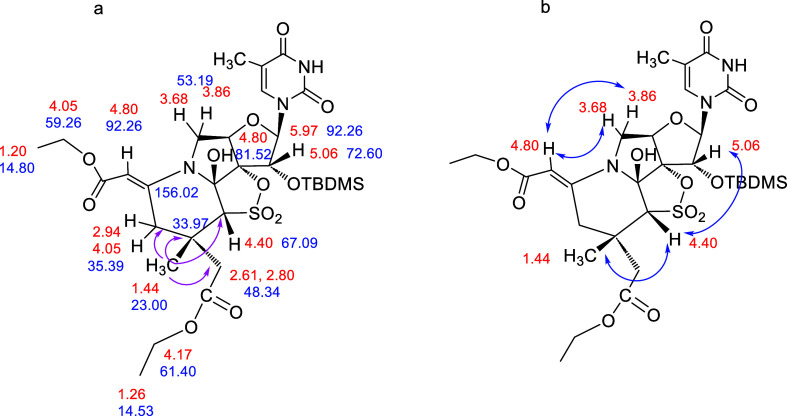
(a) Most relevant gHSQC correlations and key bond connectivities identified by gHMBC in **7** (pink arrows); (b) ROESY correlations in **7** (blue arrows).

The gHSQC spectrum of **7** (Figure 9a) shows similarities with those of **5**, like the presence of two new characteristic AB systems, protons at δ 2.94 and 4.05 ppm, that correlate with the same carbon atom at 35.39 ppm and protons at δ 2.61 and 2.80 ppm that correlate with the same carbon atom at 48.34 ppm. In addition, one exocyclic double bond with carbon atoms at 156.02 (quaternary) and 92.26 ppm (CH), together with a new singlet at δ 1.44 ppm (3H, CH_3_), was also observed. The gHMBC experiment (Figure 9a) showed long-range correlations between the new CH_3_ (δ 1.44 ppm) and the carbons at δ 33.97 (quaternary), 35.39 (CH_2_-cycle), 48.34 (CH_2_CO), and 67.09 ppm (CH–SO_2_) that confirmed the proposed structure. The signal of the new methyl moiety at δ 1.44 ppm correlates, in a ROESY experiment (Figure 9b), with the signal at δ 4.40 ppm (CH–SO_2_), which in turn correlates with the H-2′ of the sugar (δ 5.06 ppm), indicating that all of these protons are on the same top face of the furanose ring. As it was observed for **5**, the signal at δ 4.80 ppm (olefinic proton) correlates to the sugar protons H-5′_a_ and H-5′_b_ (δ 3.68 and 3.86 ppm), confirming the stereochemistry of the double bond proposed for **7**.

### Compound **8**

**Figure 10:**
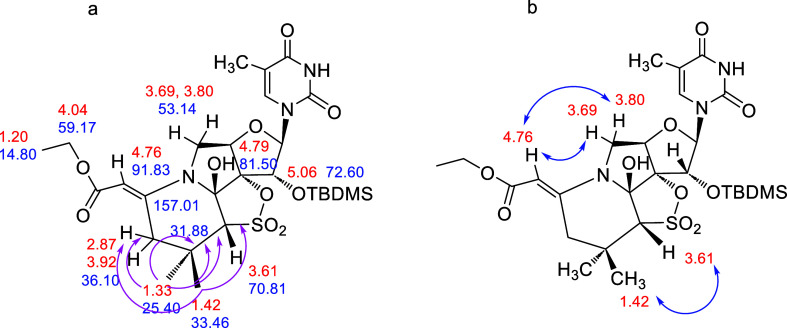
(a) Most relevant gHSQC correlations and key bond connectivities identified by gHMBC in **8** (pink arrows); (b) ROESY correlations in **8** (blue arrows).

Two significant differences in the ^1^H NMR spectra of **8** with respect to those of **7** were found. The first one is that only one ethoxy fragment, instead of two, was observed. The second is that two new singlets at δ 1.33 and δ 1.42 ppm (3H each, CH_3_), instead of one, were observed (see Supporting Information). The gHMBC spectrum (Figure 10a) showed long-range correlations between the two new CH_3_ (δ 1.33 and 1.42 ppm) and the carbons at δ 31.88 (quaternary), 36.10 (CH_2_-cycle), and 70.81 ppm (CH–SO_2_) that confirmed the proposed structure. The new CH_3_ at δ 1.42 ppm correlates in a ROESY experiment with the signal at δ 3.61 ppm (CH–SO_2_) (Figure 10b). Based on our antecedents, we make the reasonable hypothesis that the stereochemistry of the CH–SO_2_ carbon is preserved. Thus, the attached proton CH–SO_2_ should be on the top face of the furanose ring, and consequently, the new methyl group (δ 1.42 ppm) should also be on this side of the molecule. On the other hand, the signal at δ 4.76 ppm (olefinic proton) correlates to the sugar protons H-5′_a_ and H-5′_b_ (δ 3.69 and 3.80 ppm), confirming the stereochemistry of the double bond proposed for **8**.

Finally, the electrospray ionization (ESI)-mass spectrum of **8** exhibited a [M + H]^+^ peak at *m*/*z* 628.38 Da, while **7** showed a peak at *m*/*z* 700.5 Da. This difference in mass (71.6 Da) is compatible with the loss in **8** of an ethoxy carbonyl fragment (CO_2_CH_2_CH_3_: 72 Da).

Spectral analysis of **9** and **10** (see Supporting Information) demonstrated that their structures, shown in Table 1, are similar, respectively, to those of the previously described compounds, **3** and **4** (Figure 1).

### Compound **11**

**Figure 11:**
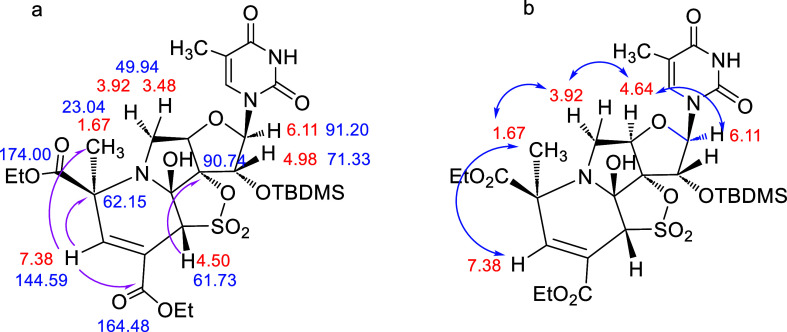
(a) Most relevant gHSQC correlations and key bond connectivities identified by gHMBC in **11** (pink arrows); (b) ROESY correlations in **11** (blue arrows).

The observed ^1^H NMR spectra of **11** showed two significant differences with respect to those of expected **10** (see Table 1 for structural comparison). The first one is the absence of the characteristic AB system present in **10**, and the second is the presence of two singlets at δ 7.38 ppm (vinylic proton) and δ 4.50 ppm (CH–SO_2_). In the gHMBC spectrum (Figure 11a), long-range correlations were observed between the vinylic proton (δ 7.38 ppm) and the carbons at δ 23.04 (CH_3_), 62.15 (quaternary), and 164.48 ppm (CO). Moreover, the CH–SO_2_ proton (δ 4.50 ppm) showed a long-range correlation with the carbon at δ 90.74 ppm (quaternary), corroborating the structure proposed for **11**.

### Compound **12**

**Figure 12:**
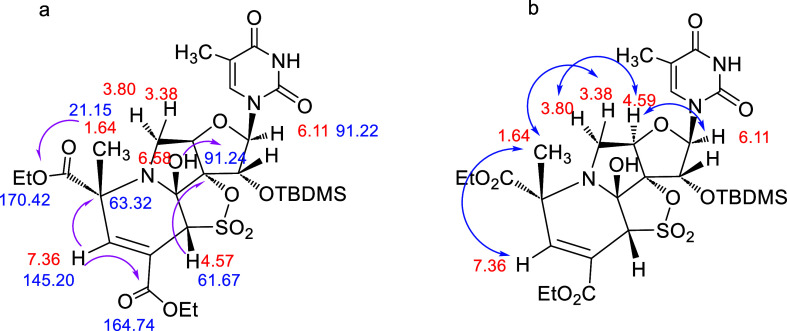
(a) Most relevant gHSQC correlations and key bond connectivities identified by gHMBC in **12** (pink arrows); (b) ROESY correlations in **12** (blue arrows).

The ^1^H NMR spectra of **12** showed three almost identical singlets (δ 7.36, 4.57, and 1.64 ppm), respectively, to those observed for **11** (Figure 12a). Moreover, the bond connectivities identified by gHMBC are also very similar (Figure 12a). Finally, ROESY experiments confirm that compounds **11** and **12** differ only in the stereochemistry of the new CH_3_. Thus, in **11** (Figure 11b), the signal of the new methyl moiety (δ 1.67 ppm) correlates with the signal at δ 3.92 ppm, corresponding to the H-5′b proton of the furanose ring, which also correlates with the H-4′proton at δ 4.64 ppm, indicating that all of these protons are on the same bottom face of the furanose ring. However, in **12** (Figure 12b), the new methyl moiety that appears at δ 1.64 ppm correlates with the H-5′a proton at δ 3.38 ppm. On the other hand, the vicinal H-5′b proton (δ 3.80 ppm) showed a correlation with the H-4′ proton at δ 4.59 ppm that is in the bottom face of the molecule. Thus, these spectral data were indicative that the H-5′a proton (δ 3.38 ppm) and the new CH_3_ (δ 1.64 ppm) are both on the same top face of the furanose ring.

## Conclusions

In summary, we have developed an efficient and completely regioselective and stereoselective procedure to generate molecular complexity from the hydroxy tricyclic precursor **2**. Our results agree with previous studies of our group that suggest the great potential of **2** to achieve molecular complexity in an efficient way. The synthetic route, which involves an enamine-iminium mechanism, is able to generate unusual ring systems together with two new C–C bonds and several stereocenters with high selectivity in one single step. A plausible mechanism, consistent with a ^13^C-enriched-labeling experiment, has been proposed for these transformations.

In addition to the regio- and stereoselectivity, efficiency, and rapid generation of molecular complexity, the present methodology is particularly attractive because it may provide access to novel polycyclic systems without the use of catalysts or aggressive conditions.

The new polycyclic compounds described here can be good scaffolds for diversification. In fact, some of them can be useful substrates for subsequent modification and derivatization. This can be regarded as an improvement over our previous work,^10^ in which only access to compounds (i.e., **3** and **4**) with inert alkyl groups was achieved.

We consider it of interest to determine if the results reported in this paper can be extended to other transformations with broader applicability. With this aim, simple glycosides that retain structural motifs similar to those present in **2** should be taken into consideration. On the one hand, the α hydroxypyrrolidine ring provides both the secondary amino group and the hydroxyl moiety that are crucial for reaction with carbonyl compounds and efficient formation of iminium–enamine intermediates. On the other hand, an electrophilic partner, in our case, vinyl sulfone, might facilitate the intramolecular cyclization. These two structural motifs seem to be essential, with the ultimate goal of generating structural complexity in a single step.

## Experimental Section

### General Chemistry Procedures

Commercial reagents and solvents were used as received from the suppliers without further purification, unless otherwise stated. The acetonitrile used as the solvent was dried prior to use.

Analytical thin-layer chromatography (TLC) was performed on aluminum plates precoated with silica gel 60 (*F*_254_, 0.20 mm). Products were visualized using an ultraviolet lamp (254 and 365 nm) or by heating after treatment with a 5% solution of phosphomolybdic acid (PMA) or vanillin in ethanol.

The compounds were purified by preparative centrifugal circular TLC (CCTLC) (Kiesegel 60 PF254 gipshaltig, layer thickness of 1 mm, flow rate of 2–4 mL/min).

For HPLC analysis, a compact LC with a reverse-phase column ACE 5C18–300 (4.6 × 150 mm, 3.5 μm) equipped with a PDA (photodiode array) detector was used. Acetonitrile was used as mobile phase A, and water with 0.05% of TFA was used as mobile phase B with a flow rate of 1 mL·min^–1^. All retention times are quoted in minutes, and the gradients are specified for each compound in the experimental data.

For high-resolution mass spectrometry (HRMS) of compound **6**, an Accurate Mass QTOF (quadrupole time-of-flight) platform coupled with LC/MS and equipped with an electrospray interface working in positive-ion (ESI^+^) mode was used. For HRMS of compounds **5**, **7**–**13**, **7-**^**13**^**C**_**4**_, and **13-**^**13**^**C**_**4**_, a LC/QTOF platform coupled with UHPLC and equipped with an electrospray interface working in the positive-ion (ESI^+^) mode was used.

NMR spectra (^1^H and ^13^C {^1^H} NMR) were recorded on 400 (400 and 100 MHz) and 500 MHz systems. The 500 MHz system was equipped with a 5 mm HCN cold probe (500 and 125 MHz) spectrometer using (CD_3_)_2_CO as solvent. They were performed using standard pulse sequences. Chemical shift (δ) values are reported in parts per million (ppm) relative to (CD_3_)_2_CO (δ = 2.05) in ^1^H and (δ = 29.84) ^13^C NMR. Coupling constant values (*J* values) are reported in hertz (Hz), and multiplicities of signals are indicated by the following symbols: s (singlet), d (doublet), t (triplet), q (quadruplet), m (multiplet), and bs (broad singlet). Two-dimensional spectra (COSY, gHSQC, gHMBC, NOESY, and ROESY) were obtained to identify the structure.

Final compounds were lyophilized using a Telstar 6–80 system.

### Note of Nomenclature

The names of the polycyclic nucleosides are given according to the IUPAC recommendations for polycyclic compounds (extension of the Von Baeyer system) (see Supporting Information).^39^ However, for easy comparison, the assignments of the signals in the NMR spectra follow standard carbohydrate/nucleoside numbering (i.e., the furanose skeleton numbered 1′–5′) with the thymine moiety having the highest priority. The spirosultone skeleton was numbered 1″-4″ starting from the oxygen (see Supporting Information).

### Nucleosides **5** and **6**

To a solution of nucleoside **2**^10^ (0.020 g, 0.04 mmol) in dry acetonitrile (1 mL) was added acetylacetone (0.098 mL, 0.8 mmol). The reaction mixture was stirred at 80 °C for 24 h and then evaporated to dryness. The residue was purified on a CCTLC purification system on a normal phase using dichloromethane/methanol (40:1) as eluent.

The fastest moving fractions afforded **5** (0.011 g, 40%) as a white foam.^1^H NMR [500 MHz, (CD_3_)_2_CO] δ 0.02 and 0.19 (6H, 2s, 2CH_3_)_,_ 0.89 (9H, s, *t*-Bu), 1.53 (3H, s, CH_3_), 1.85 (3H, d, *J* = 1.3 Hz, CH_3_-5), 2.03 (3H, s, CH_3_CO), 2.13 (3H, s, CH_3_CO), 2.90 (1H, d, *J* = 18.9 Hz, CH_2_aCO), 3.03 (1H, d, *J* = 18.9 Hz, CH_2_bCO), 3.00 (1H, d, *J* = 17.4 Hz, CH_2_-cyclo), 3.40 (1H, d, *J* = 17.4 Hz, CH_2_-cyclo), 3.72 (1H, dd, *J*_5′a,5′b_ = 12.0 Hz, *J*_4′,5′a_ = 4.2 Hz, H-5′a), 4.00 (1H, dd, *J*_5′a,5′b_ = 12.0 Hz, *J*_4′,5′b_ = 7.6 Hz, H-5′b), 4.36 (1H, s, CH–SO_2_), 4.75 (1H, dd, *J*_4′,5′a_ = 4.1 Hz, *J*_4′,5′b_ = 7.5 Hz, H-4′), 4.99 (1H, d, *J*_1′,2′_ = 6.7 Hz, H-2′), 5.37 (s, 1H, C=CH), 6.07 (1H, d, *J*_1′,2′_ = 6.7 Hz, H-1′), 6.70 (1H, s, OH-4″), 7.54 (1H, s, H-6), 10.26 (1H, br s, NH-3). ^13^C NMR [125 MHz, (CD_3_)_2_CO]: δ −4.96 (CH_3_), −4.54 (CH_3_), 12.56 (CH_3_-5), 18.44 *C*(CH_3_)_3_, 25.97 C(*CH*_3_)_3_, 27.40 (CH_3_), 30.86 (*CH*_3_CO), 31.69 (*CH*_*3*_CO), 32.78 (C), 36.13 (CH_2_-cyclo), 51.28 (CH_2_CO), 54.06 (C-5′), 65.27 (CH–SO_2_), 71.62 (C-2′), 81.09 (C-4′), 91.53 (C-4″), 91.73 (C-1′), 96.01 (C-3′), 103.69 (CH=), 112.46 (C-5), 136.22 (C-6), 151.40 (C-2), 154.78 (C=), 163.74 (C-4), 195.46 (CO), 206.19 (CO). HPLC [gradient:H_2_O/MeCN, 10–100 in 5 min]: 4.64 min. HRMS (ESI^+^) *m/z:* calcd for C_28_H_41_N_3_O_10_SSi: 639.2282; found, 662.2269.

The slowest moving fractions afforded **6** (0.008 g, 30%) as a white foam. ^1^H NMR [500 MHz, (CD_3_)_2_CO]: δ −0.03 and 0.14 (6H, 2 s, 2CH_3_), 0.88 (9H, s, *t*-Bu), 1.31 (3H, s, CH_3_), 1.50 (3H, s, CH_3_), 1.85 (1H, dd, CH_2_-cyclo, *J* = 13.5 Hz, 1.1 Hz), 1.86 (3H, s, CH_3_-5), 1.90 (2H, s, CH_2_), 2.21 (3H, s, CH_3_CO), 2.32 (1H, dd, CH_2_-cyclo, *J* = 13.5 Hz, 1.7 Hz), 2.64 (1H, d, CH_2a_CO, *J* = 14.1 Hz), 2.95 (1H, d, CH_2_bCO, *J* = 14.1 Hz), 3.37 (1H, dd, *J*_5′a,5′b_ = 9.0 Hz, *J*_4′,5′a_ = 5.3 Hz, H-5′a), 3.44 (1H, s, CH–SO_2_), 3.77 (1H, dd, *J*_5′a,5′b_ = 9.0 Hz, *J*_4′,5′b_ = 7.1 Hz, H-5′b), 4.59 (1H, d, *J*_1′,2′_ = 6.7 Hz, H-2′), 4.65 (1H, d, *J*_4′,5′b_ = 7.1 Hz, H-4′), 6.09 (1H, d, *J*_1′,2′_ = 6.7 Hz, H-1′), 7.40 (1H, d, *J* = 1.3 Hz, H-6), 10.21 (1H, br s, NH-3). ^13^C NMR δ [125 MHz, (CD_3_)_2_CO]: δ −4.85 (CH_3_), −4.70 (CH_3_), 12.56 (CH_3_-5), 18.36 *C*(CH_3_)_3_, 25.06 (CH_3_), 25.94 C(*CH*_3_)_3_, 26.80 (CH_3_), 32.08 (*CH*_3_CO), 34.41 (C), 37.99 (CH_2_-cyclo), 46.78 (CH_2_), 50.62 (C-5′), 51.01 (CH_2_CO), 65.33 (CH–SO_2_), 69.27 (C-2′), 81.62 (C-4′), 85.82 (C), 91.34 (C-1′), 91.99 (C-4″), 95.06 (C-3′), 102.51 (C), 112.62 (C-5), 135.57 (C-6), 151.28 (C-2), 163.73 (C-4), 204.16 (CO). HPLC [gradient: H_2_O/MeCN, 10–100 in 5 min]: 5.22 min. HRMS (ESI^+^) *m/z:* calcd for C_28_H_41_N_3_O_10_SSi: 639.2282; found, 639.2292.

### Nucleosides **7** and **8**

To a solution of nucleoside **2**^10^ (0.020 g, 0.04 mmol) in dry acetonitrile (1 mL), ethyl acetoacetate (0.095 mL, 0.8 mmol) was added. The reaction mixture was stirred at 80 °C for 24 h and then evaporated to dryness. The residue was purified on a CCTLC purification system on a normal phase using hexane/ethyl acetate (3:1) as eluent.

The fastest moving fractions afforded **7** (0.012 g, 41%) as a white foam. ^1^H NMR [400 MHz, (CD_3_)_2_CO]: δ 0.06 and 0.18 (6H, 2s, 2CH_3_), 0.89 (9H, s, *t*-But), 1.20 (3H, t, *J* = 7.1 Hz, *CH*_*3*_CH_2_O), 1.26 (3H, t, *J* = 7.1 Hz, *CH*_*3*_CH_2_O), 1.44 (3H, s, CH_3_), 1.84 (3H, d, *J* = 1.3 Hz, CH_3_-5), 2.61 (1H, d, *J* = 14.9 Hz, CH_2_aCO), 2.80 (1H, d, *J* = 14.9 Hz, CH_2_bCO), 2.94 (1H, d, *J* = 14.9 Hz, CH_2_-cyclo), 3.68 (1H, dd, *J*_5′a,5′b_ = 12.1 Hz, *J*_4′,5′a_ = 2.4 Hz, H-5′a), 3.86 (1H, dd, *J*_5′a,5′b_ = 12.1 Hz, *J*_4′,5′b_ = 6.7 Hz, H-5′b), 4.05 (3H, q, *J* = 7.06 Hz, OCH_2_ and d, *J* = 14.9 Hz, CH_2_-cyclo), 4.17 (2H, q, *J* = 7.06 Hz, OCH_2_), 4.40 (1H, s, CH–SO_2_), 4.80 (2H, dd, *J*_4′,5′a_ = 2.4 Hz, *J*_4′,5′b_ = 6.7 Hz, H-4′ and s, CH=), 5.06 (1H, d, *J*_1′,2′_ = 6.4 Hz, H-2′), 5.97 (1H, d, *J*_1′,2′_ = 6.4 Hz, H-1′), 6.79 (1H, s, OH-4″), 7.61 (1H, s, H-6), 10.31 (1H, br s, NH-3). ^13^C NMR [100 MHz, (CD_3_)_2_CO]: δ −4.96 (CH_3_), −4.45 (CH_3_), 12.43 (CH_3_-5), 14.53 (CH_3_), 14.80 (CH_3_), 18.56 *C*(CH_3_)_3_, 23.00 (CH_3_), 25.93 C(*CH*_3_)_3_, 33.97 (C), 35.39 (CH_2_-cycle), 48.34 (*CH*_*2*_CO), 53.19 (C-5′), 59.26 (OCH_2_), 61.40 (OCH_2_), 67.09 (CH–SO_2_), 72.60 (C-2′), 81.52 (C-4′), 92.26 (CH= and C-1′), 94.24 (C-4″), 94.85 (C-3′), 112.42 (C-5), 136.93 (C-6), 151.55 (C-2), 156.02 (C=CH), 163.55 (C-4), 168.03 (CO), 171.12 (CO). HPLC [gradient: H_2_O/MeCN, 10–100 in 5 min]: 5.47 min. MS (ESI^+^) *m*/*z*: [M + H]^+^ 700.5. HRMS (ESI^+^) *m/z:* calcd for C_30_H_45_N_3_O_12_SSi: 699.2493; found, 699.2477.

The slowest moving fractions afforded **8** (0.007 g, 25%) as a white foam. ^1^H NMR [400 MHz, (CD_3_)_2_CO]: δ 0.04 and 0.15 (6H, 2s, 2CH_3_), 0.88 (9H, s, *t*-But), 1.20 (3H, t, *CH*_*3*_CH_2_O, *J* = 7.1 Hz), 1.33 (3H, s, CH_3_), 1.42 (3H, s, CH_3_), 1.84 (3H, s, CH_3_-5), 2.87 (1H, d, *J* = 15.3 Hz, CH_2_-cyclo), 3.61 (1H, s, CH–SO_2_), 3.69 (1H, dd, *J*_5′a,5′b_ = 12.0 Hz, *J*_4′,5′a_ = 2.7 Hz, H-5′a), 3.80 (1H, dd, *J*_5′a,5′b_ = 12.0 Hz, *J*_4′,5′b_ = 6.9 Hz, H-5′b), 3.92 (1H, d, *J* = 15.3 Hz, CH_2_-cyclo), 4.04 (2H, q, OCH_2_), 4.76 (1H, s, CH=), 4.79 (1H, dd, *J*_4′,5′a_ = 2.6 Hz, *J*_4′,5′b_ = 6.7 Hz, H-4′), 5.06 (1H, d, *J*_1′,2′_ = 6.4 Hz, H-2′), 5.99 (1H, d, *J*_1′,2′_ = 6.4 Hz, H-1′), 6.62 (1H, s, OH-4″), 7.67 (1H, s, H-6). ^13^C NMR [100 MHz, (CD_3_)_2_CO]: δ −4.95 (CH_3_), −4.55 (CH_3_), 12.42 (CH_3_-5), 14.80 (CH_3_), 18.52 *C*(CH_3_)_3_, 25.40 (CH_3_), 25.90 C(*CH*_3_)_3_, 31.88 *C*(CH_3_)_2_, 33.46 (CH_3_), 36.10 (CH_2_-cycle), 53.14 (C-5′), 59.17 (OCH_2_), 70.81 (CH–SO_2_), 72.60 (C-2′), 81.50 (C-4′), 91.64 (C-1′), 91.83 (CH=), 94.21 (C-4″), 95.12 (C-3′), 112.31 (C-5), 136.83 (C-6), 152.12 (C-2), 157.01 (C=), 164.41 (C-4), 168.10 (CO). HPLC [gradient: H_2_O/MeCN, 10–100 in 5 min]: 5.49. MS (ESI^+^) *m*/*z*: [M + H]^+^ 628.38. HRMS (ESI^+^) *m/z:* calcd for C_27_H_41_N_3_O_10_SSi: 627.2282; found, 627.2272.

### Nucleosides **9** and **10**

To a solution of nucleoside **2**^10^ (0.020 g, 0.04 mmol) in dry acetonitrile (1 mL), ethyl pyruvate (0.094 mL, 0.8 mmol) was added. The reaction mixture was stirred at room temperature for 12 h and then evaporated to dryness. The residue was purified on a CCTLC purification system on a normal phase using hexane/ethyl acetate (3:1) as eluent.

The fastest moving fractions afforded **9** (0.012 g, 44%) as a white foam.^1^H NMR [500 MHz, (CD_3_)_2_CO]: δ 0.09 (3H, s, CH_3_), 0.19 (3H, s, CH_3_), 0.91 (9H, s, *t*-But), 1.29 (3H, t, *J* = 7.1 Hz, *CH*_*3*_CH_2_O), 1.31 (3H, t, *J* = 7.1 Hz, *CH*_*3*_CH_2_O), 1.74 (3H, s, CH_3_), 1.81 (3H, d, *J* = 1.3 Hz, CH_3_-5), 3.52 (1H, dd, *J*_5′a,5′b_ = 12.0 Hz, *J*_4′,5′a_ = 5.9 Hz, H-5′a), 3.90 (1H, s, CH–SO_2_), 4.20 (2H, q, *J* = 7.1 Hz, OCH_2_), 4.27 (2H, q, *J* = 7.1 Hz, OCH_2_), 4.35 (1H, dd, *J*_5′a,5′b_ = 12.0 Hz, *J*_4′,5′b_ = 1.2 Hz, H-5′b), 4.69 (1H, dd, *J*_4′,5′a_ = 5.9 Hz, *J*_4′,5′b_ = 1.2 Hz, H-4′), 5.04 (1H, d, *J*_1′,2′_ = 5.6 Hz, H-2′), 5.99 (1H, d, *J*_1′,2′_ = 5.6 Hz, H-1′), 6.36 (1H, d, *J* = 0.9 Hz, CH=), 6.47 (1H, s, OH-4″), 7.69 (1H, s, H-6), 10.27 (1H, br s, NH-3). ^13^C NMR [125 MHz, (CD_3_)_2_CO]: δ −4.98 (CH_3_), −4.64 (CH_3_), 12.45 (CH_3_-5), 14.05 (*CH*_*3*_CH_2_O), 14.41 (*CH*_*3*_CH_2_O), 18.59 *C*(CH_3_)_3_, 25.96 C(*CH*_3_)_3_, 27.59 (CH_3_), 44.27 (C), 54.38 (C-5′), 62.09 (OCH_2_), 62.73 (OCH_2_), 62.81 (CH–SO_2_), 72.48 (C-2′), 81.57 (C-4′), 92.12 (C-1′), 93.97 (C-3′), 94.42 (C-4″), 112.23 (C-5), 114.96 (CH=), 132.43 (C=), 136.90 (C-6), 151.49 (C-2), 163.84 (CO, C-4), 171.22 (CO). HPLC [gradient: H_2_O/MeCN, 40–100 in 5 min] tr: 4.62. HRMS (ESI^+^) *m/z:* calcd for C_28_H_41_N_3_O_12_SSi: 671.2180; found, 672.2172.

The slowest moving fractions afforded **10** (0.009 g, 33%) as a white foam. ^1^H NMR [500 MHz, (CD_3_)_2_CO]: δ 0.04 and 0.13 (6H, 2s, 2CH_3_), 0.89 (9H, s, *t*-But), 1.28 (3H, t, *J* = 7.4 Hz, *CH*_*3*_CH_2_O), 1.34 (3H, t, *J* = 7.5 Hz, *CH*_*3*_CH_2_O), 1.54 (3H, s, CH_3_), 1.92 (3H, s, CH_3_-5), 2.79 (1H, dd, *J*_5′a,5′b_ = 11.8 Hz, *J*_4′,5′a_ = 4.5 Hz, H-5′a), 2.83 (1H, overlaps with H_2_O, CH_2_a), 3.40 (d, 1H, CH_2_b, *J* = 19.8 Hz), 3.42 (1H, dd, *J*_5′a,5′b_ = 11.8 Hz, *J*_4′,5′b_ = 1.0 Hz, H-5′b), 4.18 (2H, q, OCH_2_), 4.31 (2H, q, OCH_2_), 4.51 (1H, d, *J*_4′,5′a_ = 4.5 Hz, *J*_4′,5′b_ = 1.0 Hz, H-4′), 4.91 (1H, d, *J*_1′,2′_ = 5.4 Hz, H-2′), 5.94 (1H, d, *J*_1′,2′_ = 5.4 Hz, H-1′), 6.11 (1H, s, OH-4″), 7.58 (1H, s, H-6), 10.19 (1H, br s, NH-3). ^13^C NMR [125 MHz, (CD_3_)_2_CO]: δ −4.71 (CH_3_), −4.68 (CH_3_), 12.70 (CH_3_-5), 13.97 (*CH*_*3*_CH_2_O), 14.38 (*CH*_*3*_CH_2_O), 18.62 *C*(CH_3_)_3_, 24.92 (CH_3_), 26.10 C(*CH*_3_)_3_, 31.12 (CH_2_-cycle), 49.71 (C-5′), 60.29 (C), 62.14 (OCH_2_), 63.31 (OCH_2_), 74.27 (C-2′), 81.67 (C-4′), 91.75 (C-1′), 92.31 (C-4″), 94.14 (C-3′), 111.70 (C-5), 136.69 (C-6), 137.19 (C=C), 137.96 (C=C–SO_2_), 151.47 (C-2), 163.73 (CO), 163.99 (C-4), 174.76 (CO). HPLC [gradient: H_2_O/MeCN, 40–100 in 5 min] tr: 4.16. HRMS (ESI^+^) *m/z:* calcd for C_28_H_41_N_3_O_12_SSi: 671.2180; found, 672.2167.

### Nucleosides **11** and **12**

To a solution of nucleoside **2**^10^ (0.020 g, 0.04 mmol) in dry acetonitrile (1 mL), ethyl pyruvate (0.094 mL, 0.8 mmol) was added. The reaction mixture was stirred at 80 °C for 24 h and then evaporated to dryness. The residue was purified on a CCTLC purification system on a normal phase using hexane/ethyl acetate (3:1) as eluent.

The fastest moving fractions afforded **11** (0.007 g, 25%) as a white foam. ^1^H NMR [500 MHz, (CD_3_)_2_CO]: δ 0.01 and 0.18 (6H, 2s, 2CH_3_), 0.90 (9H, s, *t*-But), 1.30 (6H, t, 2 *CH*_*3*_CH_2_O, *J* = 7.3 Hz), 1.67 (3H, s, CH_3_), 1.84 (3H, s, CH_3_-5), 3.48 (1H, dd, *J*_5′a,5′b_ = 9.7 Hz, *J*_4′,5′a_ = 5.1 Hz, H-5′a), 3.92 (1H, dd, *J*_5′a,5′b_ = 9.7 Hz, *J*_4′,5′b_ = 7.4 Hz, H-5′b), 4.28 (4H, q, 2 OCH_2_), 4.50 (1H, s, CH–SO_2_), 4.64 (1H, dd, *J*_4′,5′a_ = 5.1 Hz, *J*_4′,5′b_ = 7.4 Hz, H-4′), 4.98 (1H, d, J_1′,2′_ = 7.1 Hz, H-2′), 6.03 (1H, s, OH-4″), 6.11 (1H, d, *J*_1′,2′_ = 7.1 Hz, H-1′), 7.38 (1H, d, *J* = 0.7 Hz, CH=), 7.61 (1H, s, H-6), 10.25 (1H, br s, NH-3). ^13^C NMR [125 MHz, (CD_3_)_2_CO] δ −4.85 (CH_3_), −4.65 (CH_3_), 12.45 (CH_3_-5), 14.25 (*CH*_*3*_CH_2_O), 14.27 (*CH*_*3*_CH_2_), 18.45 *C*(CH_3_)_3_, 23.04 (CH_3_), 26.00 C(*CH*_3_)_3_, 49.94 (C-5′), 61.73 (CH–SO_2_), 62.15 (C), 62.55 (OCH_2_), 63.57 (OCH_2_), 71.33 (C-2′), 81.89 (C-4′), 90.74 (C-3′), 91.20 (C-1′), 96.66 (C-4″), 112.24 (C-5), 121.29 (C), 136.14 (C-6), 144.59 (C=CH), 151.43 (C-2), 163.84 (CO), 164.48 (CO, C-4), 174.00 (CO). HPLC [gradient:H_2_O/MeCN, 40–100 in 5 min] tr: 4.47. HRMS (ESI^+^) *m*/*z*: calcd for C_28_H_41_N_3_O_12_SSi: 671.2180; found, 672.2169.

The intermediate moving fractions afforded 0.010 g (37%) of a compound (white foam) whose ^1^H, ^13^C NMR, and mass spectrum correspond to those of **9**.

The slowest moving fractions afforded **12** (0.005 g, 18%) as a white foam. ^1^H NMR [500 MHz, (CD_3_)_2_CO]: δ −0.01 and 0.19 (6H, 2s, 2 CH_3_), 0.90 (9H, s, *t*-But), 1.24 (3H, t, *CH*_*3*_CH_2_O, *J* = 6.9 Hz), 1.30 (3H, t, *CH*_*3*_CH_2_O, *J* = 6.8 Hz), 1.64 (3H, s, CH_3_), 1.86 (3H, s, CH_3_-5), 3.38 (1H, dd, *J*_5′a,5′b_ = 9.2 Hz, *J*_4′,5′a_ = 5.8 Hz, H-5′a), 3.80 (1H, dd, *J*_5′a,5′b_ = 9.2 Hz, *J*_4′,5′b_ = 7.4 Hz, H-5′b), 4.17 (2H, m, OCH_2_), 4.27 (2H, m, OCH_2_), 4.57 (1H, s, CH–SO_2_), 4.59 (1H, dd, *J*_4′,5′a_ = 5.8 Hz, *J*_4′,5′b_ = 7.4 Hz, H-4′), 4.95 (1H, d, *J*_1′,2′_ = 7.4 Hz, H-2′), 6.11 (1H, d, *J*_1′,2′_ = 7.4 Hz, H-1′), 6.58 (1H, s, OH-4″), 7.36 (1H, s, CH=), 7.67 (1H, s, H-6), 10.22 (1H, br s, NH-3). ^13^C NMR [125 MHz, (CD_3_)_2_CO]: δ −4.76 (CH_3_), −4.52 (CH_3_), 12.45 (CH_3_-5), 14.25 (*CH*_*3*_CH_2_O), 14.31 (*CH*_*3*_CH_2_O), 18.43 *C*(CH_3_)_3_, 21.15 (CH_3_), 26.05 C(*CH*_3_)_3_, 50.06 (C-5′), 61.67 (CH–SO_2_), 62.41 (OCH_2_), 62.60 (OCH_2_), 63.32 (C), 71.18 (C-2′), 81.90 (C-4′), 91.22 (C-1′), 91.24 (C-3′), 96.87 (C-4″), 112.22 (C-5), 120.72 (C=), 136.34 (C-6), 145.20 (C=CH), 151.46 (C-2), 163.66 (C-4), 164.74 (CO), 170.42 (CO). HPLC [gradient: H_2_O/MeCN, 40–100 in 5 min] tr: 4.00. HRMS (ESI^+^) *m*/*z*: calcd for C_28_H_41_N_3_O_12_SSi 671.2180; found, 671.2166.

### Nucleoside **13**

To a solution of nucleoside **2**^10^ (0.020 g, 0.04 mmol) in dry acetonitrile (1 mL), ethyl acetoacetate (0.095 mL, 0.8 mmol) was added. The reaction mixture was stirred at 70 °C for 3 h and then evaporated to dryness. The residue was purified on a CCTLC purification system on a normal phase using dichloromethane/methanol (40:1) as eluent.

The fastest moving fractions afforded **13** (0.006 g, 20%) as a white foam. ^1^H NMR [500 MHz, (CD_3_)_2_CO]: δ 0.13 and 0.19 (6H, 2s, 2 CH_3_), 0.89 (9H, s, *t*-But), 1.20 (3H, t, *J* = 7.1 Hz, *CH*_*3*_CH_2_O), 1.24 (3H, t, *J* = 7.1 Hz, *CH*_3_CH_2_O), 1.59 (3H, s, CH_3_), 1.85 (3H, d, *J* = 1.3 Hz, CH_3_-5), 2.72, 2.84 (2H, CH_2a_CO and CH_2b_CO), 3.29 (m, CH_2_-cyclo), 3.38 (m, CH_2_-cyclo), 3.73 (1H, dd, *J*_5′a,5′b_ = 11.7 Hz, *J*_4′,5′a_ = 3.9 Hz, H-5′a), 3.94 (1H, dd, *J*_5′a,5′b_ = 11.7 Hz, *J*_4′,5′b_ = 7.3 Hz, H-5′b), 4.05 (2H, m, OCH_2_), 4.14 (2H, m, OCH_2_), 4.16 (1H, s, CH–SO_2_), 4.77 (1H, dd, *J*_4′,5′a_ = 3.9 Hz, *J*_4′,5′b_ = 7.3 Hz, H-4′), 4.84 (1H, s, CH=), 5.03 (1H, d, *J*_1′,2′_ = 6.6 Hz, H-2′), 6.06 (1H, d, *J*_1′,2′_ = 6.6 Hz, H-1′), 6.73 (1H, s, OH-4″), 7.59 (1H, s, H-6), 10.25 (1H, br s, NH-3). ^13^C NMR [125 MHz, (CD_3_)_2_CO]: δ −4.88 (CH_3_), −4.52 (CH_3_), 12.53 (CH_3_-5), 14.46 (*CH*_3_CH_2_O), 14.82 (*CH*_*3*_CH_2_O), 18.46 *C*(CH_3_)_3_, 25.99C(*CH*_3_)_3_, 27.63 (CH_3_), 33.61 (C), 34.90 (CH_2_-cycle), 42.64 (CH_2_), 53.75 (C-5′), 59.29 (OCH_2_), 61.02 (OCH_2_), 66.98 (CH–SO_2_), 71.76 (C-2′), 81.22 (C-4′), 91.86 (C-1′), 92.21 (C-3′), 94.85 (CH=), 96.03 (C-4″), 112.40 (C-5), 136.40 (C-6), 151.43 (C-2), 155.39 (=C), 163.72 (C-4), 167.64 (CO), 170.48 (CO). HPLC [gradient: H_2_O/MeCN, 10–100 in 5 min]: 5.54 min. MS (ESI^+^) *m*/*z*: [M + H]^+^ 700.5. HRMS (ESI^+^) *m/z:* calculated for C_30_H_45_N_3_O_12_SSi, 699.2493; found, 699.2480.

The slowest moving fractions afforded 0.009 g (34%) of a compound (white foam) whose ^1^H, ^13^C NMR, and mass spectrum correspond to those of **7**.

#### ^13^C-Enriched Nucleosides **7-**^**13**^**C**_**4**_ and **13-**^**13**^**C**_**4**_

To a solution of nucleoside **2**^10^ (0.020 g, 0.04 mmol) in dry acetonitrile (1 mL), C^13^-enriched 3,4-^13^C_2_ ethyl acetoacetate (0.095 mL, 0.8 mmol) was added. The reaction mixture was heated at 70 °C for 3 h. The residue was purified on a CCTLC purification system on a normal phase using dichloromethane/methanol (40:1) as eluent.

The fastest moving fractions afforded **7-**^**13**^**C**_**4**_ (0.008 g, 30%) as a white foam. HPLC [gradient:H_2_O/MeCN, 10–100 in 5 min]: 5.51 min. HRMS (ESI^+^) *m*/*z*: calcd for C_26_[C^13^]_4_H_45_N_3_O_12_SSi, 703.2627; found, 703.2610.

The slowest moving fractions afforded **13-**^**13**^**C**_**4**_ (0.004 g, 15%) as a white foam. HPLC [gradient: H_2_O/MeCN, 10–100 in 5 min]: 5.51. HRMS (ESI^+^) *m*/*z*: calcd for C_26_[C^13^]_4_H_45_N_3_O_12_SSi, 703.2627; found, 726.2615.

## Data Availability

The data underlying this study are available in the published article and its Supporting Information.

## Supporting Information Available

Nomenclature of all the compounds; atom numbering of all the compounds; mechanistic considerations for the decarboxylation step; HPLC–ESI-MS studies; structural assignments of **9**, **10**, and **13**; 2-dimensional NMR procedures; and selected copies of NMR spectra (PDF)

## Abbreviations Used

COSY: correlated spectroscopy
ESI: electrospray ionization
gHMBC: gradient heteronuclear multiple bond coherence
gHSQC: gradient heteronuclear single quantum correlation
HRMS: high-resolution mass spectrometry
NOESY: nuclear overhauser effect spectroscopy
ROESY: rotating frame overhauser enhancement spectroscopy
MS: mass spectrometry
CCTLC: centrifugal circular thin-layer chromatography
