# Supplementary material for: Skeletal Transformations Observed in the Reaction of a Tricyclic Thymine Nucleoside with Dicarbonyl Compounds
Source: ACS Omega. 2024 Aug 12;9(34):36259–72. doi: 10.1021/acsomega.4c02553 (PMC11359620; doi:10.1021/acsomega.4c02553)

# SKELETAL TRANSFORMATIONS OBSERVED IN THE REACTION OF A TRICYCLIC THYMINE NUCLEOSIDE WITH DICARBONYL COMPOUNDS

*María-Cruz Bonache*[a] *Elisa G.-Doyagüez,*[b] *Raúl Benito-Arenas,*[c] *M. Angeles Bonache,*[a]

*María-Luisa Jimeno*[b] *and Ana San-Félix**[a]

[a]Instituto de Química Médica (CSIC), Juan de la Cierva 3, 28006 Madrid.
[b]Centro de Química Orgánica "Lora-Tamayo" (CSIC), Juan de la Cierva 3, 28006 Madrid
[c]Instituto de Química Orgánica General (CSIC), Juan de la Cierva 3,28006 Madrid

anarosa@iqm.csic.es

**Table of contents**

*Index* S1

*Nomenclature of all the compounds* S2

*Atom numbering of all the compounds* S4

*Mechanistic proposal for decarboxylation* S5

*HPLC-ESI-MS studies* S6

*Structural assignments of **9**, **10** and **13*** S9

*2-Dimensional NMR procedures* S12

*Selected copies of NMR spectra* S13

*Nomenclature of all the compounds*

- (1R, 5R, 10R, 12R, 13R, 14S)-13-(*tert*-butyldimethylsilyloxy)-3,3-dioxo-14-hydroxy-5-methyl-5-methylcarbonylmethylene-7-[1-(*E*) (methylcarbonyl)methylidene]-12-(thymin-1'-yl)-8-aza-2, 11-dioxa-3-thiatetracyclo[8.3.$1^{4,8}$.$0^{1,10}$.$0^{1,14}$]tetradecane (**5**).
- (1R, 5S, 10R, 12R, 13R, 14S, 16S)-13-(*tert*-butyldimethylsilyloxy)-3,3-dioxo-5,16-dimethyl-7-methylcarbonylmethylene-12-(thymin-1'-yl)-8-aza-2, 11, 15, 17-tetraoxa-3-thiahexacyclo[8.3.$3^{5,7}$.$1^{4,8}$.$1^{14,16}$.$0^{1,10}$]octadecane (**6**).
- (1R, 5S, 10R, 12R, 13R, 14S)-13-(*tert*-butyldimethylsilyloxy)-3,3-dioxo-14-hydroxy-5-methyl-5-ethoxycarbonylmethylene-7-[1-(*E*) (ethoxycarbonyl)methylidene]-12-(thymin-1'-yl)-8-aza-2, 11-dioxa-3-thiatetracyclo[8.3.$1^{4,8}$.$0^{1,10}$.$0^{1,14}$]tetradecane (**7**).
- (1R, 5R, 10R, 12R, 13R, 14S)-13-(*tert*-butyldimethylsilyloxy)-3,3-dioxo-14-hydroxy-5,5-dimethyl -7-[1-(*E*) (ethoxycarbonyl)methylidene]-12-(thymin-1'-yl)-8-aza-2, 11-dioxa-3-thiatetracyclo[8.3.$1^{4,8}$.$0^{1,10}$.$0^{1,14}$]tetradecane (**8**).
- (1R, 5S, 10R, 12R, 13R, 14S)-13-(*tert*-butyldimethylsilyloxy)-3,3-dioxo-14-hydroxy-5-ethoxycarbonyl-5-methyl-7-ethoxycarbonyl-12-(thymin-1'-yl)-8-aza-2, 11-dioxa-3-thiatetracyclo[8.3.$1^{4,8}$.$0^{1,10}$.$0^{1,14}$]tetradec-6-ene (**9**).
- (1R, 7R, 10R, 12R, 13R, 14S)-13-(*tert*-butyldimethylsilyloxy)-3,3-dioxo-14-hydroxy-7-ethoxycarbonyl-7-methyl-12-(thymin-1'-yl)-8-aza-2, 11-dioxa-3-thiatetracyclo[8.3.$1^{4,8}$.$0^{1,10}$.$0^{1,14}$]tetradec-4-ene (**10**).
- (1R, 7S, 10R, 12R, 13R, 14S)-13-(*tert*-butyldimethylsilyloxy)-3,3-dioxo-14-hydroxy-7-ethoxycarbonyl-7-methyl-12-(thymin-1'-yl)-8-aza-2, 11-dioxa-3-thiatetracyclo[8.3. $1^{4,8}$.$0^{1,10}$.$0^{1,14}$]tetradec-5-ene (**11**).

- (1R, 7R, 10R ,12R, 13R, 14S)-13-(*tert*-butyldimethylsilyloxy)-3,3-dioxo-14-hydroxy-7-ethoxycarbonyl-7-methyl-12-(thymin-1'-yl)-8-aza-2, 11-dioxa-3-thiatetracyclo[8.3.$1^{4,8}$.$0^{1,10}$.$0^{1,14}$]tetradec-5-ene (**12**).
- (1R, 5R, 10R, 12R, 13R, 14S)-13-(*tert*-butyldimethylsilyloxy)-3,3-dioxo-14-hydroxy-5-methyl-5-ethoxycarbonylmethylene-7-[1-(*E*) (ethoxycarbonyl)methylidene]-12-(thymin-1'-yl)-8-aza-2, 11-dioxa-3-thiatetracyclo[8.3.$1^{4,8}$.$0^{1,10}$.$0^{1,14}$]tetradecane (**13**).

*Atom numbering of all the compounds*

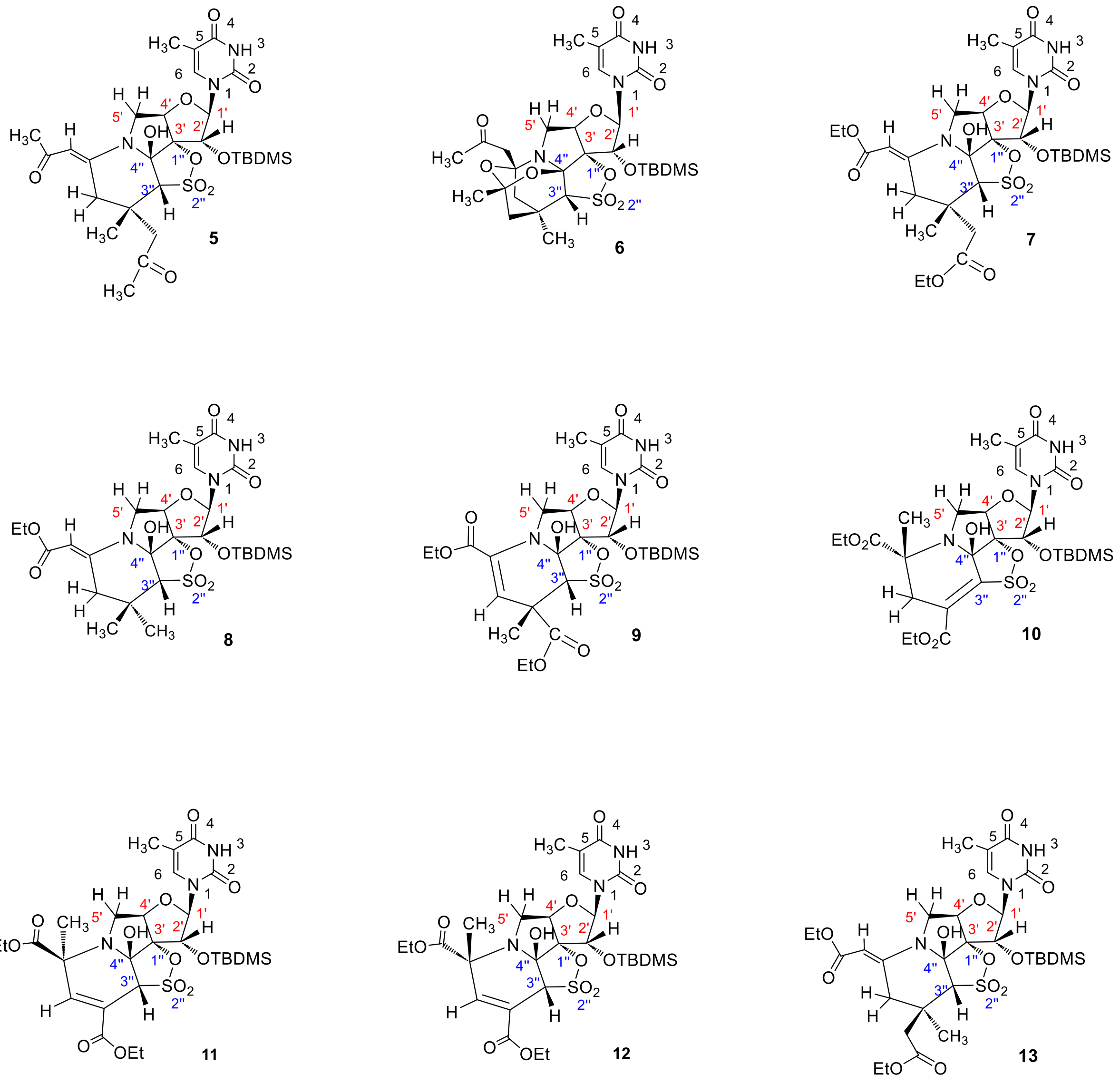


**FIGURE S1.** Atom numbering of all the compounds

*Mechanistic proposal for decarboxylation*

As was shown in Scheme 2 of the main manuscript, the reaction between **2** and ethyl acetoacetate follows a series of steps leading to intermediate **XVI** and then to the decarboxylated compound **8.** A possible pathway to explain the transformation of **XVI** in **8** is shown in Scheme S1.

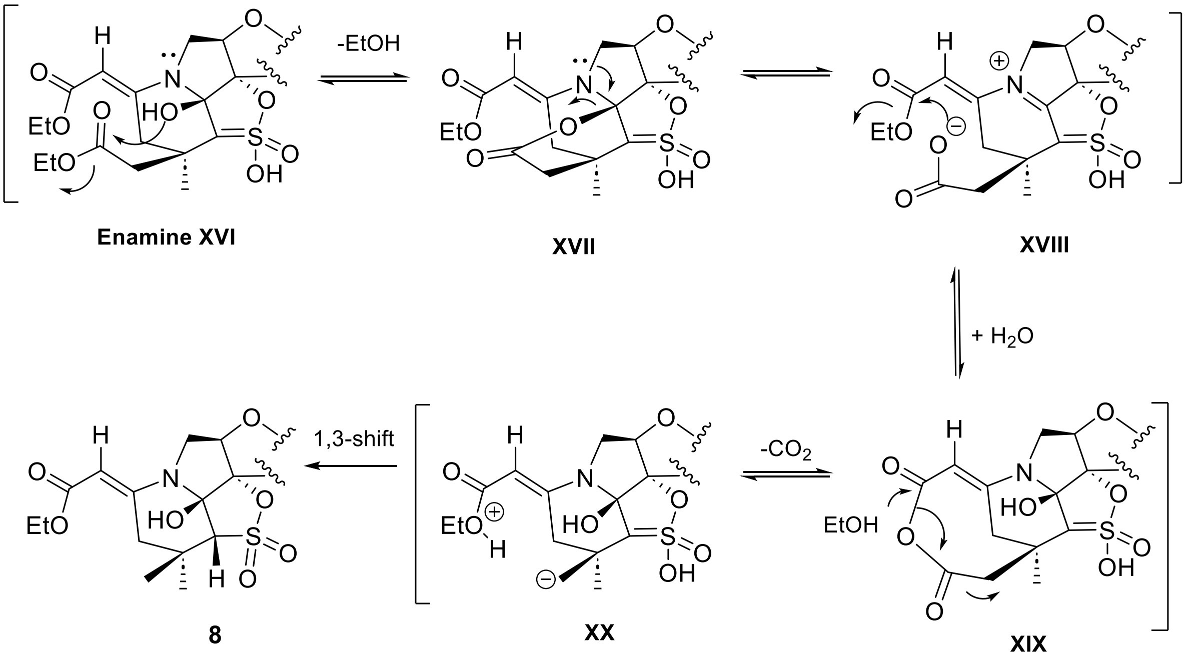


**Scheme S1.** Proposed mechanism for decarboxylation

Attack of the OH at the α-position of the pyrrolidine ring to the nearby ethyl ester would give lactone **XVII** through elimination of EtOH. Ring opening, with the assistance of the nitrogen of the pyrrolidine ring, should give intermediate **XVIII**. An equilibrium iminium-lactone, similar to that proposed in our mechanism, has been demonstrated in several natural zoanthamine products giving strong support to this step.[1] Next, the attack of water to the iminium ion present in **XVIII** and the intramolecular attack of the carboxylate anion to the ethyl ester might afford the anhydride **XIX**, which might experiment subsequent ethanol attack, followed by $CO_2$ extrusion, to generate intermediate **XX.** Finally, protonation of carbanion **XX** and 1,3-shift might afford compound **8**.

(1) Behenna, D. C.; Stockdill, J. L.; Stoltz, B. M. The Biology and Chemistry of the Zoanthamine Alkaloids. *Angew. Chem., Int. Ed.* **2008**, *47*, 2365–2386.

*HPLC-ESI-MS studies*

The reaction of **2** with ethyl acetoacetate (see Scheme 2 of the main manuscript) using acetonitrile:water (15:95) as gradient, was followed by HPLC-ESI-MS to support the proposed mechanism.

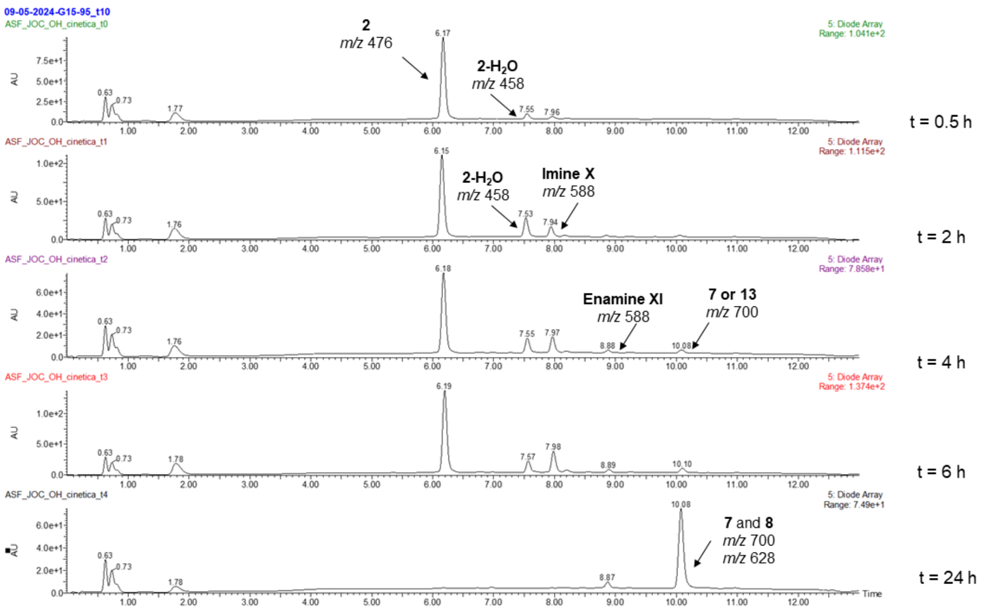


**FIGURE S2.** Evolution of the reaction of **2** in the presence of ethyl acetoacetate monitored by HPLC-ESI-MS at different times (0.5 h-24 h) on a C18 reverse-phase column.

As shown in Figure S2, after 2 h the signal at *m/z* 476, corresponding to the starting material (**2**), was still present. There were also two minor peaks at *m/z* 458 (rt: 7.55 min) and *m/z* 588 (rt: 7.97 min.), which can be assigned to a dehydrated form of the starting material and to the imine intermediate **X**, respectively (Figure S3). After 4 h, a new signal with the same mass than intermediate **X**, but different retention time (*m/z* 588, rt: 8.86 min.) appeared in the chemical noise (Figure S4). We hypothesized that this peak might belong to the enamine **XI**. Moreover, a signal compatible with the final compounds **7** or **13** (*m/z* 700, rt: 10.09 min.) also appeared at this time. After 6 h of reaction the signal of the imine

intermediate **X** increased. After 24 h, the signal of the starting material disappeared and only the signal at rt: 10.09 min was recognizable. This signal contained two very close peaks at *m/z* 700 (rt: 10.09 min) and *m/z* 628 (rt: 9.82 min), corresponding to the final compounds **7** and **8**, respectively (Figures S5 and S6). Other gradients: (50:95), (70:95) and (30:95) were investigated but these two peaks could not be separated.

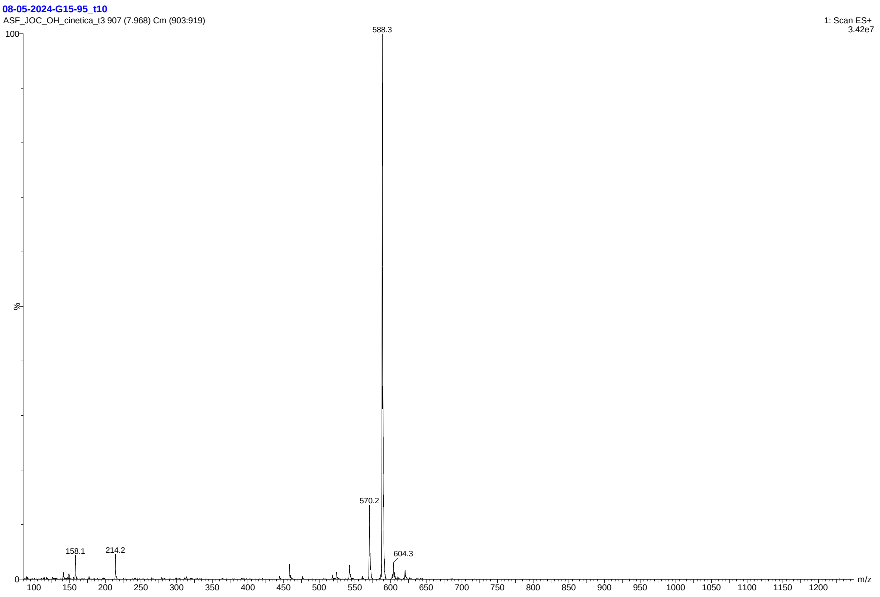


**FIGURE S3.** Mass spectra of the peak that appears at rt: 7.968 min (*m/z* 588), compatible with the imine **X**, showed after 2 h of reaction

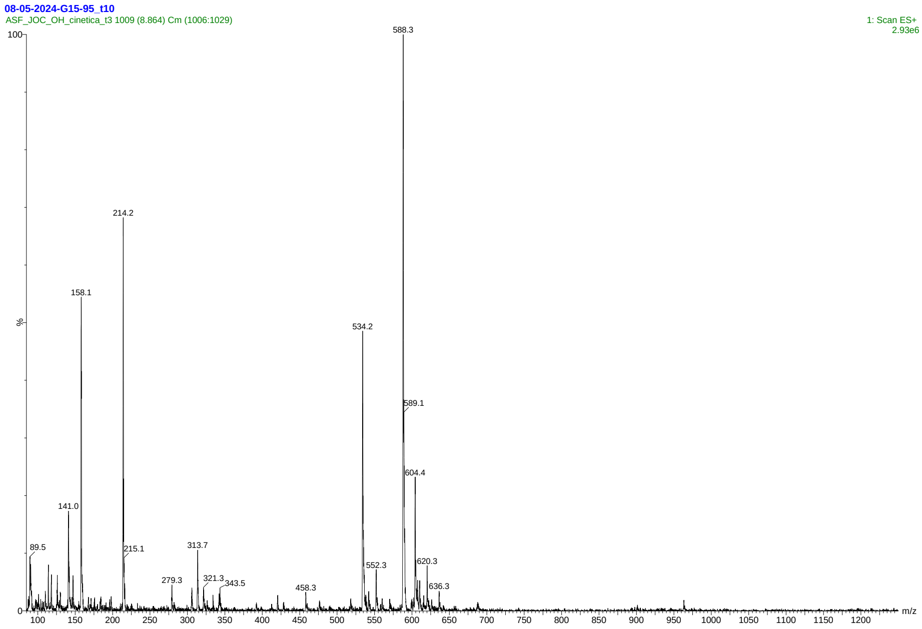


**FIGURE S4**. Mass spectra of the peak that appears at rt: 8.864 min (*m/z* 588), compatible with enamine **XI**, showed after 4 h of reaction

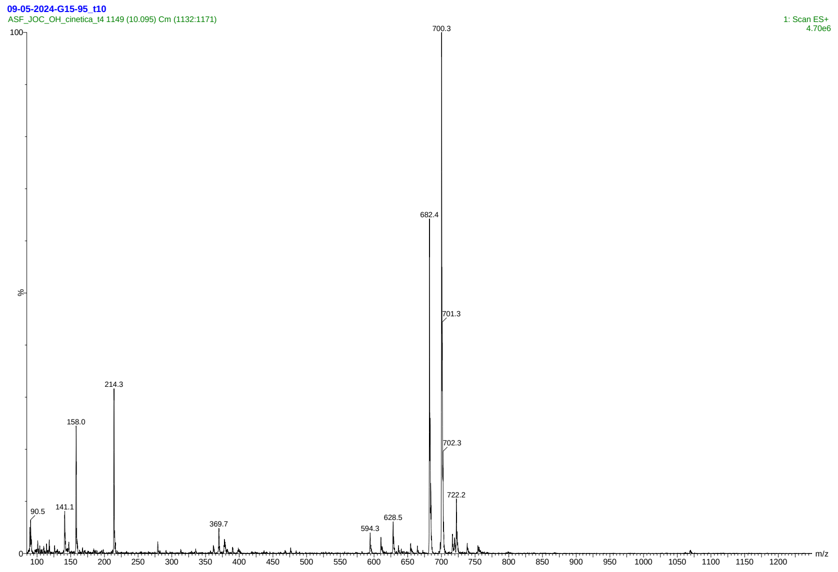


**FIGURE S5**. Mass spectra of the peak that appears at rt: 10.095 min (*m/z* 700), compatible with compound **7**, showed after 24 h of reaction

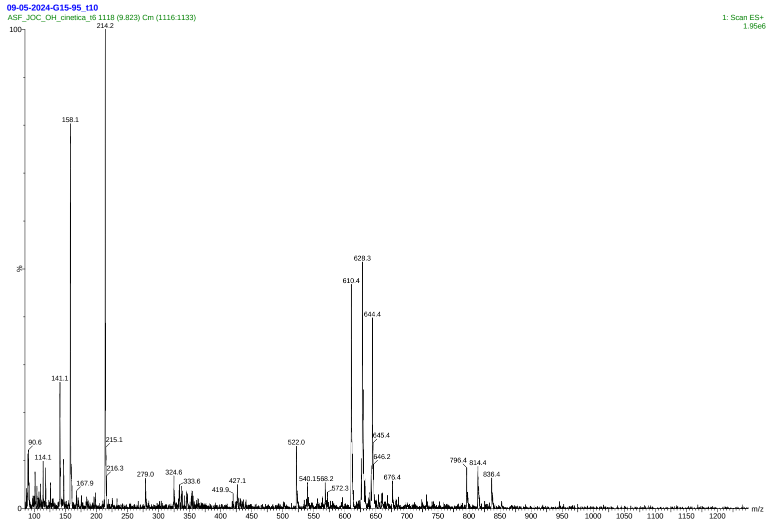


**FIGURE S6**. Mass spectra of the peak that appears at rt: 9.82 min (*m/z* 628), compatible with compound **8**, showed after 24 h of reaction

*Structural assignments of **9**, **10** and **13***

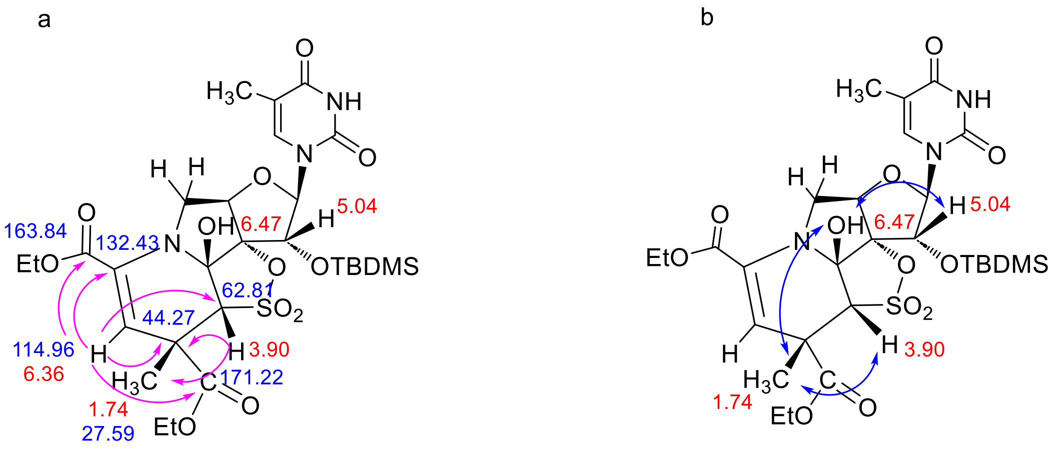


**FIGURE S7.** (a) Most relevant gHSQC correlations and key bond connectivities identified by gHMBC in **9** (pink arrows); (b) ROESY correlations in **9** (blue arrows).

The $^{1}H$ NMR spectrum of **9** (Figure S7) showed the disappearance of the characteristic AB system at ca. $\delta$ 3.60 ppm present in the starting compound (**2**) and the presence of two ethoxy fragments. Moreover, one double bond with carbon atoms at $\delta$ 132.43 ppm (quaternary) and $\delta$ 114.96 (CH), together with two new singlet peaks at $\delta$ 1.74 ppm (3H) and $\delta$ 6.36 (1H) ppm, were also observed. In the gHMBC experiment (Figure S7a) compound **9** showed long–range correlations between the vinylic proton ($\delta$ 6.36 ppm) and the two CO carbons at $\delta$ 171.22 and 163.84 ppm. The vinylic proton also showed long–range correlations with the quaternary carbons at $\delta$ 132.43 (double bond) and at $\delta$ 44.27 ppm (quaternary). Moreover, the $CH\text{-}SO_2$ proton ($\delta$ 3.90 ppm) showed correlations with the quaternary carbon at $\delta$ 44.27 ppm and the methyl carbon at $\delta$ 27.59 ppm. A ROESY experiment (Figure S7b) showed that the signal of the new $CH_3$ ($\delta$ 1.74 ppm) has a correlation with the signals at $\delta$ 3.90 ($H\text{-}SO_2$) and 6.47 ppm (OH at the α position of the pyrrolidine ring). In turn, this OH has a correlation with the H-2' of the sugar ($\delta$ 5.04 ppm) indicating that all of these protons were at the same upper side of the furanose ring.

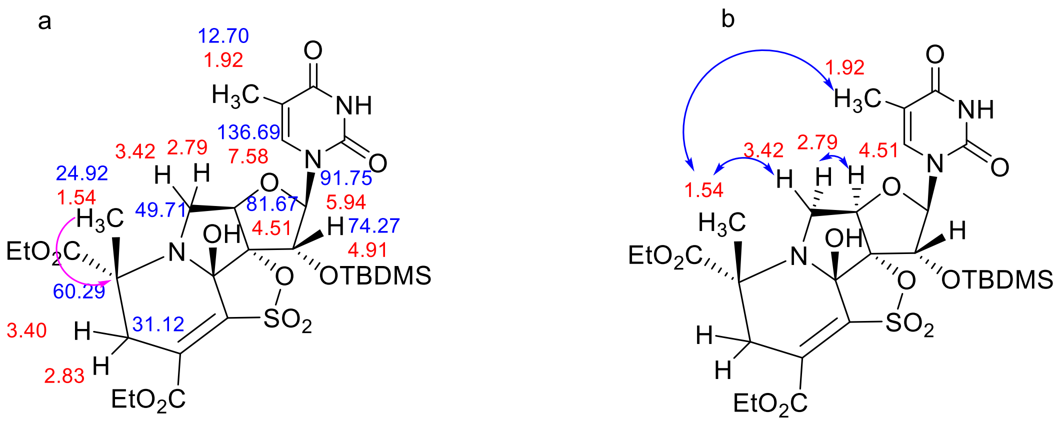


**FIGURE S8.** (a) Most relevant gHSQC correlations and key bond connectivities identified by gHMBC in **10** (pink arrows); (b) ROESY correlations in **10** (blue arrows).

The $^{1}H$ NMR spectrum of **10** showed the absence of the singlet corresponding to the CH-$SO_2$ proton and the presence of a characteristic AB system with two protons at $\delta$ 2.83 and 3.40 ppm that in the gHSQC experiment correlates with the same carbon atom at $\delta$ 31.12 ppm (Figure S8a). Moreover, apart from the two new ethoxy fragments, a new singlet peak at $\delta$ 1.54 ppm ($CH_3$), that correlates in gHMBC with a quaternary carbon at $\delta$ 60.29 ppm was observed. A ROESY experiment (Figure S8b) showed that the new $CH_3$ ($\delta$ 1.54 ppm) correlates with the signal at $\delta$ 3.42 ppm (H-5'b proton of the furanose ring) and very slightly with the signal at $\delta$ 1.92 ppm ($CH_3$-5) suggesting that all of these protons are oriented towards the same upper side of the furanose ring.

On the other hand, a correlation between the H-4' ($\delta$ 4.51 ppm) and the H-5'a ($\delta$ 2.79 ppm) and H-5'b ($\delta$ 3.42 ppm) protons of the furanose ring was observed, being the correlation with H-5'a much more intense than with H-5'b. This is a conclusive proof that the H-5'a ($\delta$ 2.79 ppm) and the H-4' protons, are oriented towards the same lower side of the furanose ring. Consequently, the vicinal H-5'b ($\delta$ 3.42 ppm) and the new $CH_3$ at $\delta$ 1.54 ppm,

with whom this proton correlates, must be oriented towards the same upper side of the furanose ring.

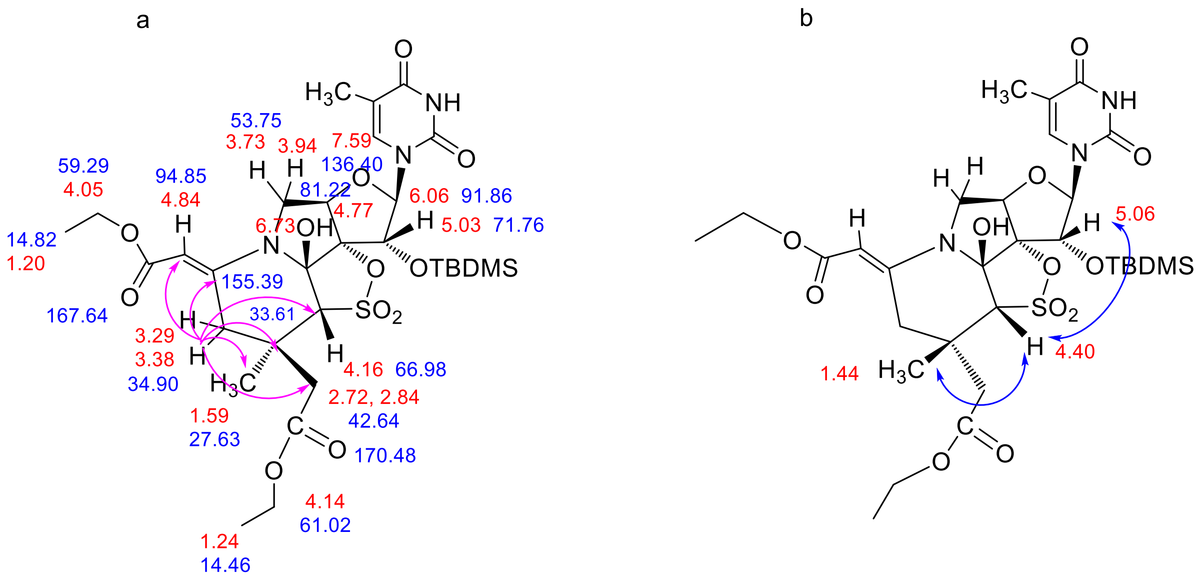


**FIGURE S9.** (a) Most relevant gHSQC and gHMBC correlations in **13**. (b) ROESY correlations in **7** for comparison.

The gHSQC spectrum of **13** (Figure S9a) showed the presence of one characteristic AB system (two protons at $\delta$ 2.72 and 2.84 ppm that correlates with the same carbon atom at 42.64 ppm). In addition, one exocyclic double bond with carbon atoms at 155.39 ppm (quaternary) and 94.85 (CH), a multiplet with signals at $\delta$ 3.29 and 3.38 ppm ($CH_2$-cycle) that correlates with the same carbon atom at $\delta$ 34.90 ppm and a new singlet at $\delta$ 1.59 ppm (3H) were also observed. In the gHMBC experiment (Figure S9a), long–range correlations between the protons of the $CH_2$-cycle ($\delta$ 3.29 and 3.38 ppm) and the carbons at $\delta$ 27.63 (new $CH_3$), 33.61 (quaternary), 42.64 ($CH_2CO$), 66.98 ($CH$-$SO_2$), 94.85 (CH=) and 155.39 (C=) ppm confirm the proposed structure. This compound showed contrasting results in the ROESY experiment with respect to those observed for the isomer **7**. As mentioned in the main mansucript, in **7** a correlation between the new methyl moiety at $\delta$ 1.44 ppm and the signal at $\delta$ 4.40 ppm ($CH$-$SO_2$) was observed (Figure S9b). This correlation, that indicates

that the new $CH_3$ is on the upper side of the furanose ring, was not observed in **13** suggesting an opposite configuration for the stereogenic center present on this compound.

*2-Dimensional NMR procedures*

Proton and carbon assignments were based on COSY, gradient-HSQC (gHSQC), gradient-HMBC (gHMBC), NOESY and ROESY experiments. Homonuclear 2D spectra (COSY, NOESY and ROESY) were collected in a 1024 x 256 matrix with a spectral width between 3000 and 6000 Hz and a 1 s recycle delay, and processed in a 1024 x 1024 matrix. They were carried out with 16 or 32 transients for each of 256 or 512 time increments. Mixing time was 0.3 s for ROESY. 2D Inverse proton detected heteronuclear shift correlation spectra (gHSQC and gHMBC) were obtained with the following conditions: the same $^{1}H$ spectral window, a $^{13}C$ spectral window of 25000 Hz for gHSQC and 30000 Hz for gHMBC, 1 s of relaxation delay and 128 or 200 time increments. Typical numbers of transients per increment were 4 and 16. Data were collected in a 1024 x 256 matrix and processed in a 1024 x 1024 matrix. The gHSQC experiment was optimized for one bond heteronuclear coupling constant of 145 Hz. The gHMBC experiment was optimized for long range coupling constants of 8 Hz.

*Selected copies of NMR spectra*

## FIGURE S10. Compound 5 ($^{1}$H-NMR)

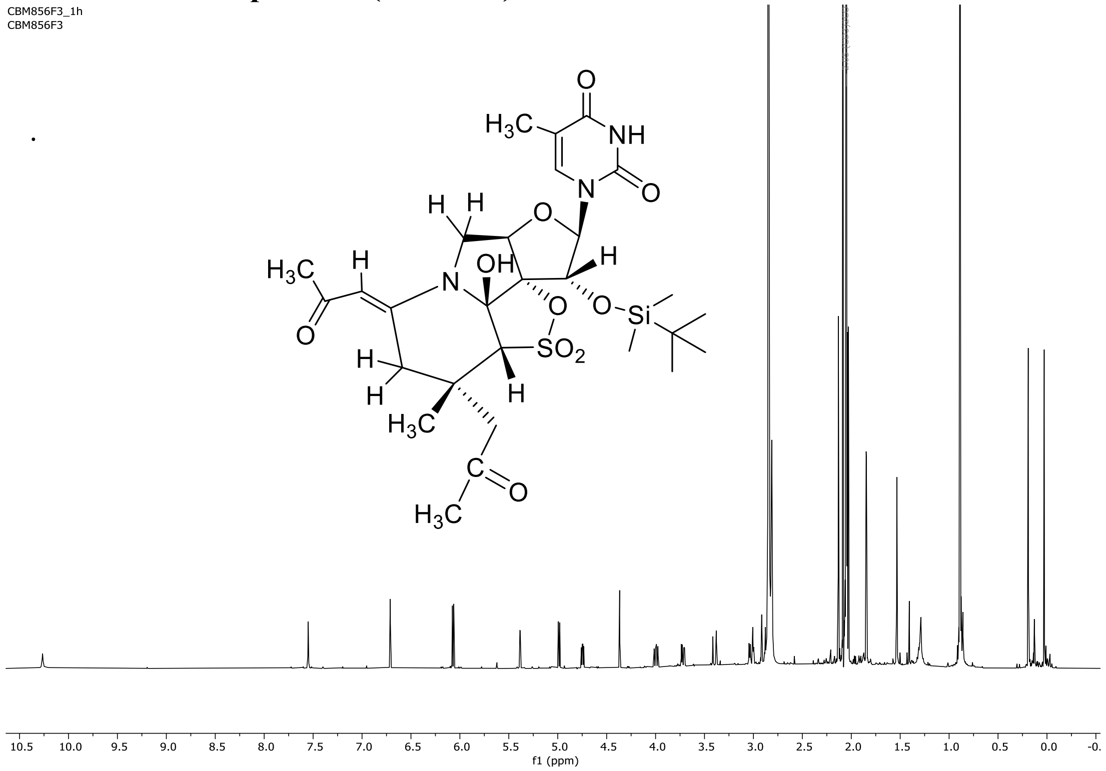

## FIGURE S11. Compound 5 ($^{13}$C-NMR)

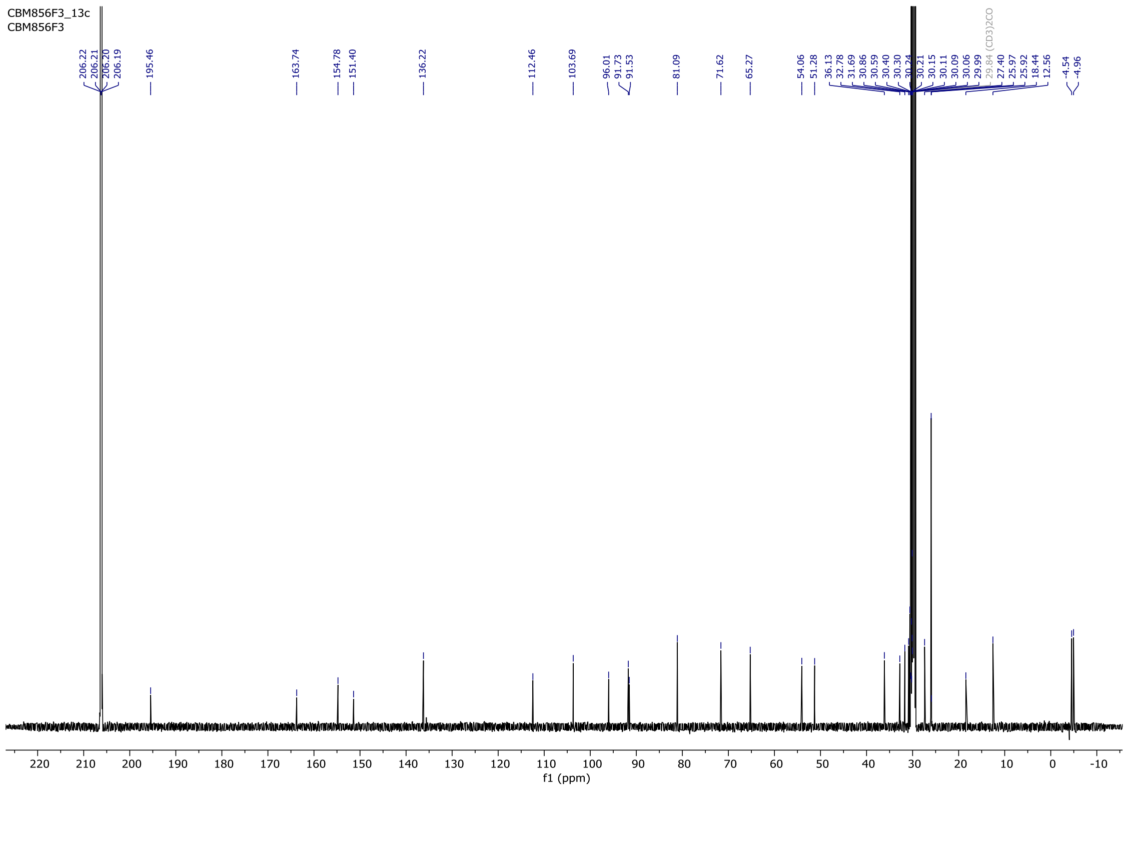

**FIGURE S12. Compound 5 (HSQC)**

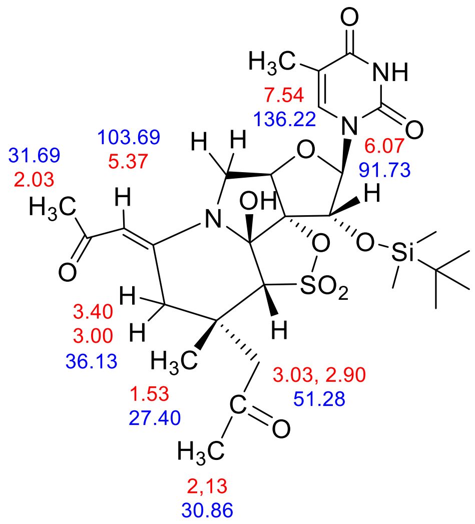


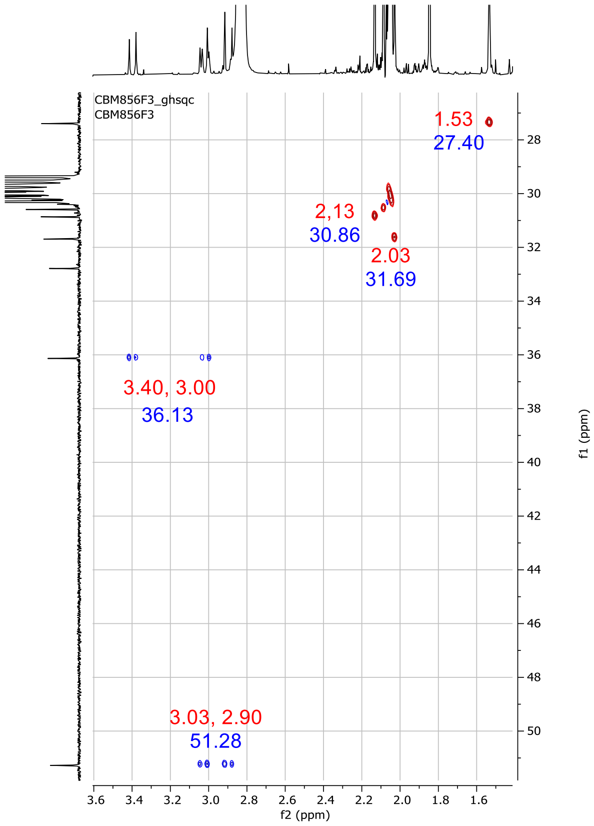


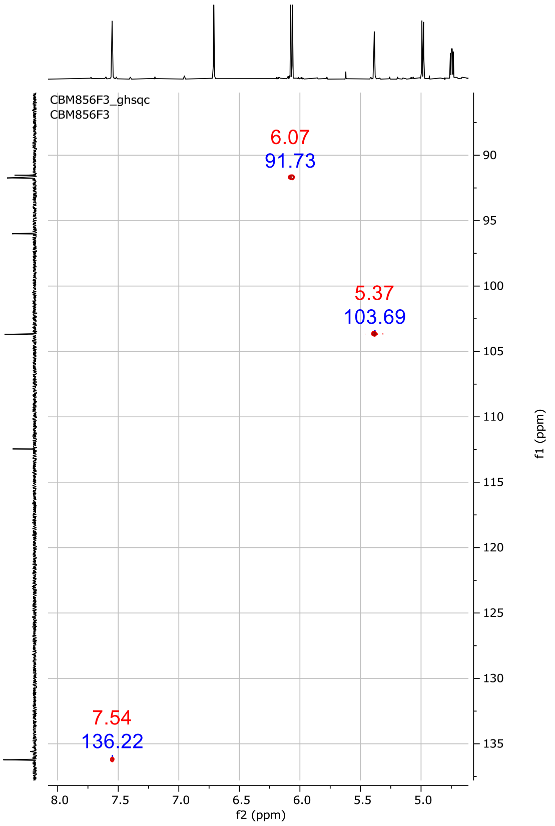

**FIGURE S13. Compound 5 (HMBC)**

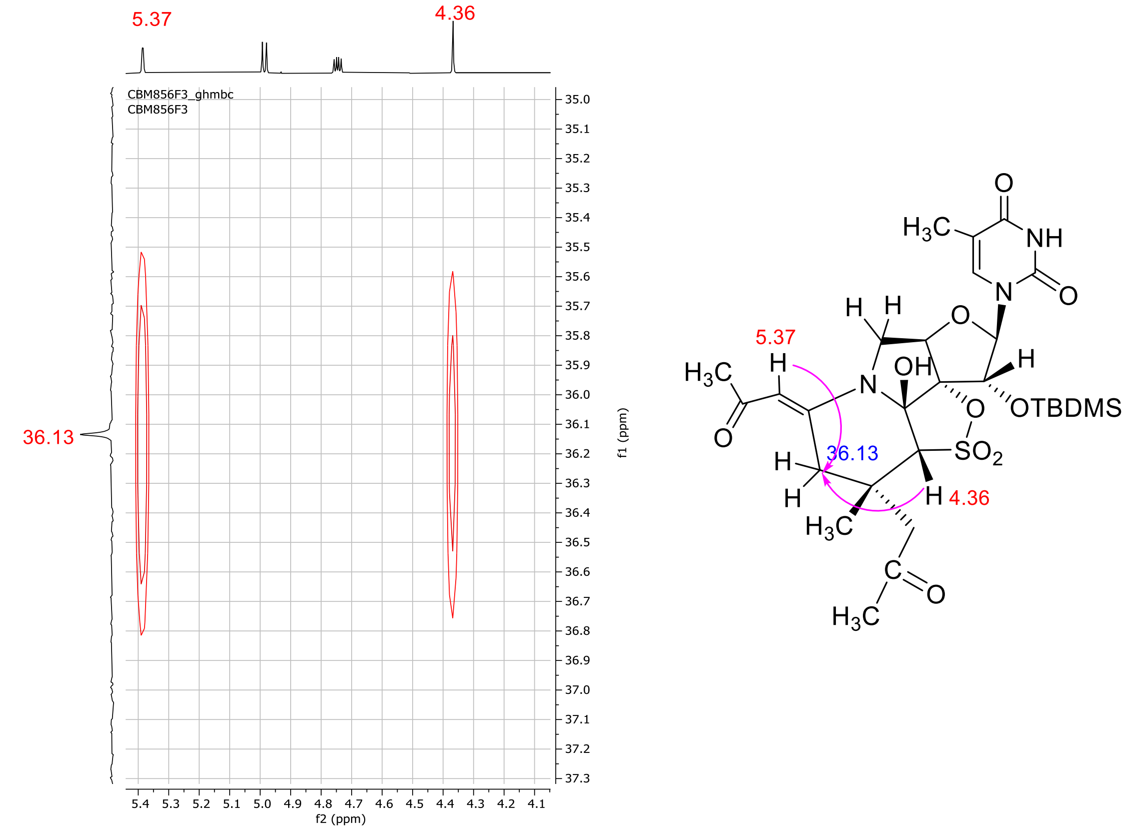


**FIGURE S14. Compound 5 (ROESY)**

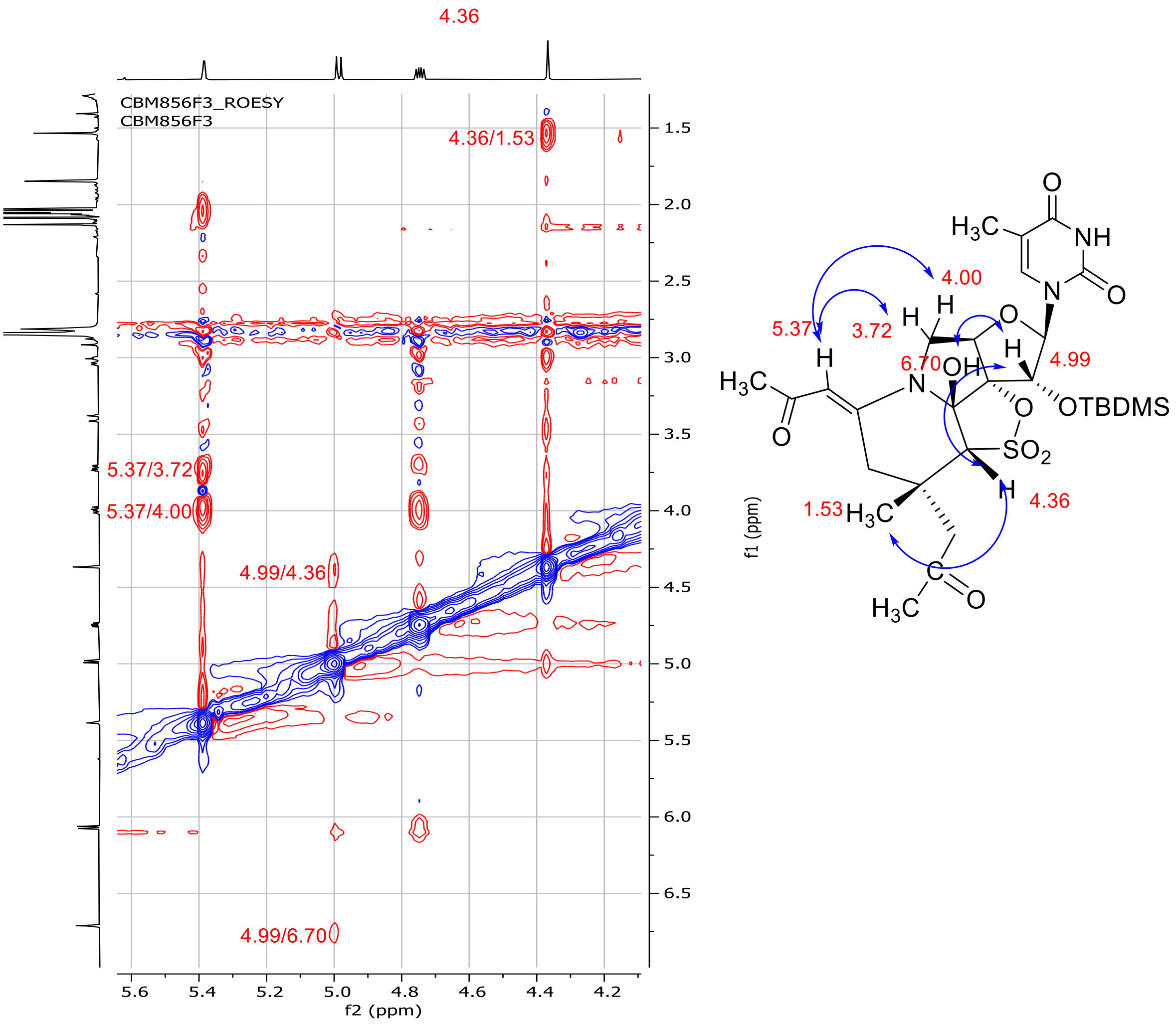

## FIGURE S15. Compound 6 ($^{1}$H-NMR)

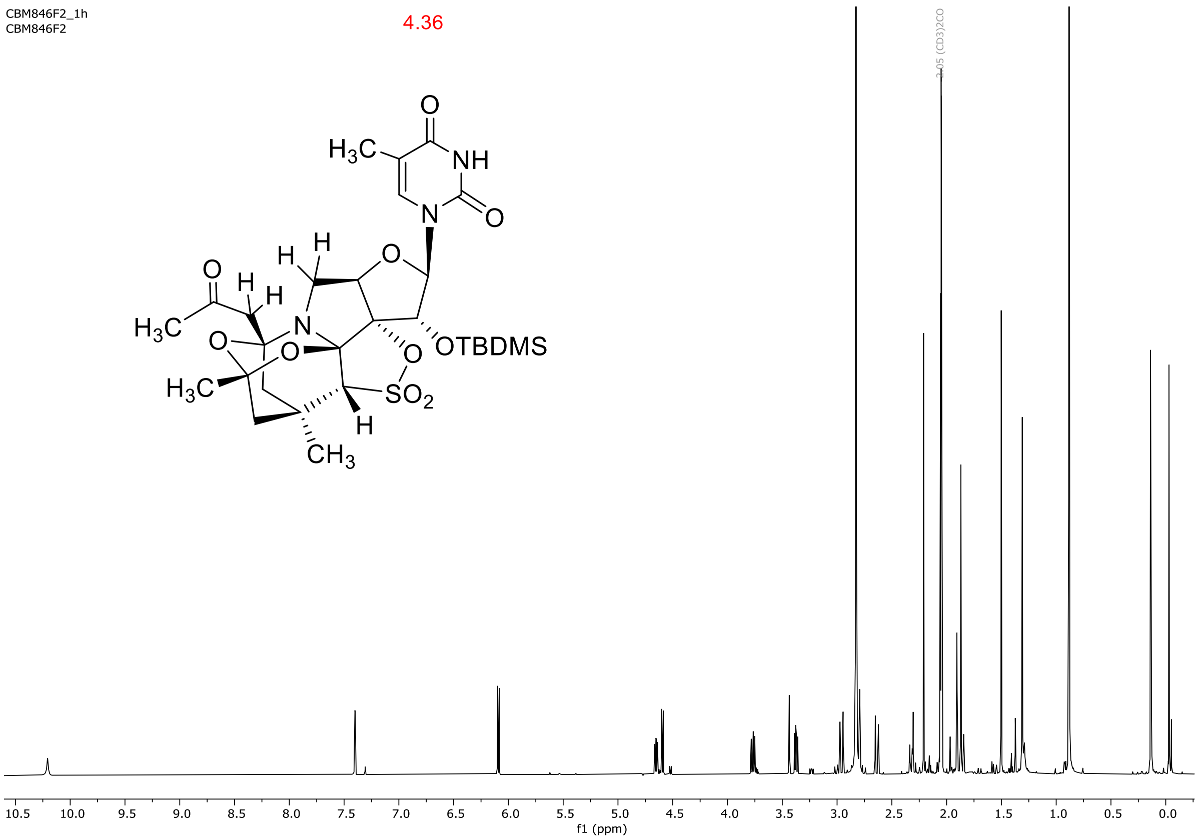


## FIGURE S16. Compound 6 ($^{13}$C-NMR)

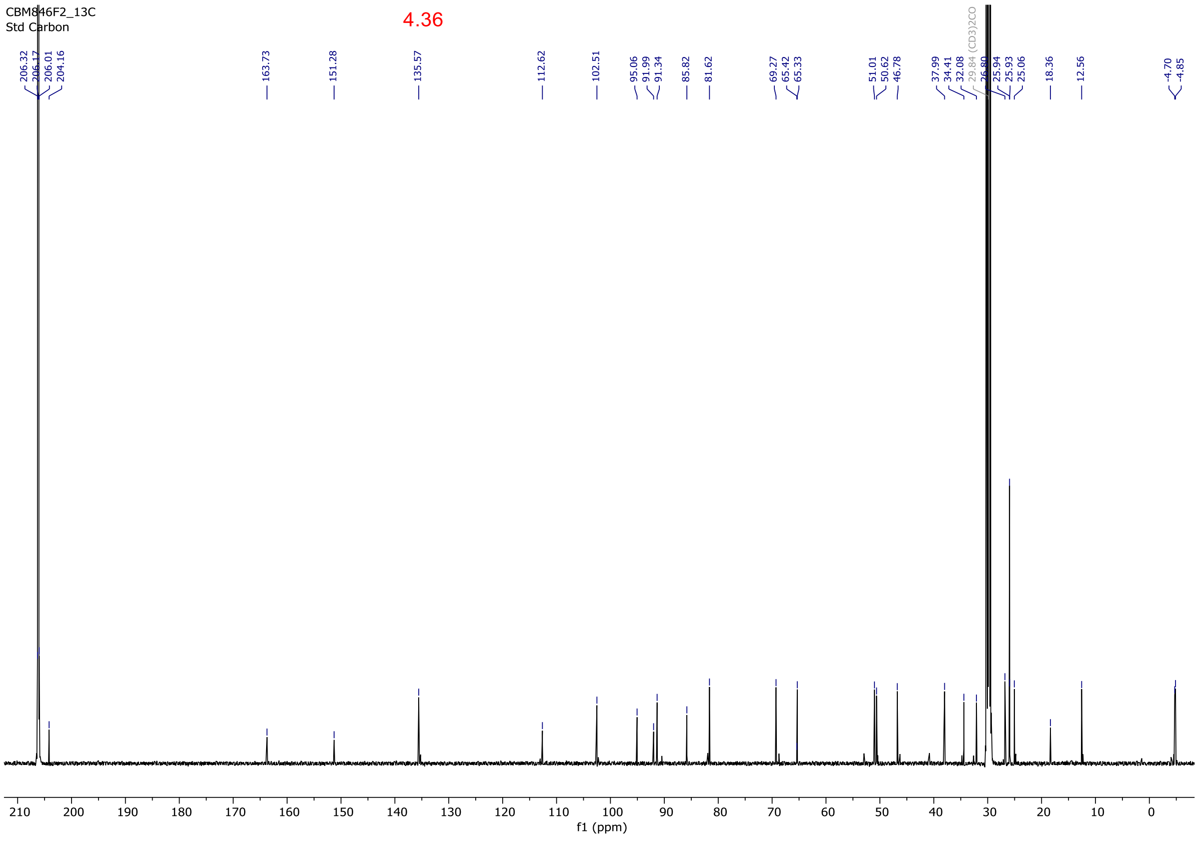

## FIGURE S17. Compound 6 (HSQC)

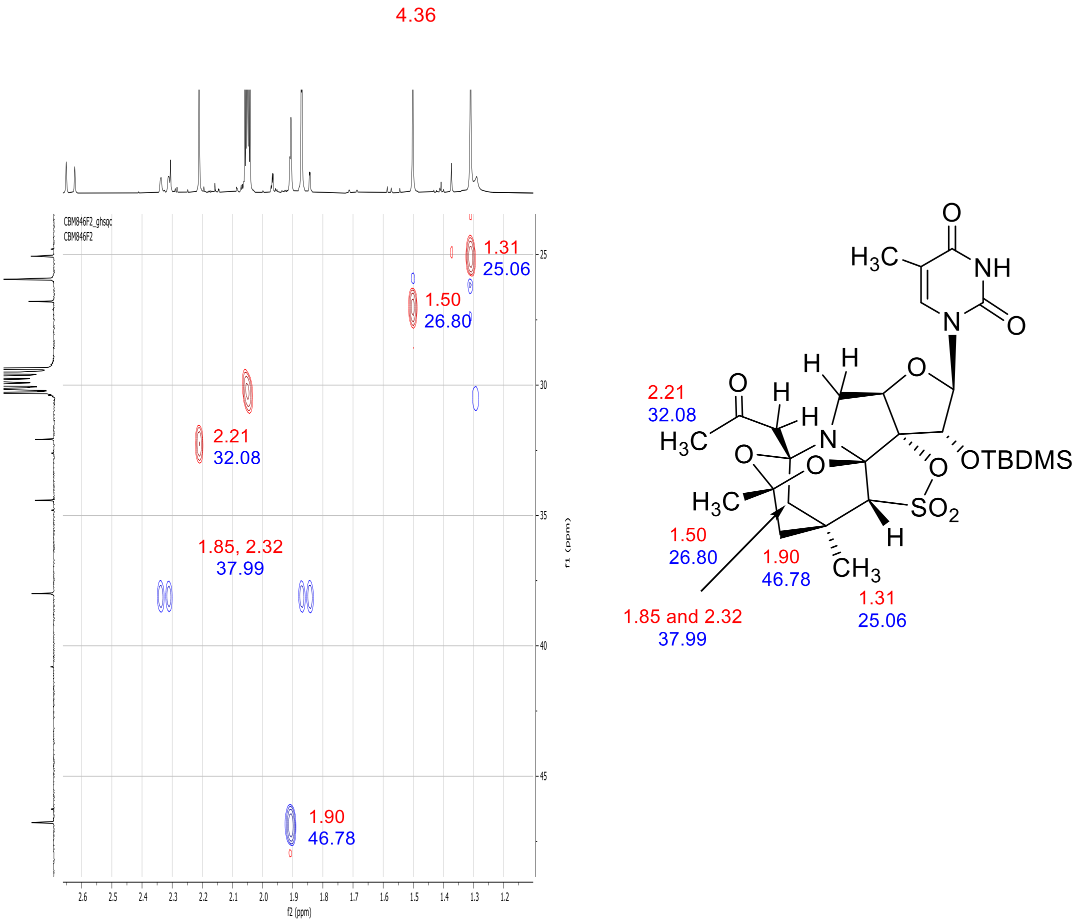


## FIGURE S18. Compound 6 (HMBC)

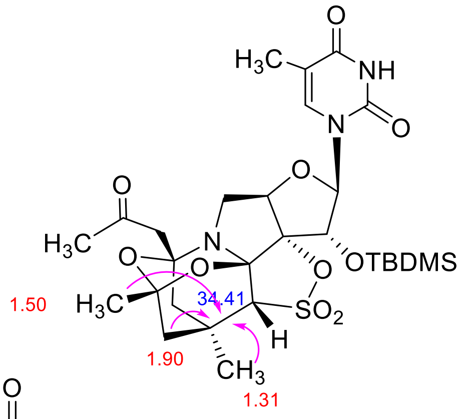
O
H3C
NH
N
O
O
H3C
N
O
O
OTBDMS
1.50
H3C
34.41
SO2
H
1.90
CH3
1.31

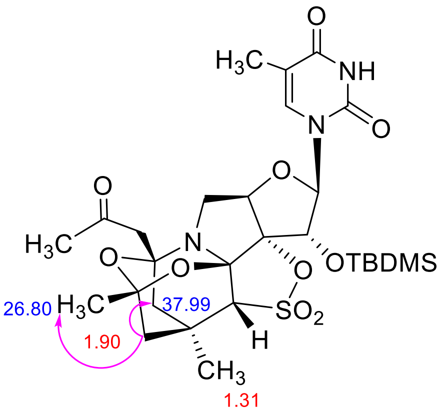
O
H3C
NH
N
O
O
O
H3C
N
O
O
OTBDMS
26.80
H3C
37.99
O
SO2
1.90
H
CH3
1.31

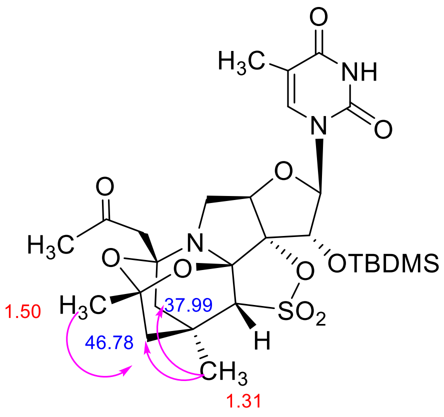
O
H3C
NH
N
O
O
O
H3C
N
O
O
OTBDMS
1.50
H3C
37.99
O
SO2
46.78
H
CH3
1.31

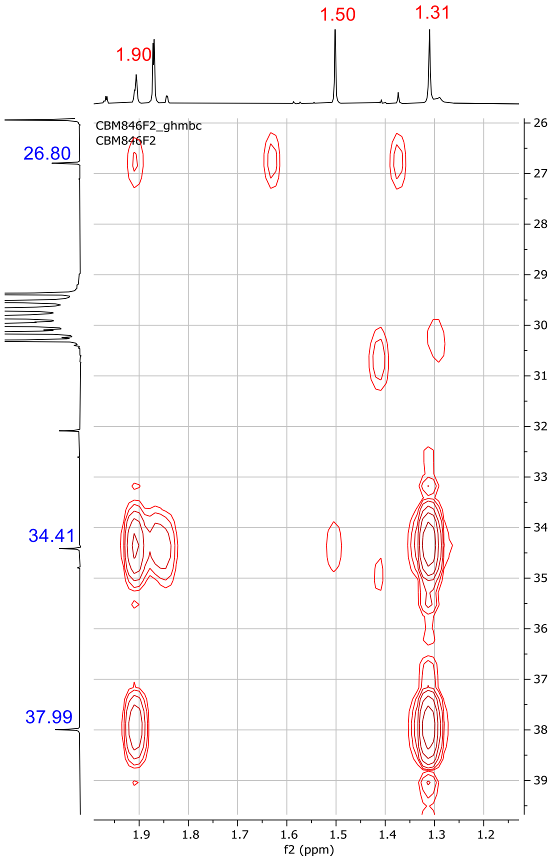
1.90
1.50
1.31
CBM846F2_ghmbc
CBM846F2
26.80
34.41
37.99
f2 (ppm)

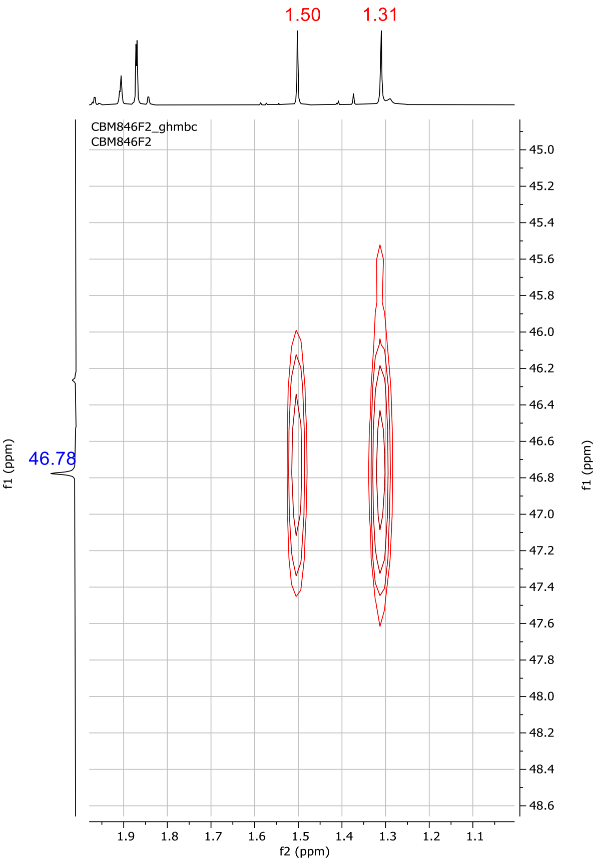
1.50
1.31
CBM846F2_ghmbc
CBM846F2
46.78
f1 (ppm)
f2 (ppm)

**FIGURE S19. Compound 6 (ROESY)**

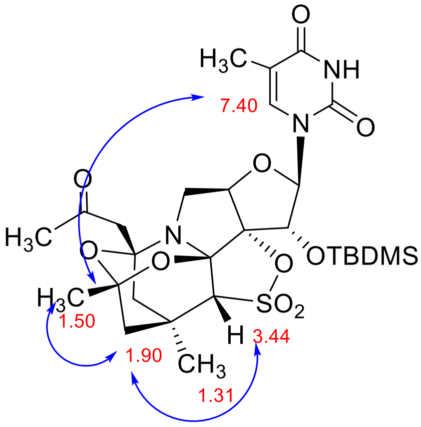


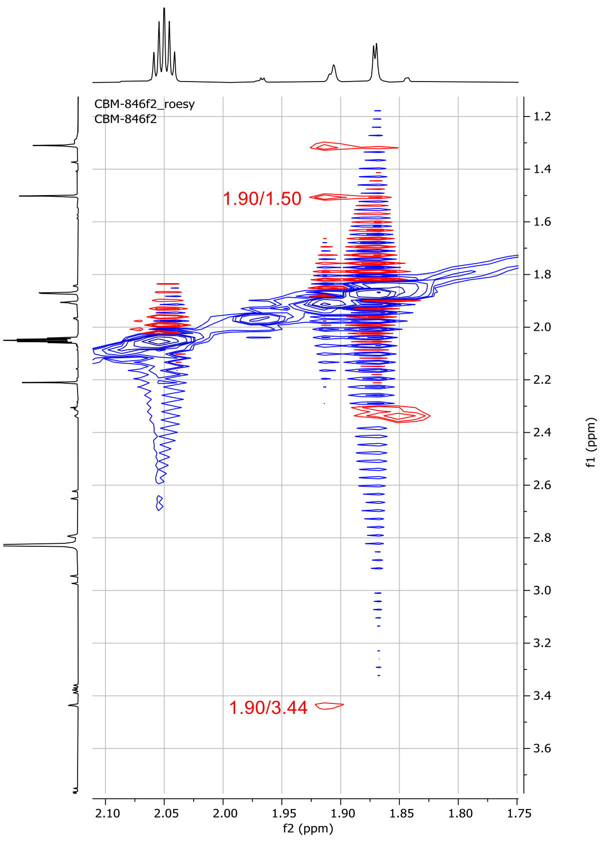


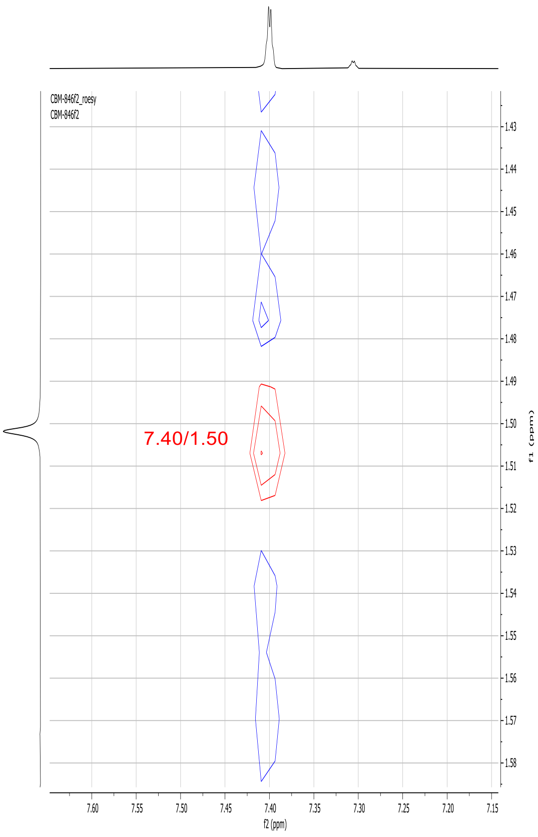

## FIGURE S20. Compound 7 ($^{1}$H-NMR)

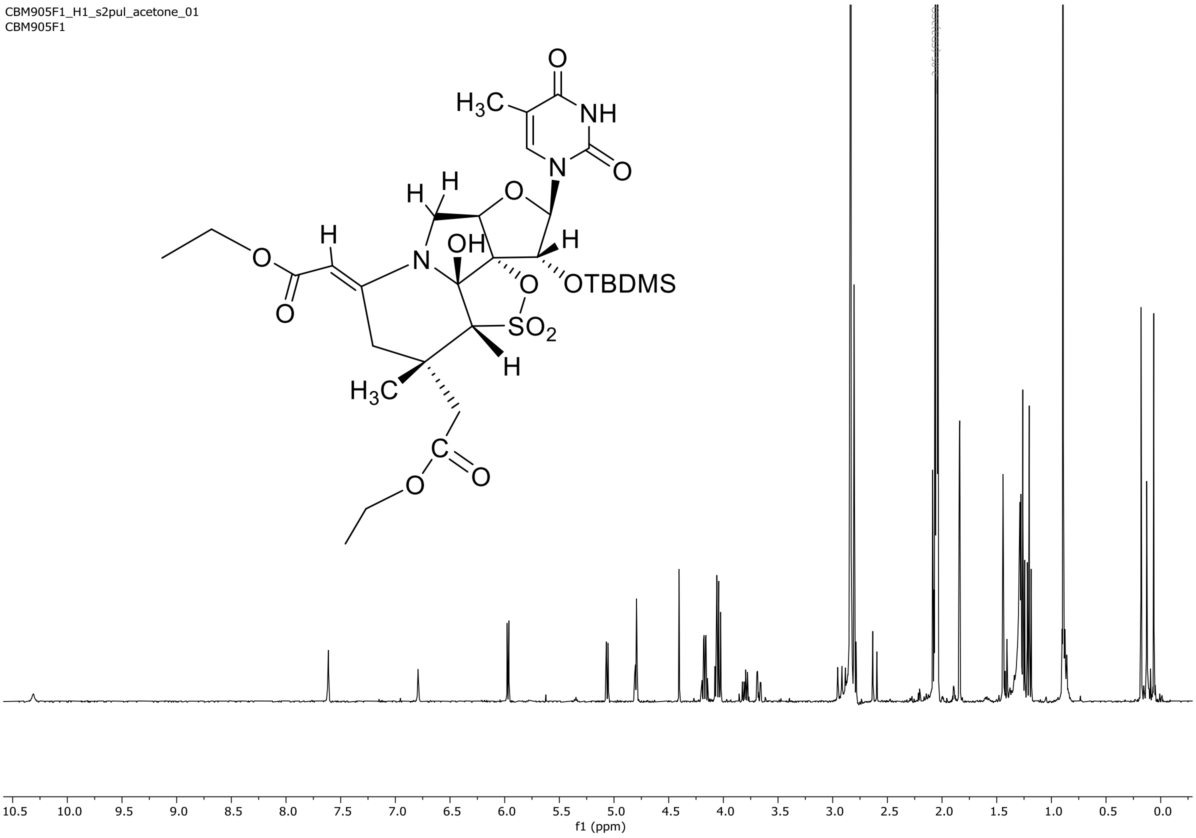


## FIGURE S21. Compound 7 ($^{13}$C-NMR)

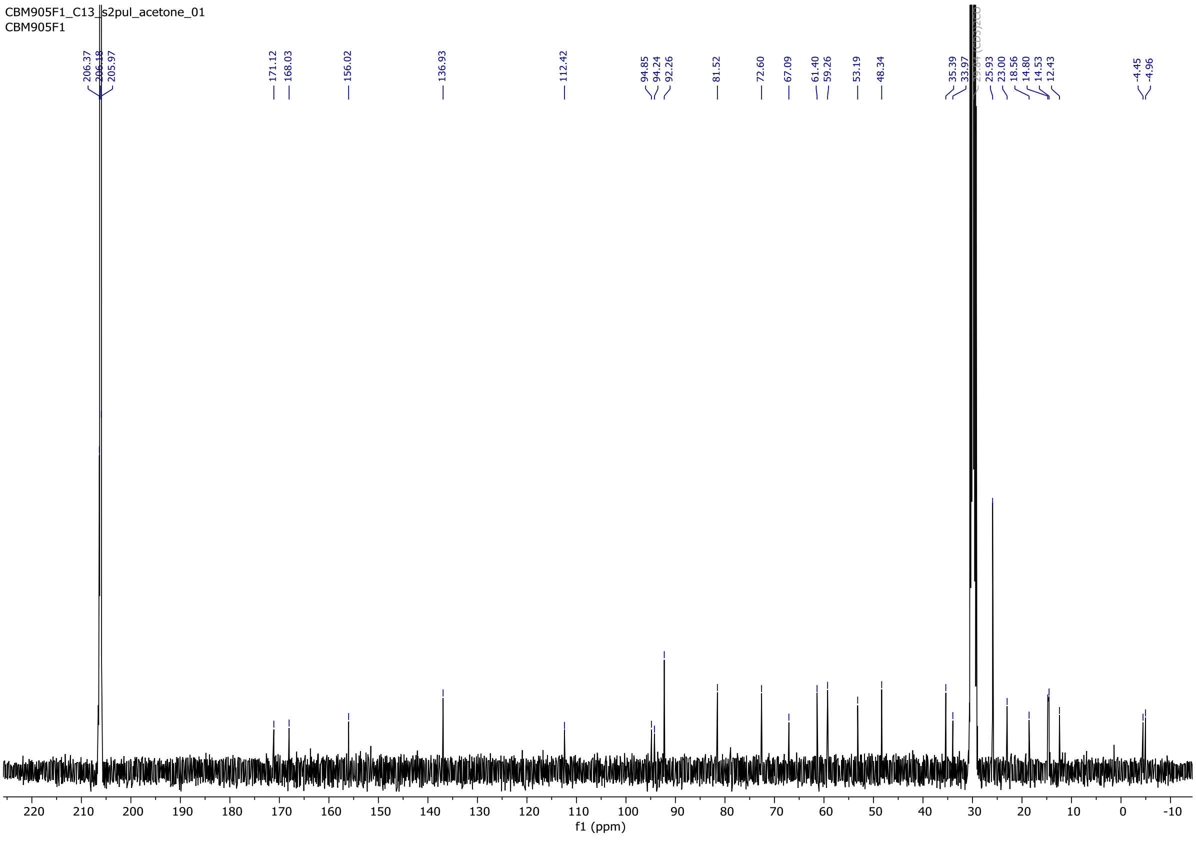

# FIGURE S22. Compound 7 (HSQC)

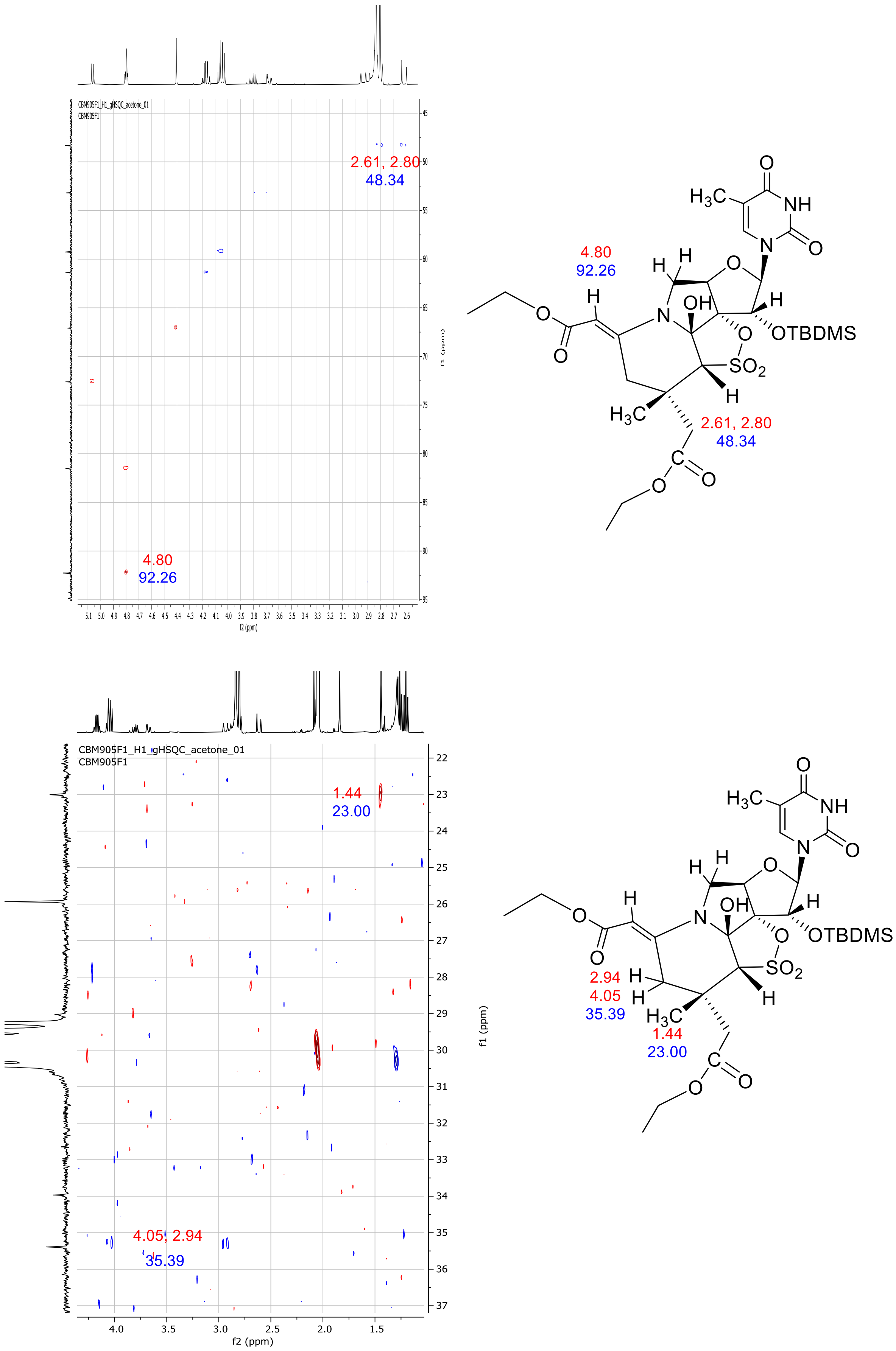

**FIGURE S23. Compound 7 (HMBC and ROESY)**

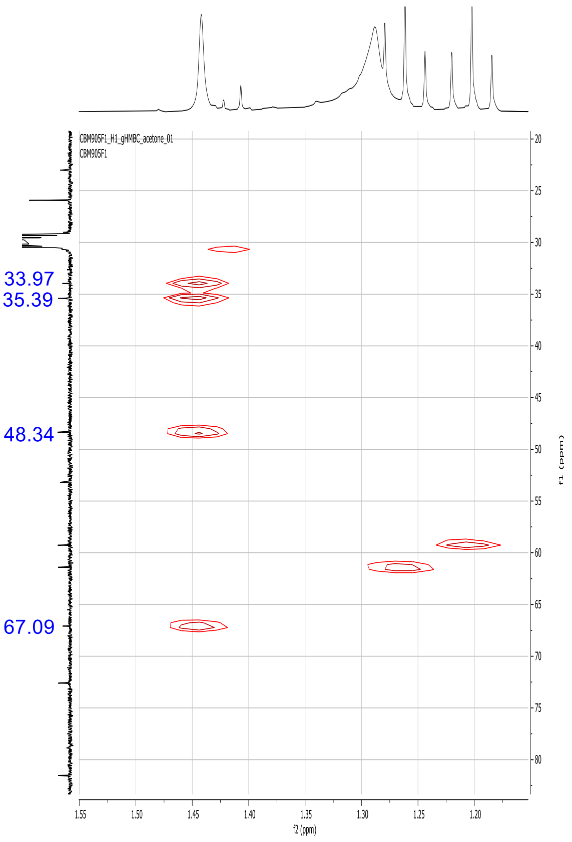


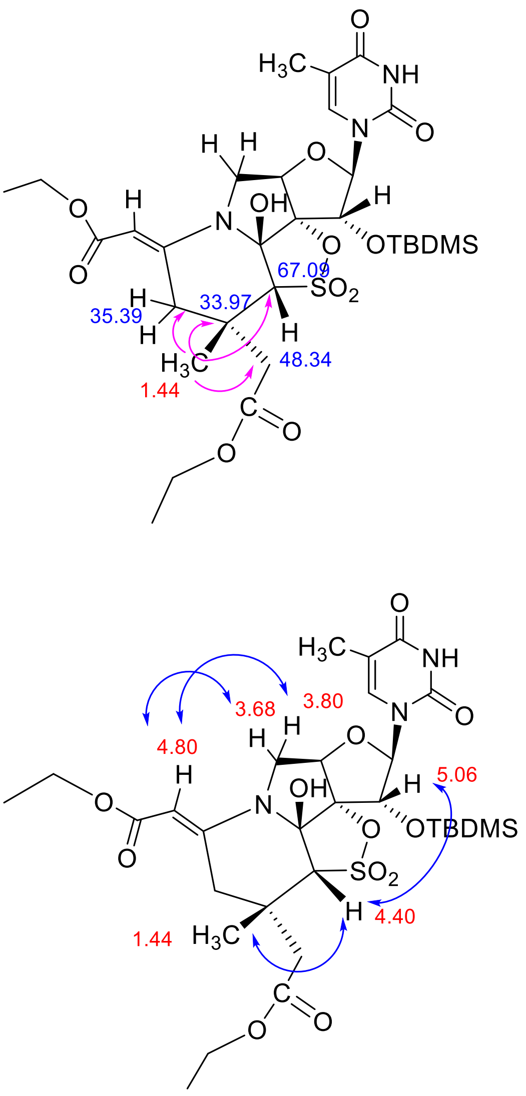


**ROESY**

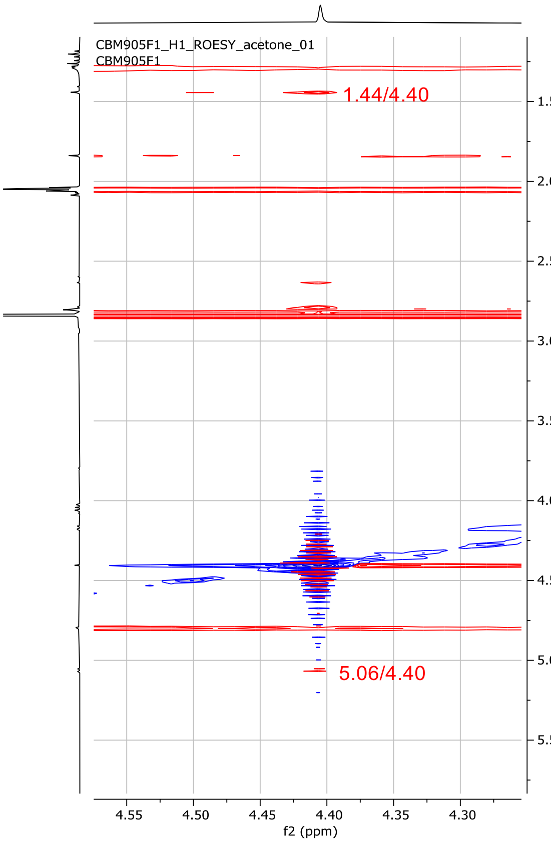


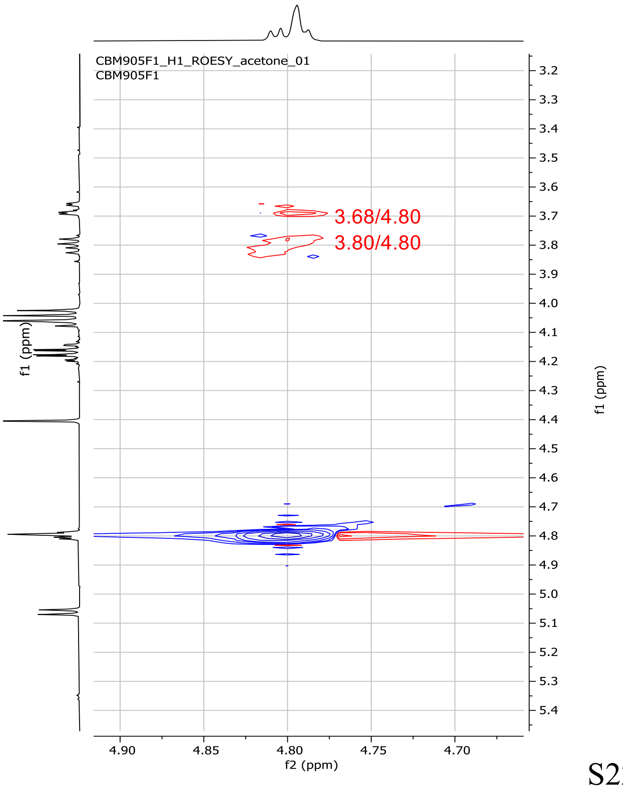

# FIGURE S24. Compound 8 (¹H-NMR)

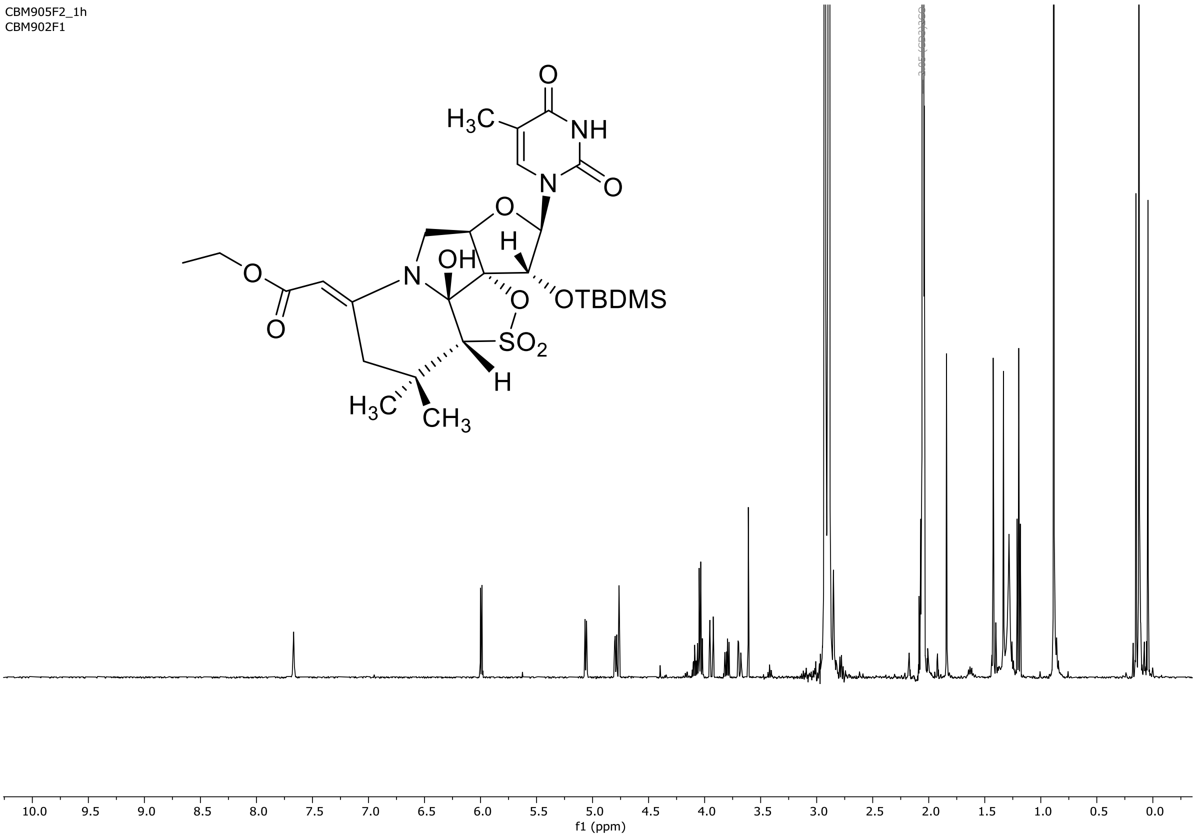


# FIGURE S25. Compound 8 (¹³C-NMR)

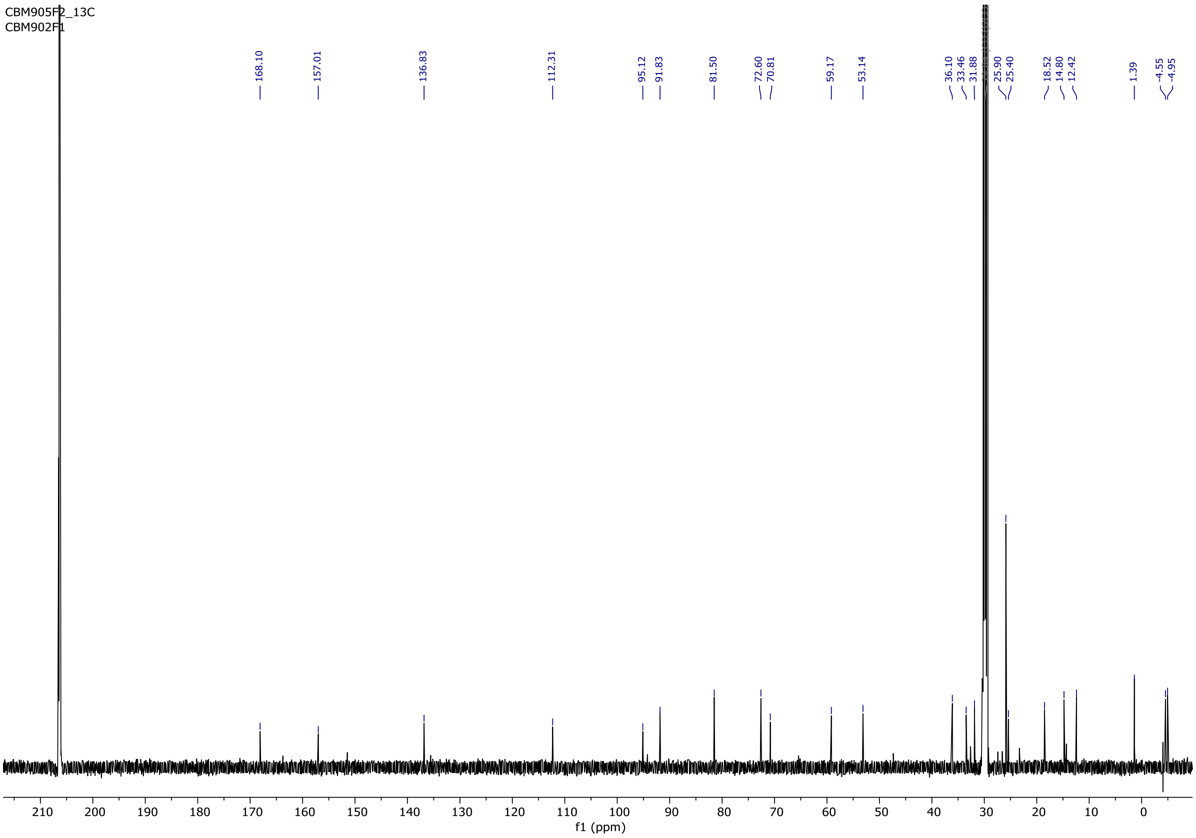

## FIGURE S26. Compound 8 (HMBC and NOESY)

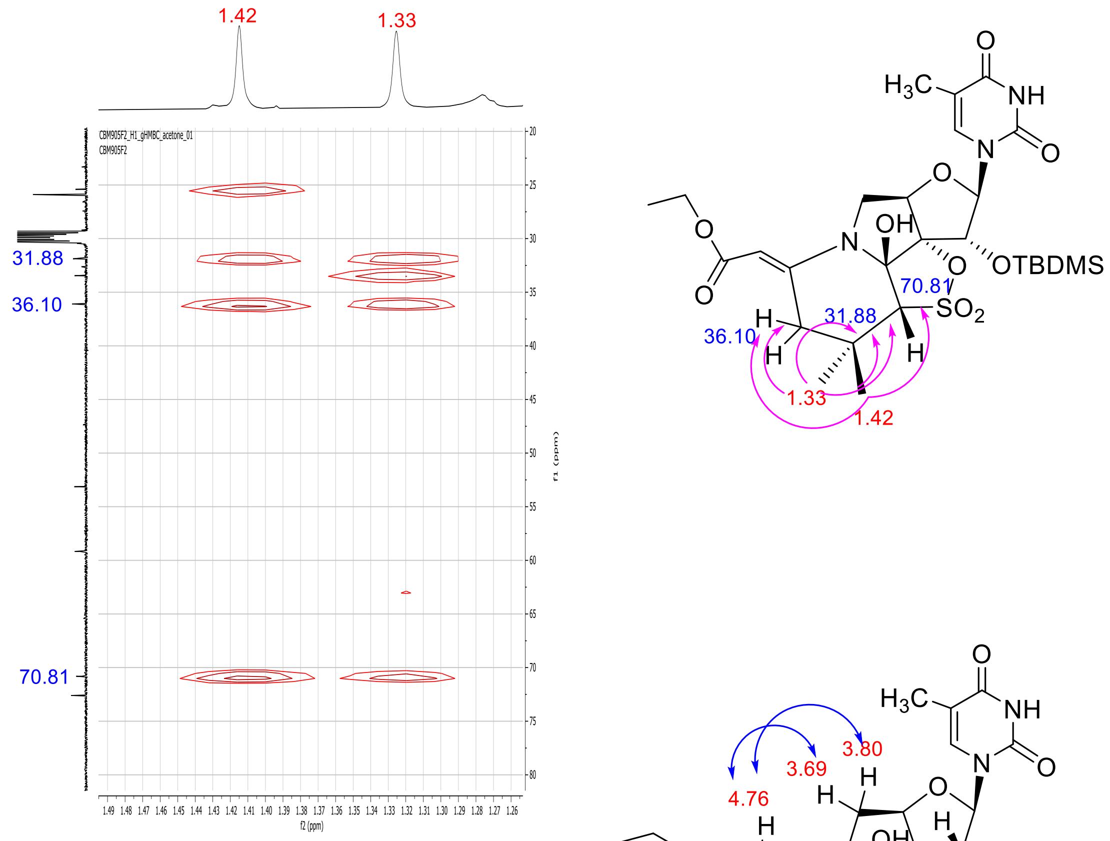


## NOESY

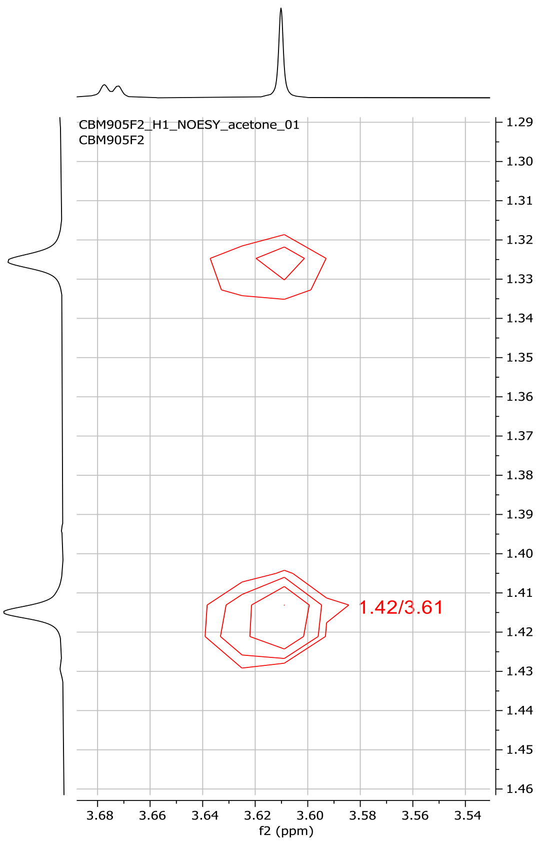


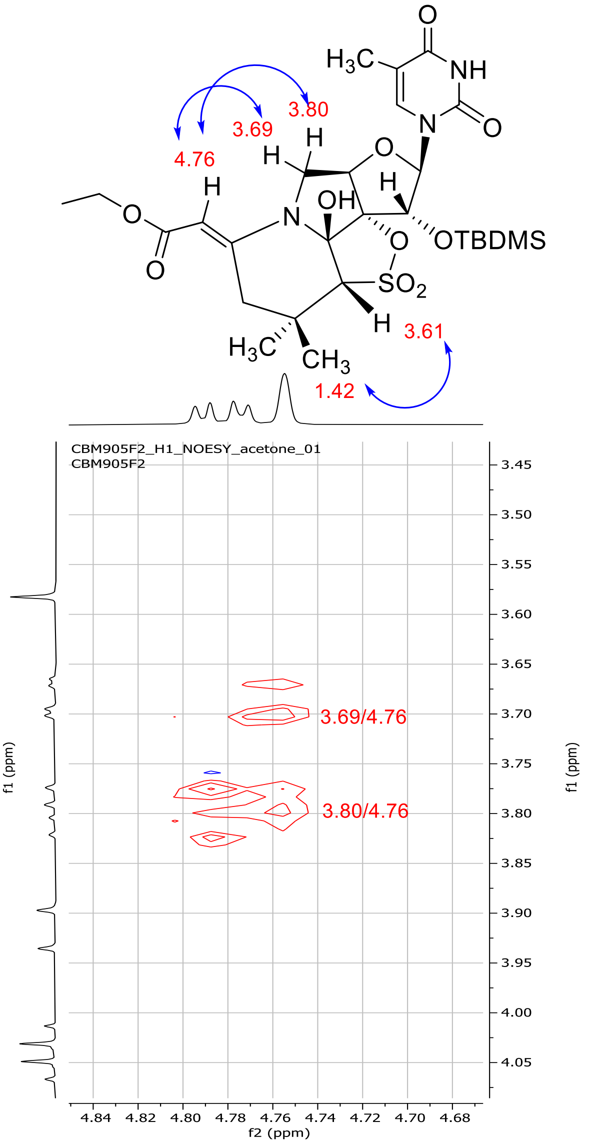

# FIGURE S27. Compound 9 ($^1$H-NMR)

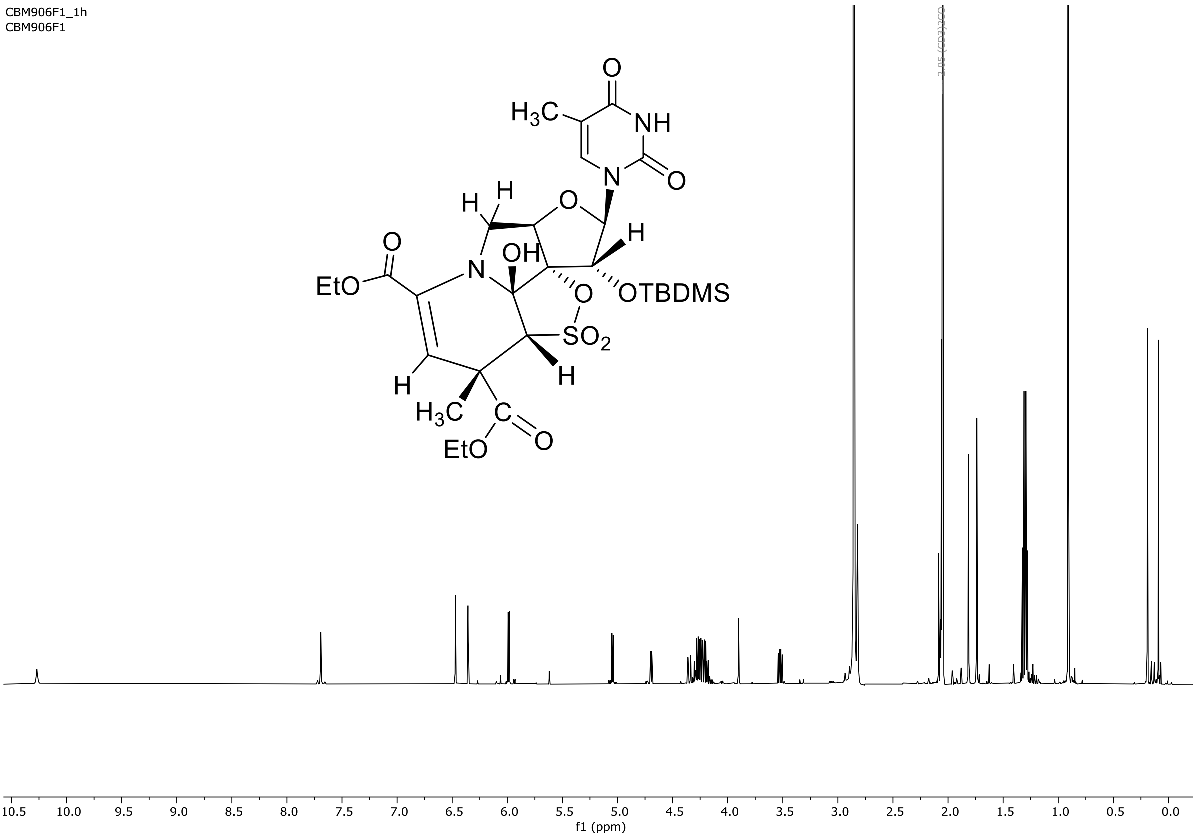


# FIGURE S28. Compound 9 ($^{13}$C-NMR)

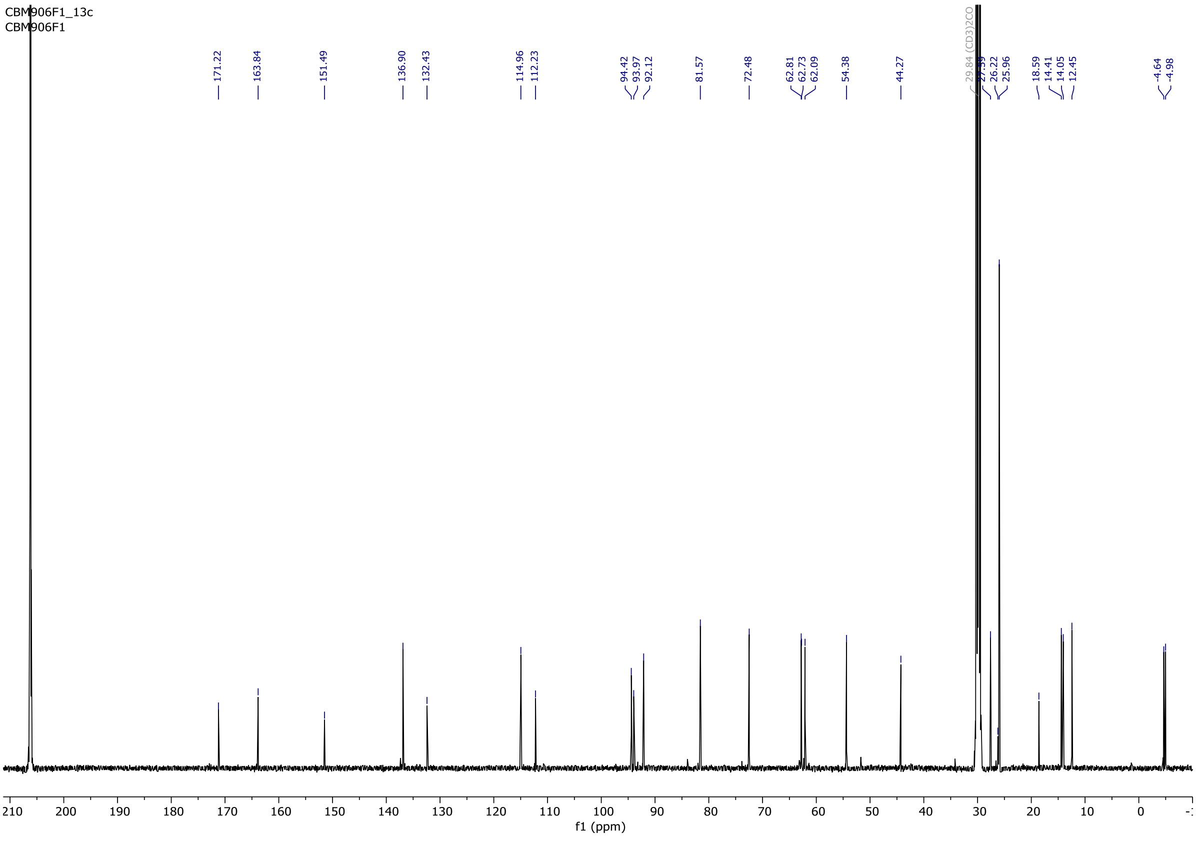

**FIGURE S29. Compound 9 (HMBC)**

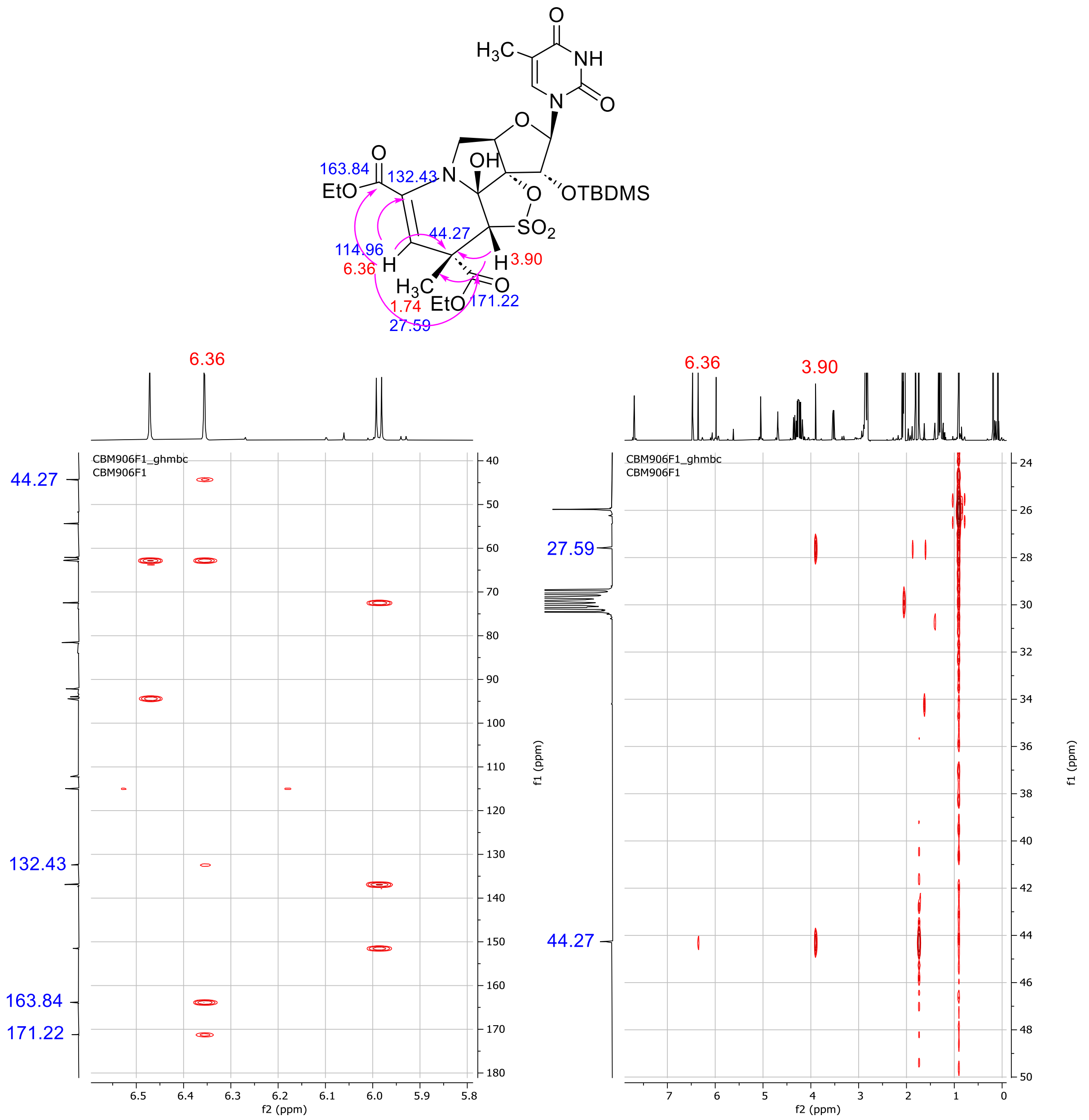

# FIGURE S30. Compound 9 (ROESY)

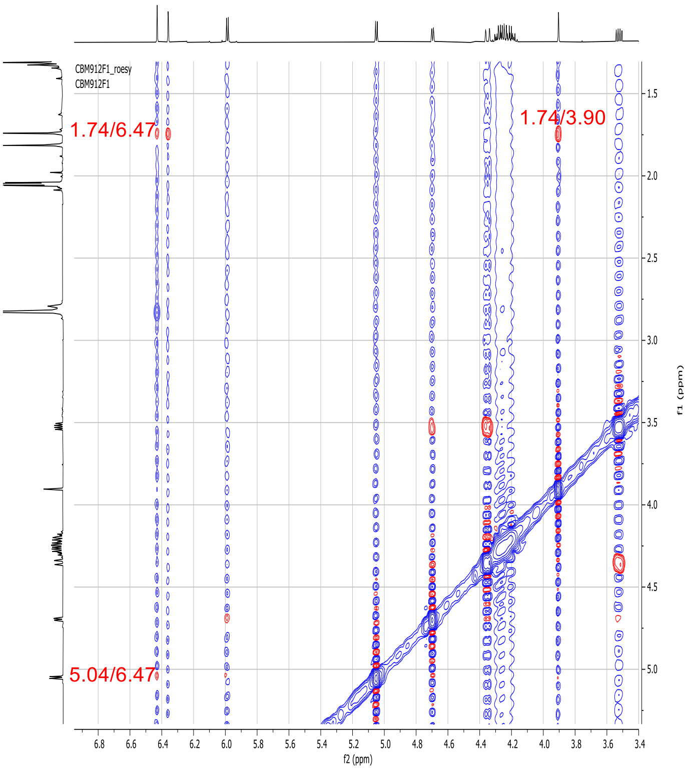


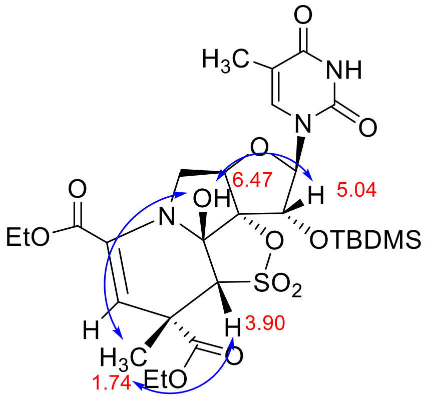

## FIGURE S31. Compound 10 ($^{1}$H-NMR)

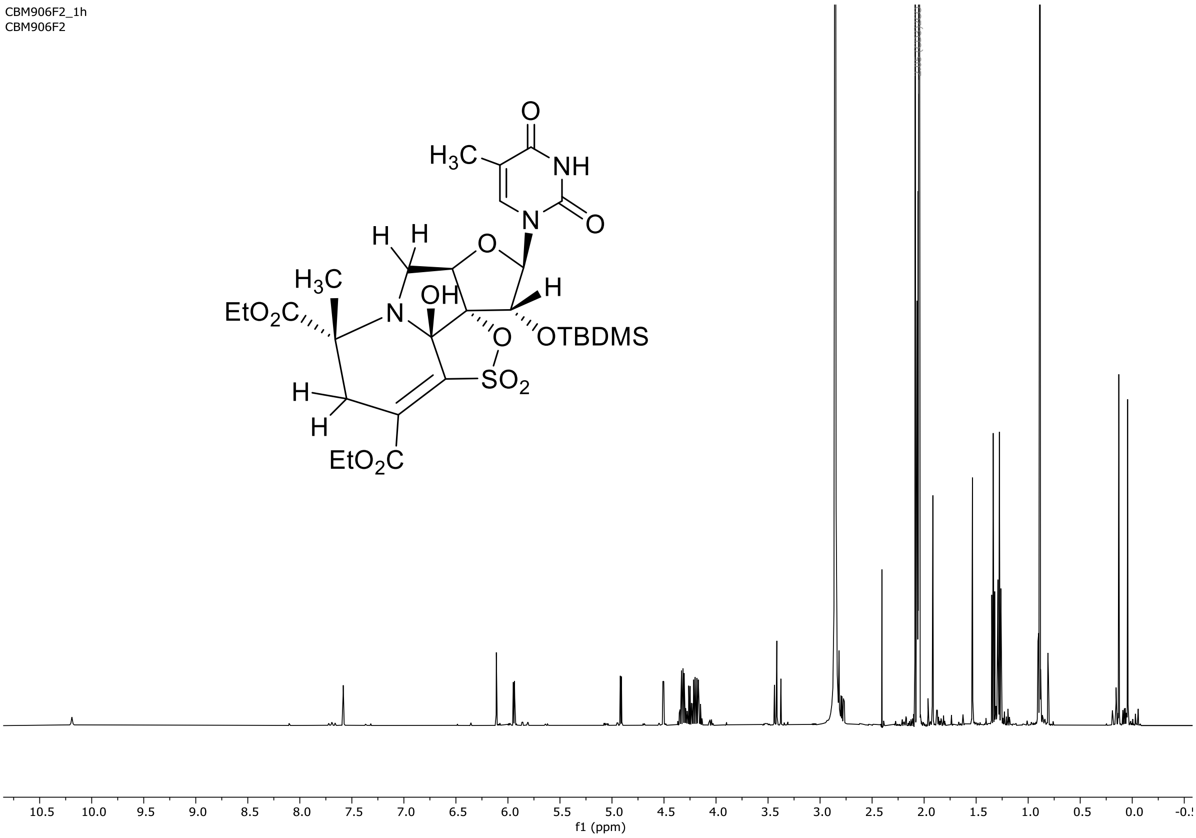


## FIGURE S32. Compound 10 ($^{13}$C-NMR)

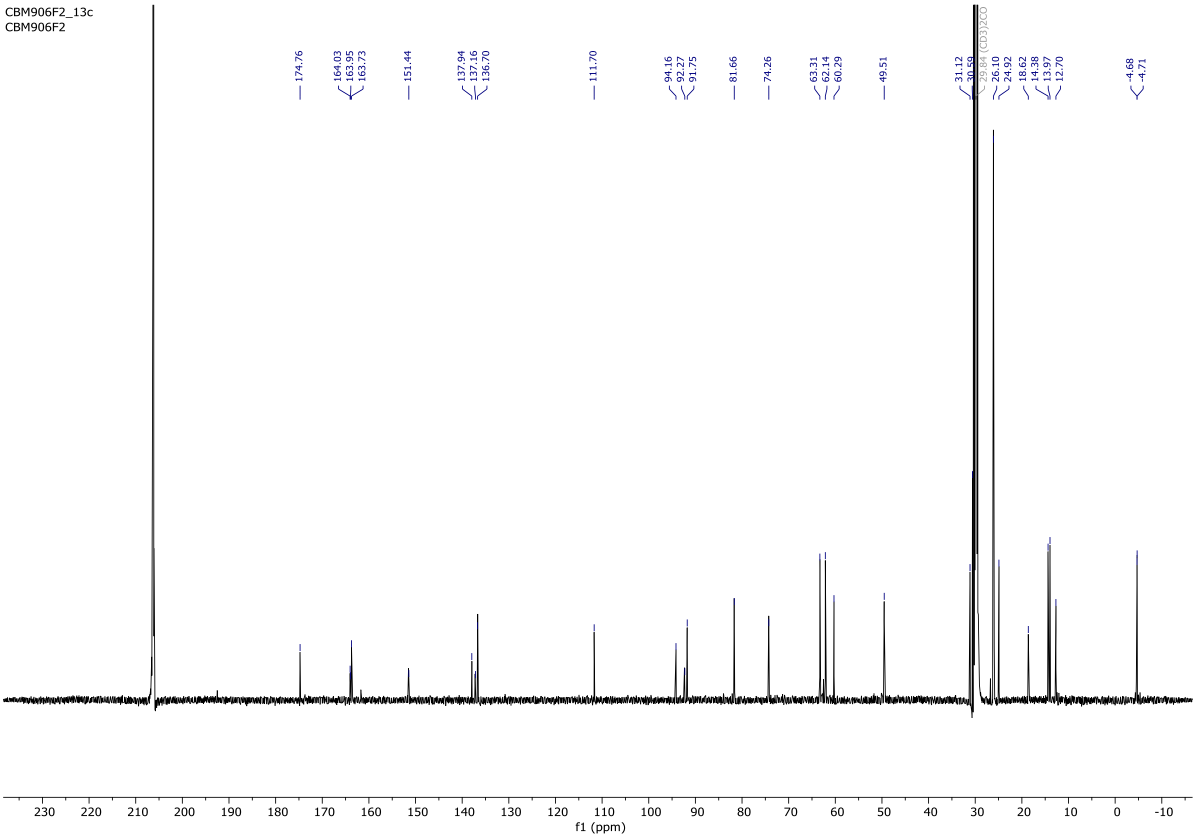

**FIGURE S33. Compound 10 (HSQC and HMBC)**

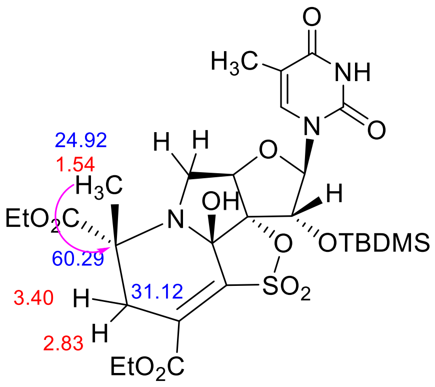


## HSQC

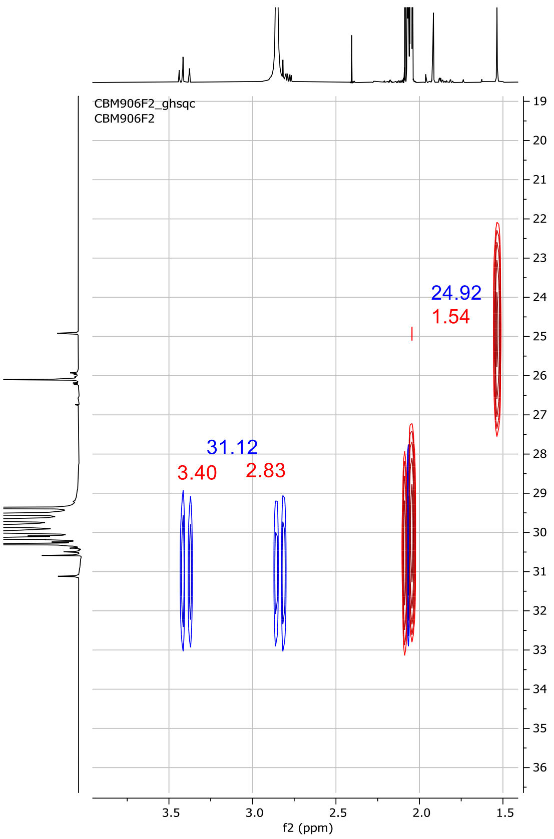


## HMBC

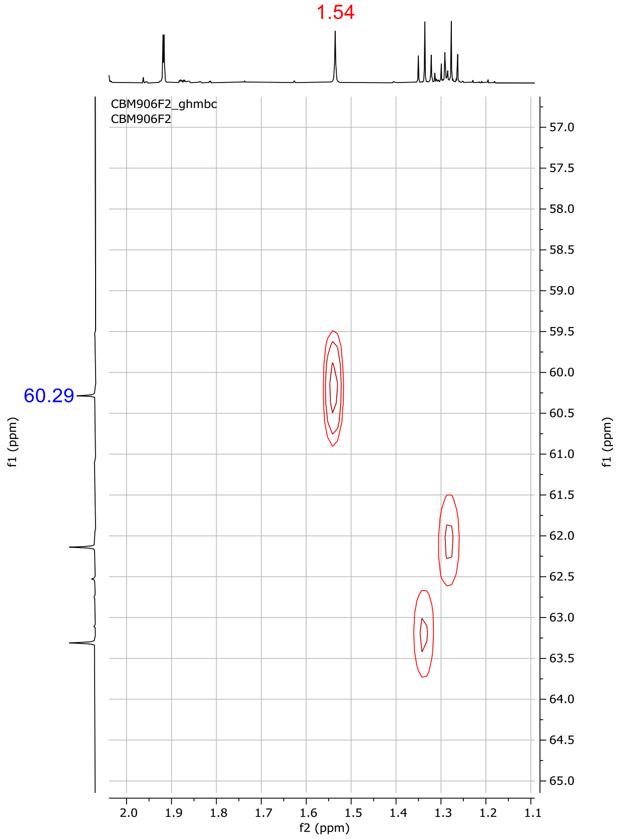

**FIGURE S34. Compound 10 (ROESY)**

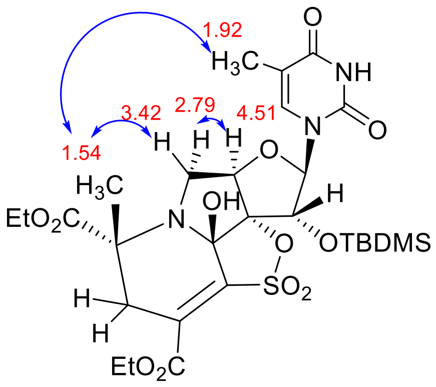


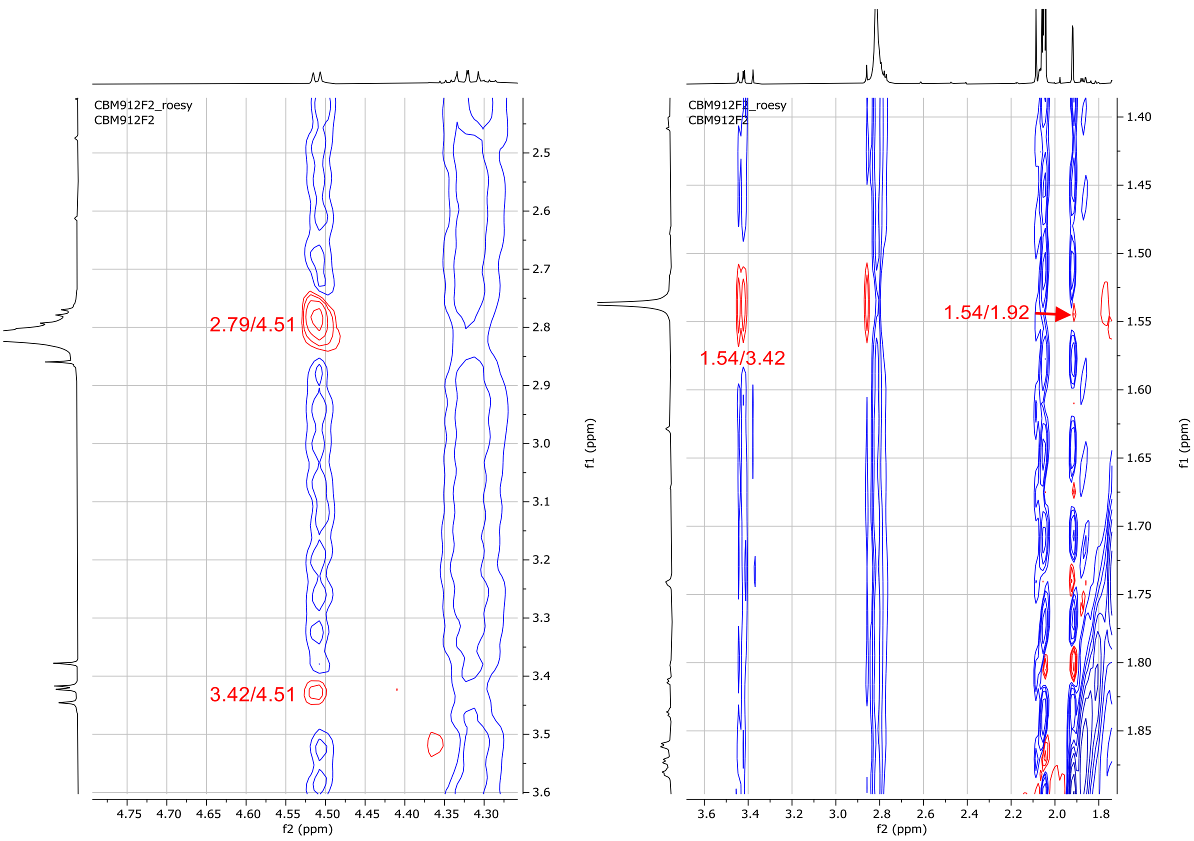

## FIGURE S35. Compound 11 ($^{1}$H-NMR)

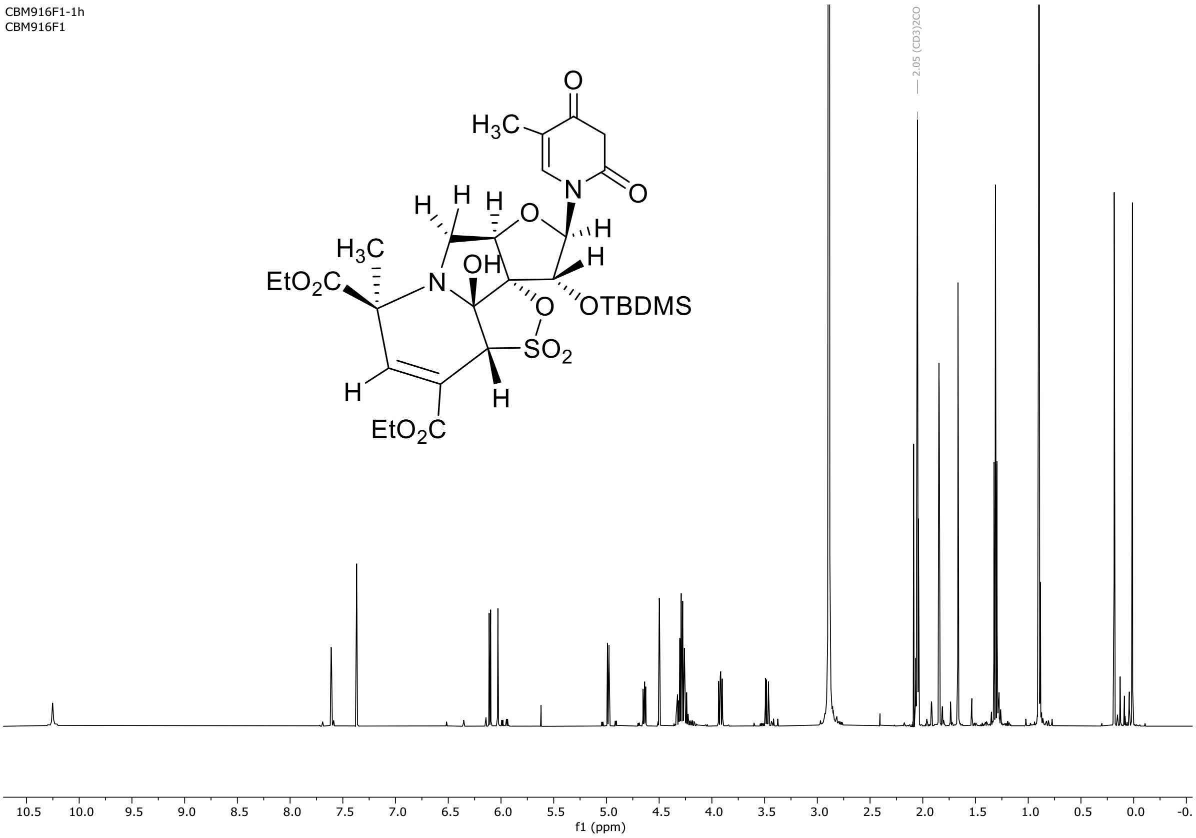


## FIGURE S36. Compound 11 ($^{13}$C-NMR)

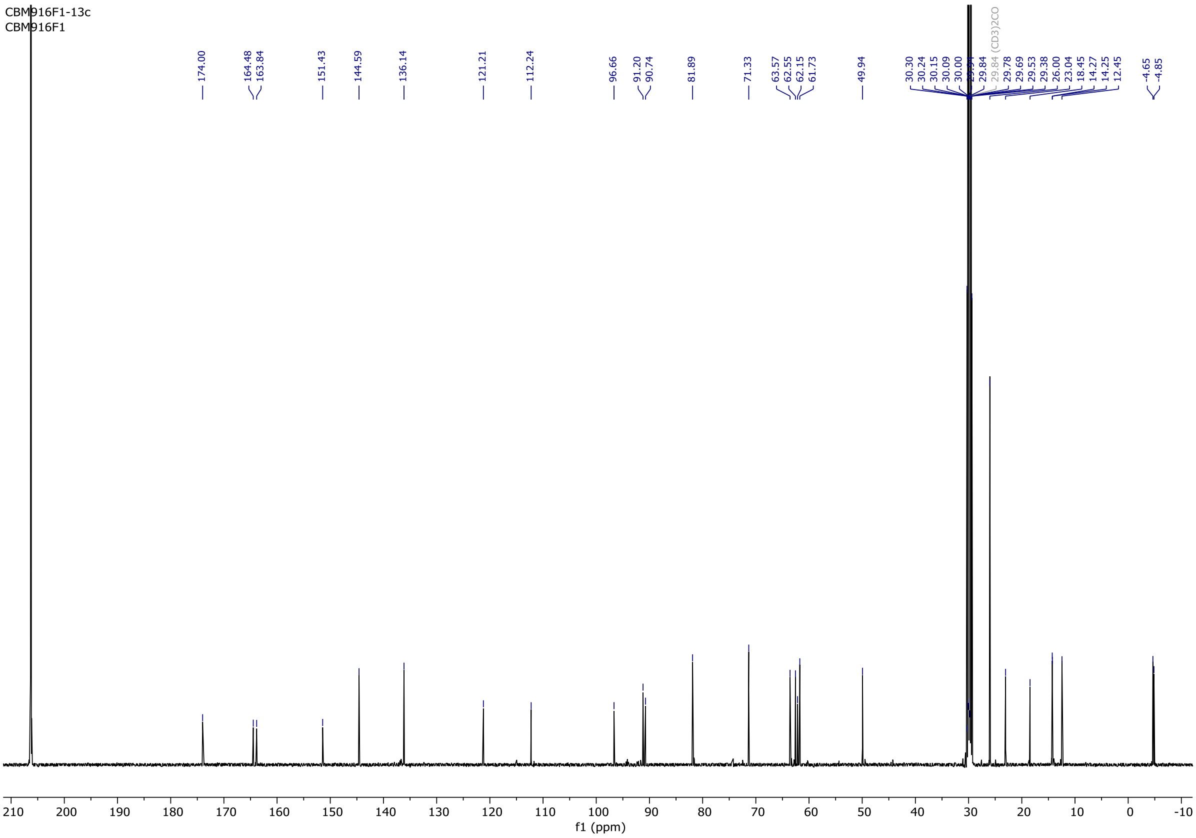

**FIGURE S37. Compound 11 (HMBC)**

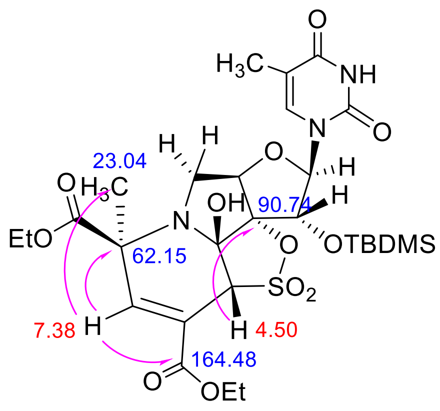


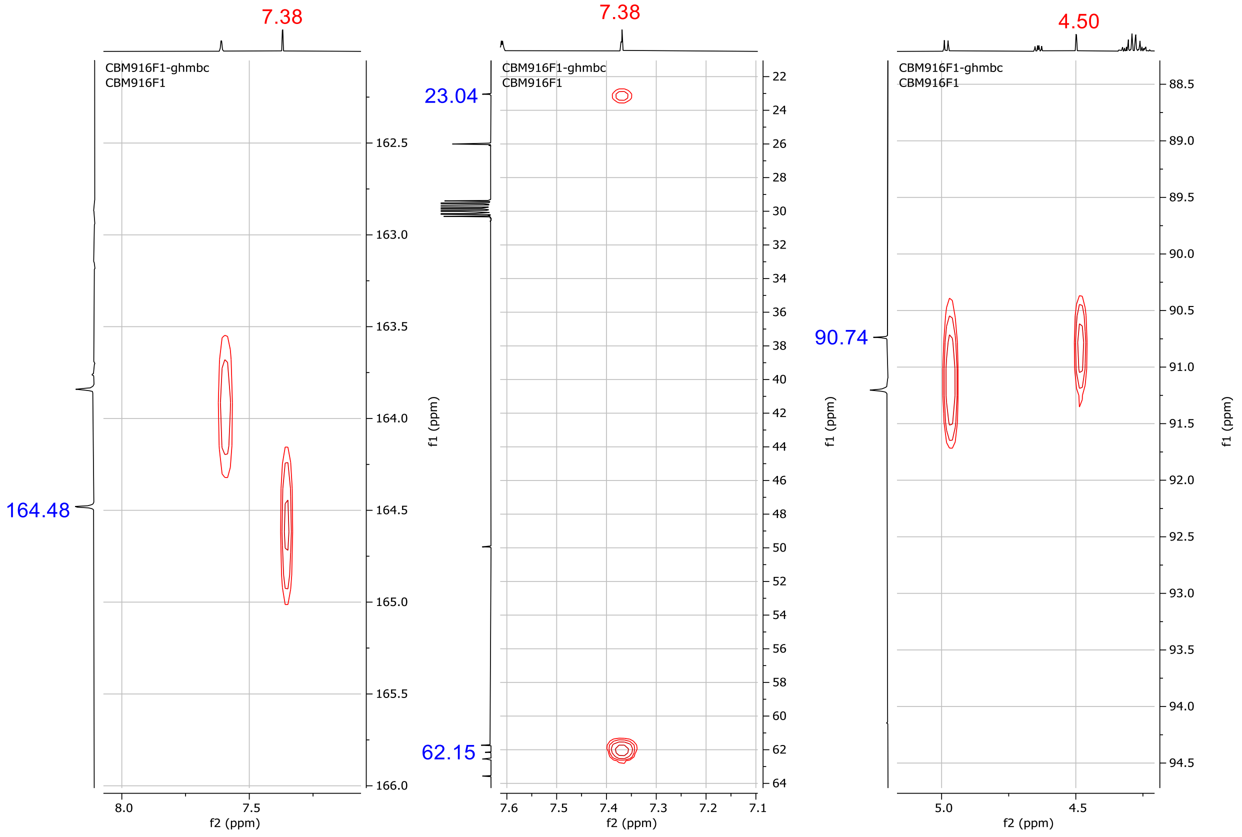

**FIGURE S38. Compound 11 (ROESY)**

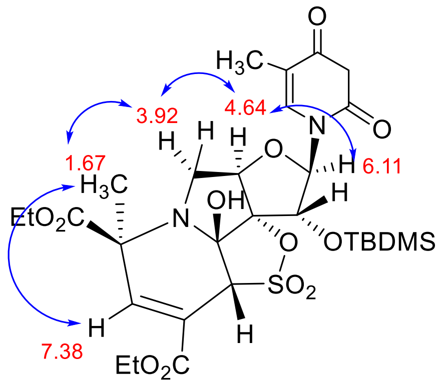


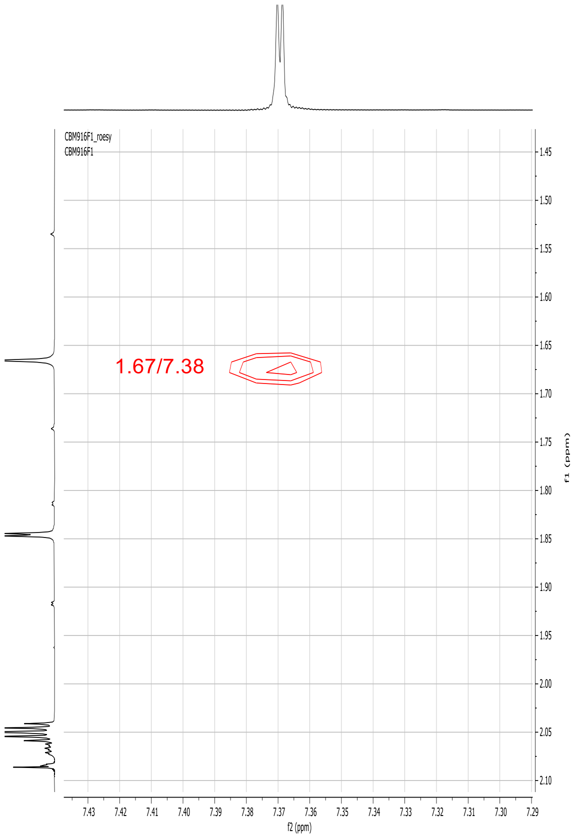


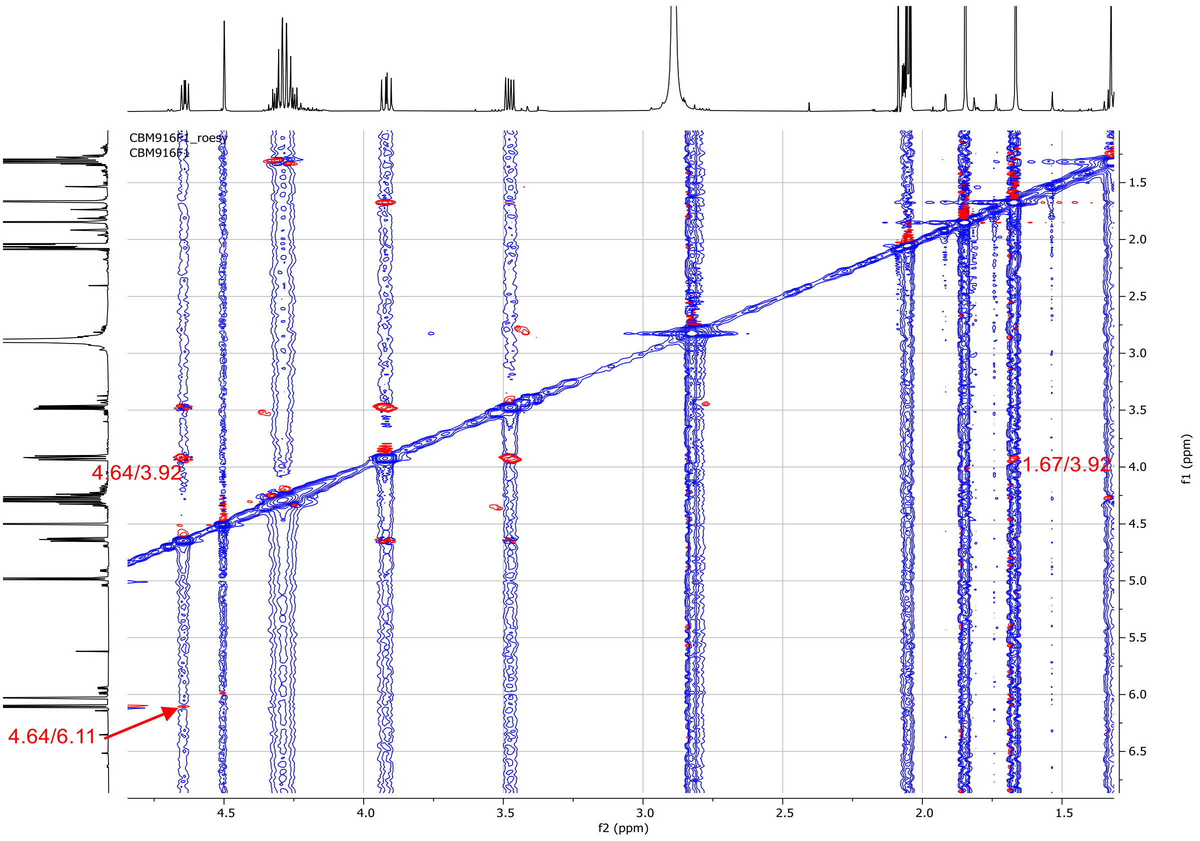

## FIGURE S39. Compound 12 ($^1$H-NMR)

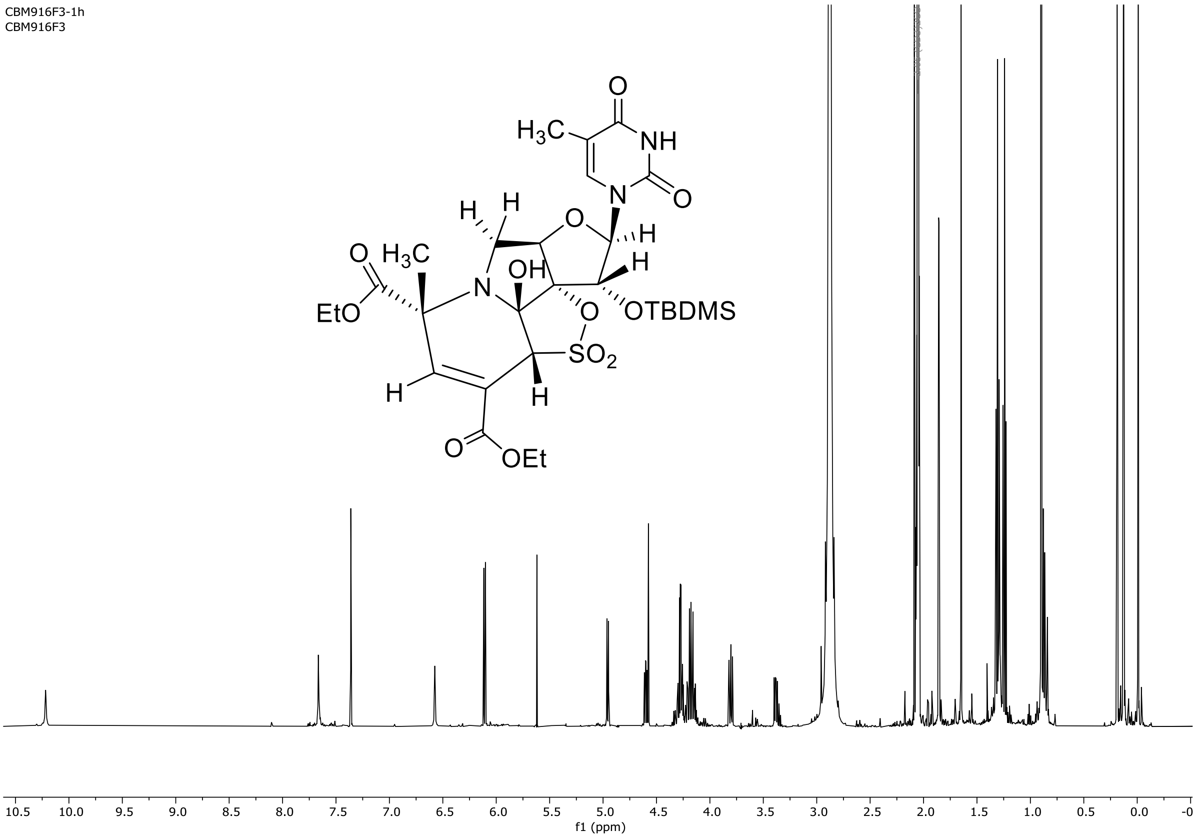


## FIGURE S40. Compound 12 ($^{13}$C-NMR)

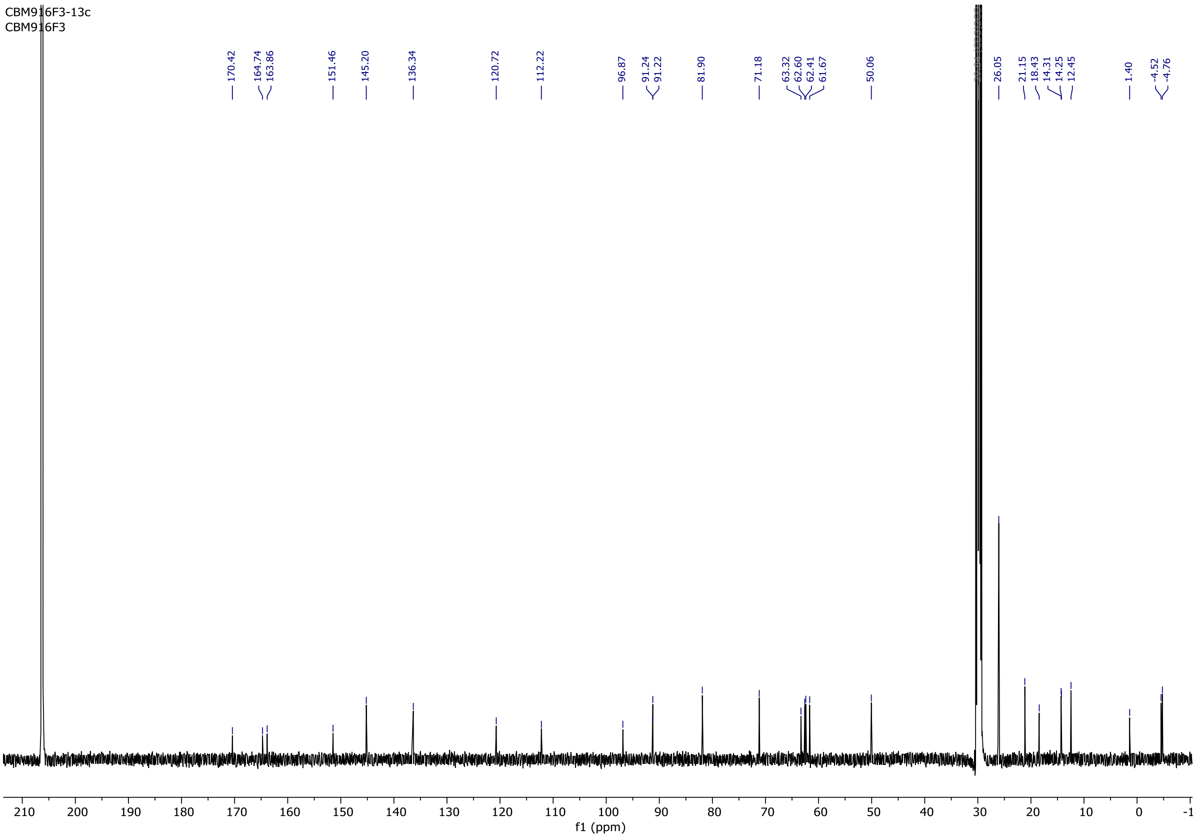

**FIGURE S41. Compound 12 (HMBC)**

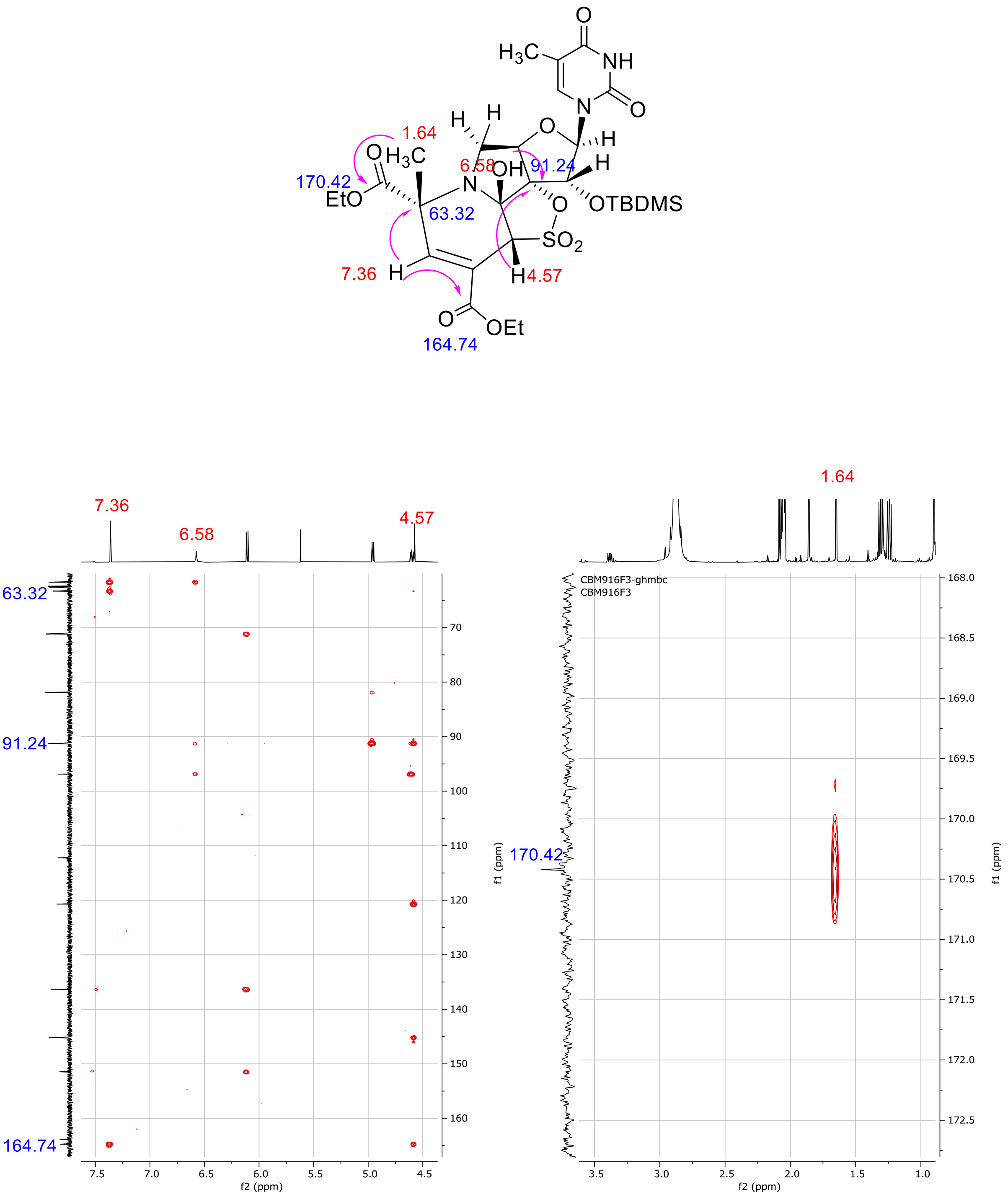

**FIGURE S42. Compound 12 (ROESY)**

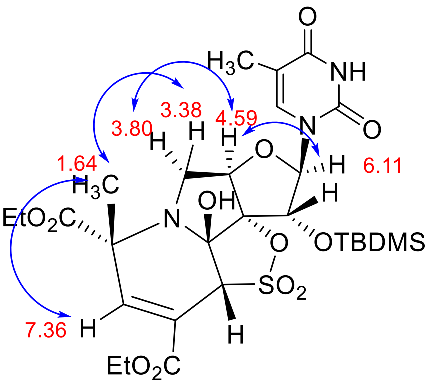


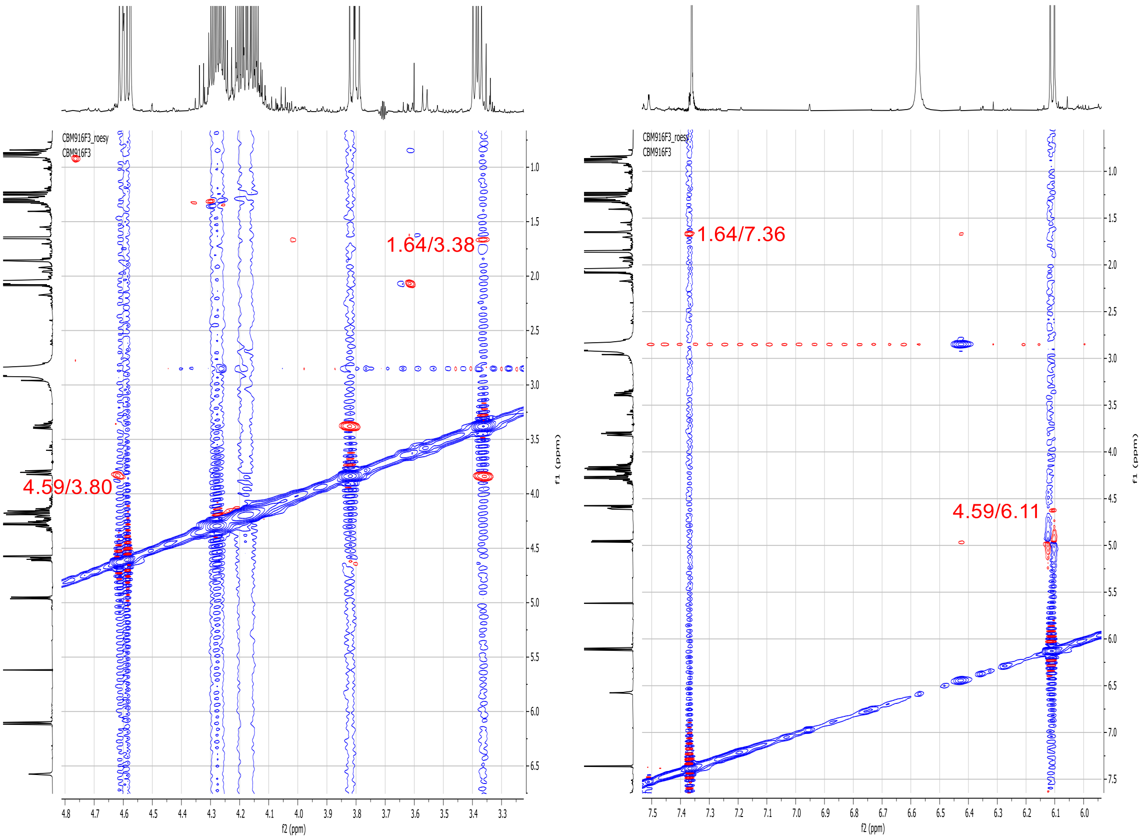

**FIGURE S43. Compound 13 ($^{1}H$-NMR)**

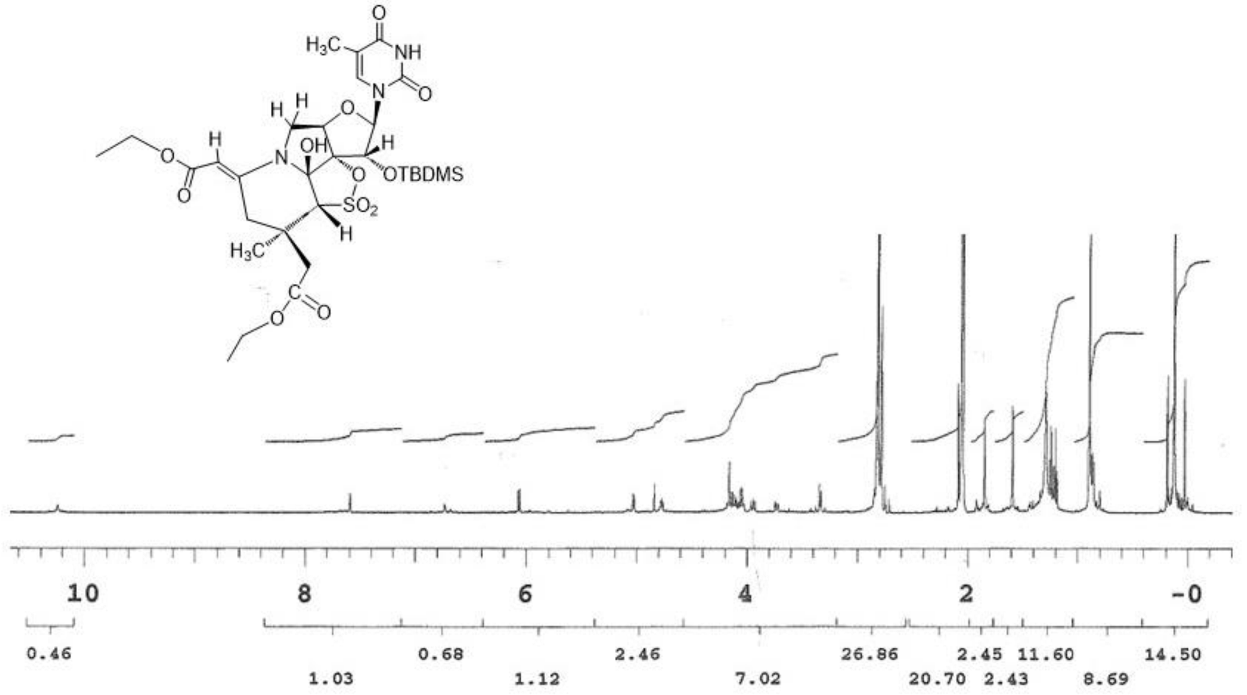


**FIGURE S44. Compound 13 ($^{13}C$-NMR)**

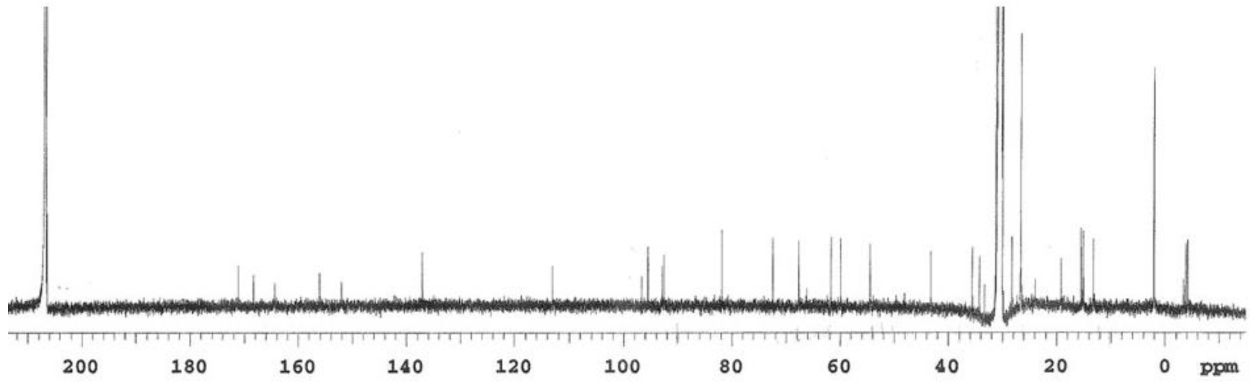


**FIGURE S45. Compound 13 (HSQC)**

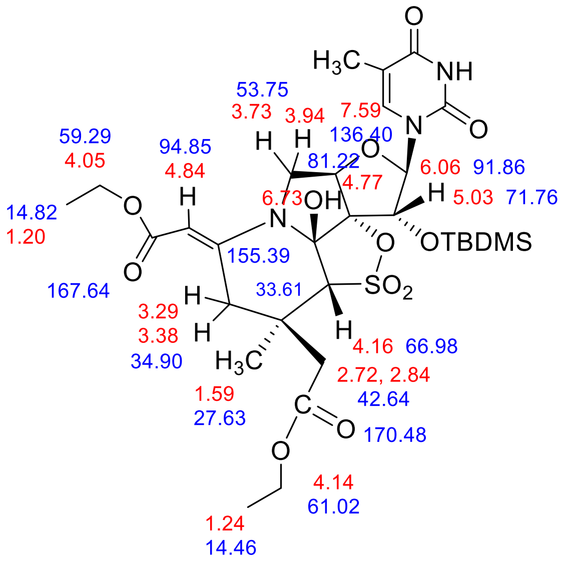

53.75
3.73
3.94
7.59
136.40
59.29
4.05
94.85
4.84
81.22
4.77
6.06
91.86
14.82
1.20
6.73
OH
5.03
71.76
OTBDMS
155.39
167.64
33.61
$SO_2$
3.29
3.38
34.90
$H_3C$
4.16
66.98
2.72, 2.84
42.64
1.59
27.63
170.48
4.14
61.02
1.24
14.46


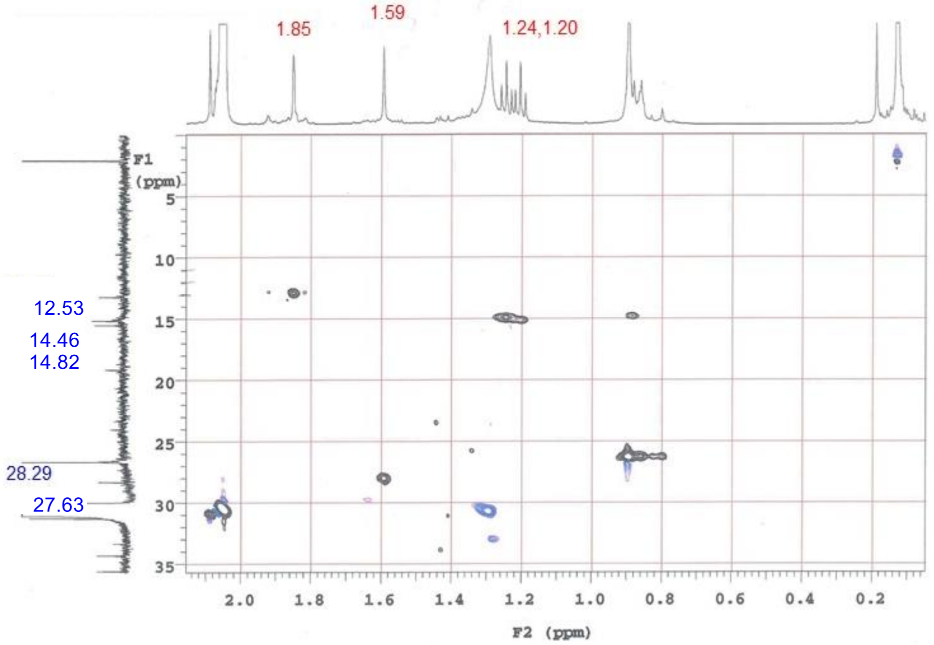

1.85
1.59
1.24,1.20
12.53
14.46
14.82
28.29
27.63
F1 (ppm)
F2 (ppm)


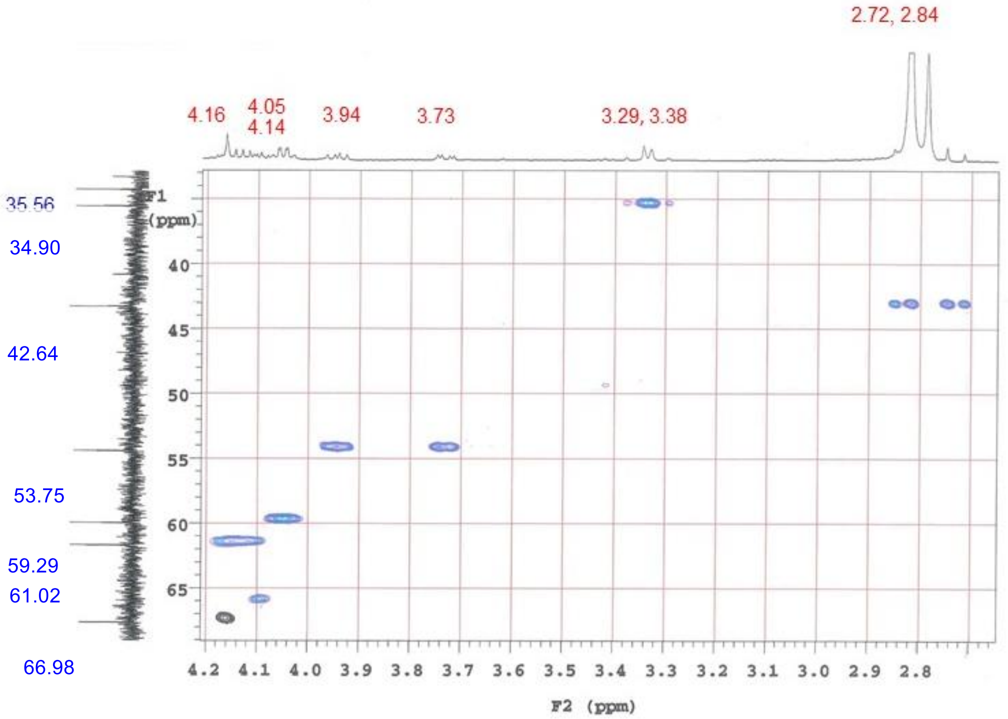

2.72, 2.84
4.16
4.05
4.14
3.94
3.73
3.29, 3.38
35.56
34.90
42.64
53.75
59.29
61.02
66.98
F1 (ppm)
F2 (ppm)

**FIGURE S46. Compound 13 (HMBC)**

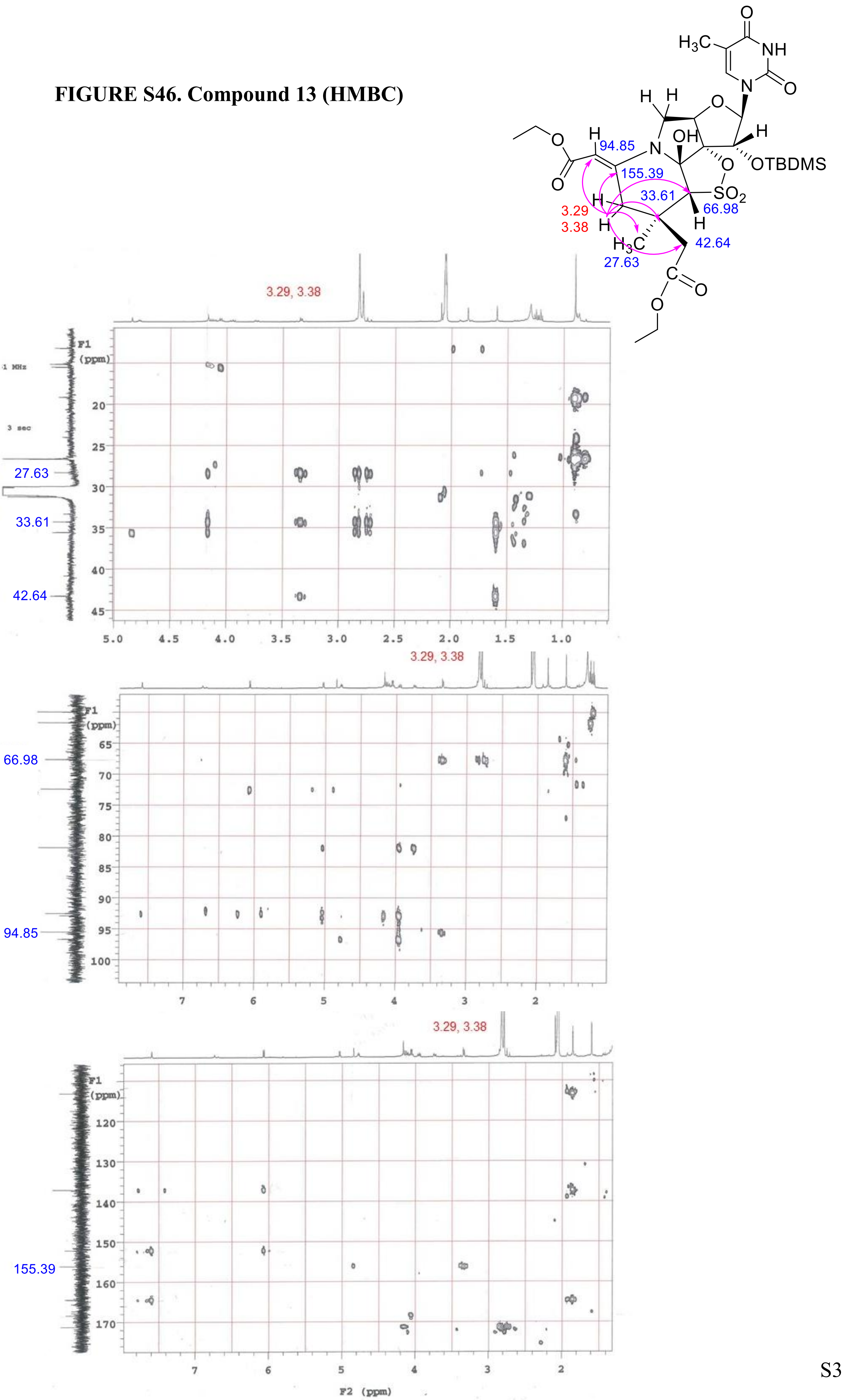

**Figure S47. Compound 13 (ROESY).** Correlation of the signal at $\delta$ 1.59 ppm ($CH_3$) with any other proton was not observed

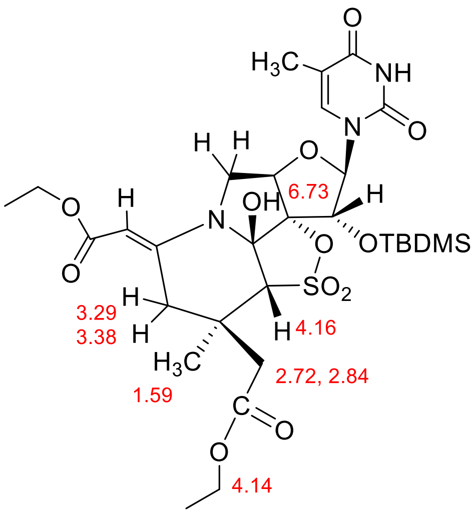


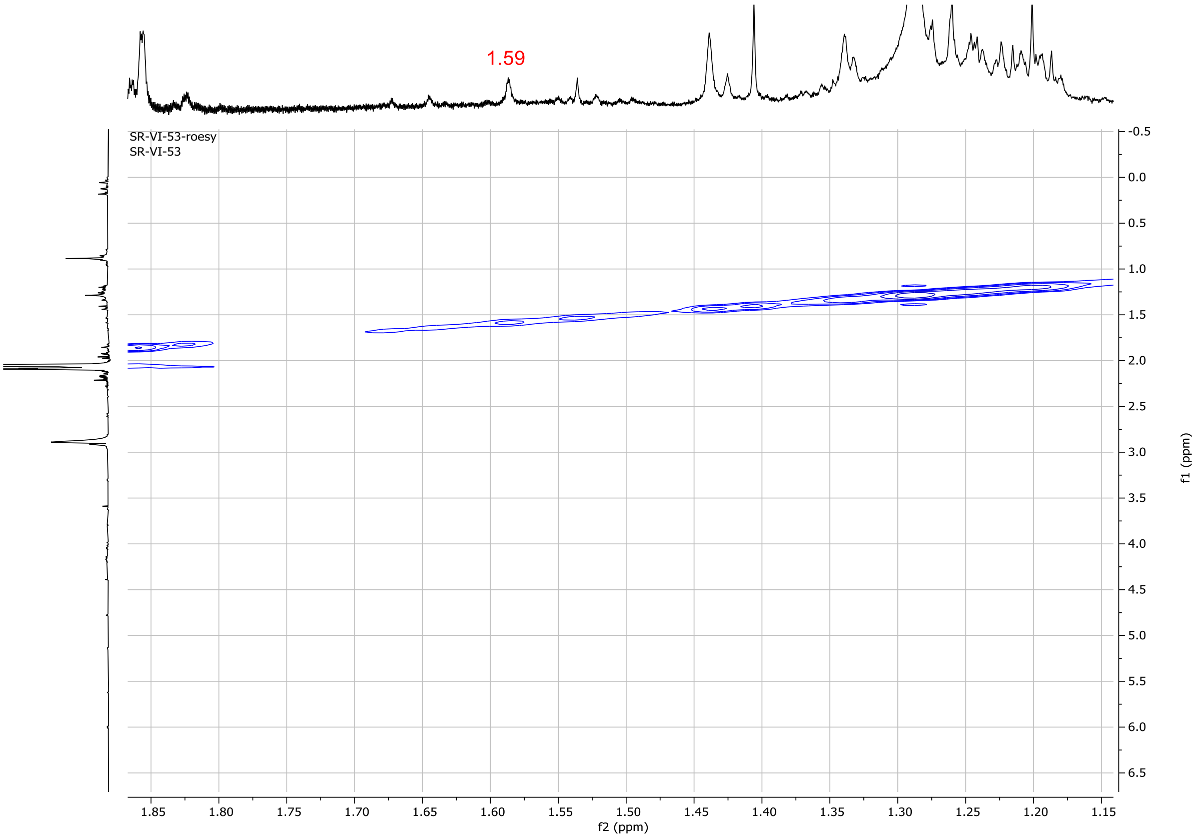

Supplement: Supplementary file 1 — ao4c02553_si_001.pdf [file ao4c02553_si_001.pdf]
